# Episodes of Rapid Recovery of the Functional Activity of the *ras85D* Gene in the Evolutionary History of Phylogenetically Distant *Drosophila* Species

**DOI:** 10.3389/fgene.2021.807234

**Published:** 2022-01-12

**Authors:** A. I. Chekunova, S. Yu. Sorokina, E. A. Sivoplyas, G. N. Bakhtoyarov, P. A. Proshakov, A. V. Fokin, A. I. Melnikov, A. M. Kulikov

**Affiliations:** ^1^ Evolutionary Genetics of Development, N.K. Koltzov Institute of Developmental Biology of the Russian Academy of Sciences, Moscow, Russia; ^2^ Department of Biochemistry, Molecular Biology and Genetics, Institute of Biology and Chemistry of Moscow Pedagogical State University (MPGU), Moscow, Russia; ^3^ Laboratory of Genetics of DNA Containing Viruses, Federal State Budgetary Scientific Institution «I. Mechnikov Research Institute of Vaccines and Sera», Moscow, Russia

**Keywords:** *Drosophila*, evolutionary conservative motifs, *ras85D*, transcription start site, broad peaked promoter, conversion, core promoter elements, non-coding regions

## Abstract

As assemblies of genomes of new species with varying degrees of relationship appear, it becomes obvious that structural rearrangements of the genome, such as inversions, translocations, and transposon movements, are an essential and often the main source of evolutionary variation. In this regard, the following questions arise. How conserved are the regulatory regions of genes? Do they have a common evolutionary origin? And how and at what rate is the functional activity of genes restored during structural changes in the promoter region? In this article, we analyze the evolutionary history of the formation of the regulatory region of the *ras85D* gene in different lineages of the genus *Drosophila*, as well as the participation of mobile elements in structural rearrangements and in the replacement of specific areas of the promoter region with those of independent evolutionary origin. In the process, we substantiate hypotheses about the selection of promoter elements from a number of frequently repeated motifs with different degrees of degeneracy in the ancestral sequence, as well as about the restoration of the minimum required set of regulatory sequences using a conversion mechanism or similar.

## Introduction

Bifurcation points on the phylogenetic tree of species often correspond to critical periods in the evolutionarily history of a population, resulting from abrupt changes in the environment and accompanied by physiological and genomic stress. At the genetic level, these events are associated with a disruption of genome stability and a sharp increase in the mutation rate (Galhardo et al., 2007; Malkova and Haber, 2012). The molecular mechanisms of mutagenesis caused by genomic instability are diverse: a weakening of the error repair by DNA polymerases and changes in the contribution of different polymerases to DNA replication, changes in the conversion mechanisms for the repair of deletions and double-strand breaks, an increase in the transposition activity of mobile elements, associated changes in the expression activity of the target genes of such transpositions and the formation of chimeric sequences, as well as large-scale rearrangements of the genome (Wong and Choo, 2004; Feschotte and Pritham, 2007; Garcia Guerreiro, 2012).

The transpositions of mobile elements can be accompanied by inversion events. The direct participation of mobile elements in the formation of rearrangements has been shown in *Drosophila* both in laboratory experiments (Alonso-Gonzalez et al., 2006; Kovalenko et al., 2006) and in evolutionary studies (Zelentsova et al., 1999; Evgen’ev et al., 2000). Such rearrangements lead to sequence replacements in the border region and to significant changes in regulatory sequences of downstream genes. The genome is literally saturated with the consequences of such rearrangements, which is manifested in a sharp decrease in the degree of gene colocalization on the synteny plots of chromosomes or Muller elements, as the phylogenetic distance between the compared species increases (Drosophila 12 Genomes Consortium and Clark, 2007). The consequences of rearrangements are manifested as a significant increase in linkage disequilibrium in their inner regions (Wallace et al., 2013) and in the level of polymorphism at the edges (Rozas et al., 1999; Sanchez-Gracia and Rozas, 2011). The authors associate the increase in the mutation rate in the latter case with the action of directed selection. Of interest are the following questions: the fixation rate of such consequences of genomic instability, the possible degree of disruption in the functional activity of genes bordering on insertions and rearrangements, and the influence of these events on further evolution of the local sequence.

Selection on coding sequences is in most cases associated with the maintenance or change of the functional activity of the protein encoded. The level and specificity of expression activity are associated with the variation of regulatory sites in non-coding sequences. For species with a distant relationship, a significant variation of key regions of the gene regulatory sequence—transcription start sites (TSSs)—has been shown. Using a large sample of loci from *Drosophila* species with different degrees of relationship, Main and co-authors have shown a high similarity of TSS sequences in orthologs for species of the *melanogaster* group and their significant differences from *D. pseudoobscura* (Main et al., 2013). The evolutionary shift of TSSs can supposedly be explained by an increased mutation rate and the fixation of mutations in these regions and is therefore a consequence of the effect of positive selection. Considering the significant number of chromosome rearrangements when comparing *D. pseudoobscura* and *D. melanogaster*, and the even larger number of degenerate remnants of mobile elements, it can be argued that at least some of these inconsistencies are associated with the emergence of sequences of independent evolutionary origin. The following question remains open: how and to what extent does the restoration of the expression activity of such genes occur?

The region of the *ras85D* gene (*Drosophila Ras1*) in *Drosophila* species with different degrees of relationship seems to be an interesting model for testing the hypothesis about the role of mobile elements and rearrangements in the accumulation of mutations in non-coding sequences. The *Ras* genes are a family of evolutionarily conserved genes encoding proteins of the group of small G-proteins (small GTPases) that play the role of “molecular switches” (Valencia et al., 1991; Rommel and Hafen, 1998; Rojas et al., 2012). The vast majority of signaling cascades dependent on *Ras* proteins are conserved (Slack et al., 2015) and typical for all eukaryotes. The coding sequence of the *ras85D* gene is under the influence of strict stabilizing selection (Chekunova et al., 2008) and is characterized by neutral polymorphism and low population variation (Gasperini and Gibson, 1999). At the same time, analysis of the nature of interspecific mutation accumulation in a fragment of this locus in closely related *Drosophila* species of the *virilis* group showed a significant impairment in the operation of the molecular clock (Kulikov et al., 2010). Rearrangements and insertions of mobile elements occurring in functionally significant regions of the genome must inevitably lead to dramatic changes in the activity and regulation of these regions. In the case of the *ras85D* gene, one of the edges of the large inversion is located on the pre-promoter region. Disruption of the expression activity of the *ras85D* gene, as one of the most important genes of the cell cycle regulation system, should have a significant effect on the viability of the cell and the organism as a whole. Significant structural changes in the promoter region of this gene are associated with the formation of lethal mutations and pronounced severe morphological disorders abnormalities. Thus, the insertion of mobile elements and regulatory fusion constructs into the intergenic region upstream of the *ras85D* transcription start site (TSS) or into the region of the non-coding 5′UTR sequence leads to the occurrence of lethal and deleterious mutations, female sterility, and impairments in the structure of neuromuscular junctions (Schnorr and Berg, 1996; Spradling et al., 1999; Prober and Edgar, 2000; Koh et al., 2002; Wu et al., 2009).

The objectives of this study are to test the hypothesis of the evolutionarily independent origin of the promoter region in *Drosophila* species with different degrees of relationship, to confirm the rather obvious assumption about the participation of mobile elements in the formation of different variants of this sequence, and to reveal the most probable mechanisms for restoring the functional activity of the gene. In the latter case, the question is divided into two parts as follows. 1) How is the new promoter selected if a part of the sequence including the promoter is replaced? 2) How is the minimum necessary set of enhancers selected and restored for an adequate expression regulation?

## Materials and Methods

### Research Objects

The *ras85D* gene with upstream intergenic region of 29 *Drosophila* species, including *Drosophila erecta, D. yakuba, D. sechellia, D. simulans, D. melanogaster, D. rhopaloa, D. ficusphila, D. biarmipes, D. takahashii, D. elegans, D. suzukii, D. eugracilis, D. obscura, D. pseudoobscura, D. persimilis, D. ananassae, D. bipectinata, D. kikkawai, D. serrata, D. willistoni, D. albomicans, D. hydei, D. grimshawi, D. navojoa, D. mojavensis, D. virilis, D. americana, Dorsilopha busckii, Scaptodrosophila lebanonensis* (the Genomes—NCBI Datasets database), and nine species of the *virilis* group, including *D. lacicola, D. montana, D. borealis, D. flavomontana, D. kanekoi, D. ezoana, D. littoralis, D. lummei, D. novamexicana* (Yusuf et al., in prep.) were used for the analysis. The boundaries of the fragment for the species with annotated genomes were revealed according to the GDV genome browser data. The homologous sequences in unannotated genomes were found using BLASTn or tblastn with the *D. melanogaster ras85D, Rlb1* and *CG31344* coding or amino acid sequence. The boundaries of non-coding sequences were revealed by the SRA-BLAST results according to the Sequence Read Archive Database NCBI (if any), or by alignment with the sequences of most related species. To find sequences in *D. virilis* genome that are homologous to inverted repeats, specific for the *ras85D* upstream intergenic region of the *virilis* group of *Drosophila*, we used Nucleotide BLAST with fragment size of at least 75 bp.

### Sequence Alignment and Extraction of Evolutionarily Conserved Motifs

The sequence alignment was carried out in MEGA X software (Kumar et al., 2018) using the ClustalW method and manually for the inner area of the intergenic region of the *virilis* species group enriched with multiple deletions. Because of different evolutionary origin of noncoding sequences (intergenic region and 5′UTR of *ras85D*) of different *Drosophila* species groups, the alignment was carried out only for closely related species and for fragments with significant homology. Evolutionarily conserved motifs (ECMs) were used as markers of homology of noncoding regions. ECMs were identified in the sequences of all species studied using the MEME analytical platform version 5.4.1—the MEME (Bailey and Elkan, 1994; Bailey et al., 2009) and the MAST (Bailey et al., 2009) tools. ECMs were searched independently for two sets of sequences: Set A includes 23 species from different groups, excluding closely related species that demonstrate high homology of noncoding regions; set B includes all 37 species. When the total amount of ECMs per sequence was 14, the number of common ECMs for both sets of sequences was maximal.

The number of homologous motifs in each sequence was set as any. In order to normalize for biased distribution of nucleotides, the background model was used by default. The homologous regions within the intergenic sequence of each species, such as forward and inverted repeats, were revealed using the YASS tool (Noe and Kucherov, 2005). The parameters of sequence fragment comparison were used by default.

### Revealing of the Sequence Homology With Mobile Elements

The search for regions of homology with mobile elements was carried out using the GIRI Repbase library and the Censor tool (Kohany et al., 2006) with the Hexapoda filter (Insects). The Censor tool does not allow to decrease the Score values and to change the values of false positive homology (E-values). Because of that, the search for homology with degenerate motifs was performed using the YASS tool (Noe and Kucherov, 2005) and the library of sequences of 57 transposon superfamilies from 52 *Drosophila* species of total amount—2280 records.

### Analysis of the Promoter Structure

To determine the TSS of the *ras85D*, we used the publicly available NCBI databases: SRA (for cDNA libraries), EST, Gene, TSA. The enrichment of the promoter region with promoter elements was estimated using the AME algorithm (McLeay and Bailey, 2010), letter-probability matrix (LPM) of JASPAR POLII motifs library for core promoter elements (Wasserman and Sandelin, 2004; Stormo, 2013) and LPM calculated on the base of positional weight matrices (PWM) for Ohler elements (Rach et al., 2009). Control sets of sequences were obtained in two ways: by cutting fragments of similar length from the upstream region of the intergenic sequence in species that form groups with common pattern of promoter elements (user-provided control sequences), and by generating random permutations based on the composition of the analyzed sequences (shuffled input sequences). To analyze small or highly heterogeneous samples of promoter sequences in the *obscura*, *ananassae*, and *montium* groups the Xstreme algorithm was used (Grant and Bailey, 2021). To visualize the data FIMO algorithm was applied (Grant et al., 2011) using all the identified motifs for each set of sequences and lowering the match *p*-value to 0.01. The obtained coordinates and structures of motifs for each fragment of the sequence with the putative promoter were mapped on the aligned sequences in the MEGA X software and were visualized on the scheme using coordinates of the general consensus sequence.

### GO Enrichment Analysis

Estimations of the enrichment of ECMs with the binding sites of transcription factors (TF) and with promoter elements were obtained using the AME algorithm. LPMs from Combined *Drosophila* Databases represented by the MEME SUITE tool were used as a library of motifs of TF binding sites. The results obtained were filtered in accordance to the list of TFs confirmed for the *ras85D* locus of *D. melanogaster* presented in the NCBI and modENCODE genome browsers (Kudron et al., 2018). GO enrichment analysis was performed using the FlyEnrichr Database (Chen et al., 2013; Kuleshov et al., 2016).

### Phylogenetic Analysis and Timetree Construction

Phylogenetic constructions and timetree calculations were performed using the MEGA X software. The Maximum Likelihood method was used with the Tamura 3-parameter model (Tamura, 1992) for the coding sequences of the *ras85D* gene of 38 *Drosophila* species including *Scaptodrosophila lebanonensis* as an outgroup. Gamma distribution was used to model evolutionary rate differences among sites [5 categories (+G, parameter = 0.8107)]. The rate variation model allowed for some sites to be evolutionarily invariable [(+I), 19.28% sites]. A timetree was inferred by applying the RelTime method (Tamura et al., 2018). Divergence times for all branching points in the topology were calculated using the Maximum Likelihood method and Tamura-Nei model (Tamura and Nei, 1993). This analysis involved 28 nucleotide sequences. The estimated log likelihood value of the topology shown is −4498.05. A discrete Gamma distribution was used to model evolutionary rate differences among sites [3 categories (+G, parameter = 3.0235)]. The timetree was computed using 3 calibration constraints. Estimates of the divergence time of *D. lummei—D. novamexicana* (2.9 Ma), species of the *montana* subgroup (4.8 Ma), and all species of the *virilis* group (9–9.5 Ma) were chosen as calibration (Morales-Hojas et al., 2011).

## Results

### Evolutionarily Conserved Motifs of the Promoter Region and 5′UTR Sequence of Different *Drosophila* Species

**FIGURE 1:**
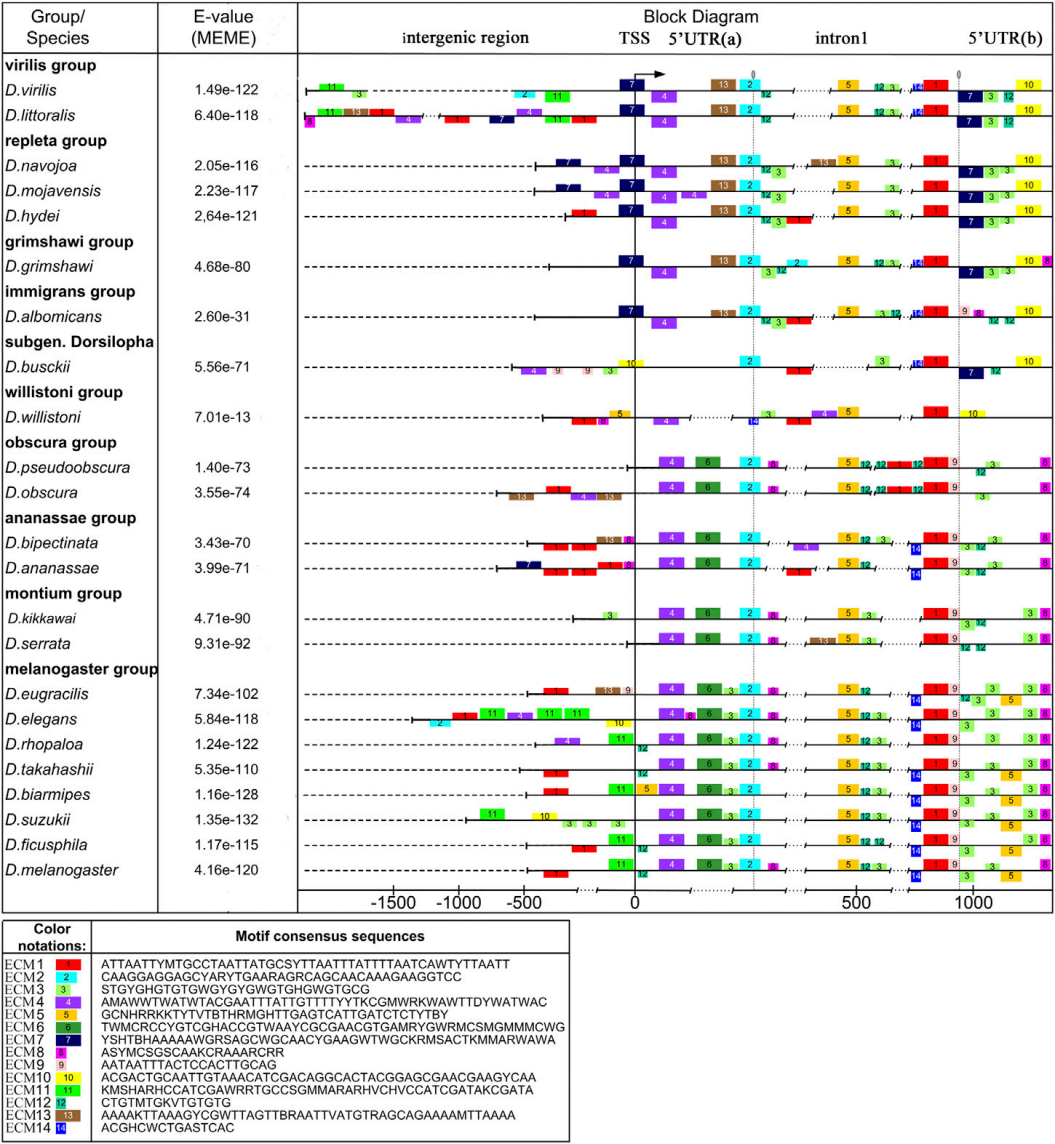
Polymorphism of the intergenic region, promoter, 5′UTR, and intron 1 in *Drosophila* species with different degrees of relationship. Scheme of the distribution of evolutionarily conserved sequences and insertion-deletion polymorphism in the analyzed region of the *ras85D* gene. The structure of evolutionarily conserved sequences which were obtained using the MEME algorithm. In the scheme, the boxes above the line show the location of ECMs on the plus strand, while the boxes below the line show ECM location on the minus-strand. The reduced box height marks degenerate ECMs. The upper part shows species of the subgenus *Drosophila (D. virilis—D. albomicans),* and in the lower part, there are species of the subgenus *Sophophora (D. willistoni—D. melanogaster). D. busckii (*subgenus *Dorsilopha)* is located between the subgenera *Drosophila* and *Sophophora*.

The locus of our interest consisting of the *ras85D* upstream intergenic region and 5′UTR, subdivided into two parts by intron 1 contains cis-regulatory elements of *ras85D*. The formal criterion for the common evolutionary origin is a significant sequence homology obtained by alignment. The use of standard sequence alignment algorithms (Unipro UGENE v. 36, Rose et al., 2019; MEGA X, Kumar et al., 2018) made it possible to identify significant areas of homology only in closely related species, for example, in the *melanogaster* and *virilis* groups. For the sequences of all 37 species used in the analysis, it was possible to show the homology of small regions flanking intron 1, the 3′end of intron 1, and the 5′UTR region at the border with exon 1, containing different sets of deletions. This result was confirmed and supplemented using the MEME and MAST algorithms for finding evolutionarily conserved motifs (Bailey and Elkan, 1994; Bailey et al., 2009). The boundaries of ECMs and the significance of homology depend both on the degree of their similarity and on their total number in the total set of sequences. Adding closely related species with high sequence homology to the analysis increases the chances of identifying regions that are overrepresented in these sequences. Therefore, the search for ECMs was carried out in two versions: for 23 species of *Drosophila* from different phylogenetic clades (Set A) and for 37 species, including closely related species from the *melanogaster* and *pseudoobscura* subgroups and the *virilis* group (Set B). The results of the sequence analysis of the 23 species are presented graphically in Figure 1, and those for the 37 species are provided in the Supplementary Materials (Supplementary Figure S1). In the figures, the sequences are aligned from the promoter to exon 1. The species of the subgenera *Drosophila* and *Sophophora* are grouped in the upper and lower parts of the scheme, respectively; the species *D. busckii*, a representative of the subgenus *Dorsilopha*, is located between them. The values of the statistical significance of ECM patterns for each sequence are given, estimated after their extraction and mapping by the MEME algorithms and after additional alignment by the MAST algorithms. In accordance with these values, only the *D. willistoni* sequence has low, albeit significant, E-values, because of the degeneracy of most of the identified ECMs in this species. For each dataset, 14 ECMs were obtained. Their names correspond to the ordinal numbers of each set, assigned in accordance with the results of the two-tiered significance analysis of the motifs selection.

A number of the identified conserved motifs are typical for both versions of the analysis, not exactly coinciding, but showing significant similarity. In the schemes shown in Figure 1 and Supplementary Figure S1, they occupy coinciding or overlapping positions relative to the boundaries of the structural elements of the gene. The accordance of their ordinal numbers to each other is given in Table 1. A comparison of sequence logos is given in Supplementary Figure S2. The overlap of ECMs sets obtained from both sets of sequences allows revealing the ECMs that are evolutionarily conservative and most likely highly significant, regardless of whether they have a common origin. Similar patterns of the ECMs distribution were obtained for both sets of sequences (for 24 and 37 species) when the fewer expected ECMs were selected in the settings. However, the best ECM topological match was obtained with the selection of 14 expected ECMs.

**TABLE 1:** Homology of evolutionarily conserved motifs isolated from the Set A (37 species) and the Set B (23 species) of the *ras85D* promoter region sequences.

Set A (23 sp.), conservative_sequence	Strand	Set B (37 sp.), conservative_sequence	Strand
ECM2 (E = 3.6e-257)	+	ECM1 (E = 1.4e-734)	+
ECM1 (E = 2.7e-315)	+	ECM2 (E = 1.3e-738)	+
ECM3 (E = 1.2e-234)	−	ECM3 (E = 1.8e-356)	+
ECM12 (E = 2.4e-028)	−	ECM3 (E = 1.8e-356)	+
ECM4 (E = 7.9e-135)	+	ECM4 (E = 7.9e-296)	+
ECM4 (E = 7.9e-135)	+	ECM13 (E = 4.7e-197)	−
ECM5 (E = 1.3e-109)	+	ECM5 (E = 2.8e-441)	+
ECM7 (E = 9.7e-069)	−	ECM8 (E = 3.5e-220)	+
ECM7 (E = 9.7e-069)	+	ECM9 (E = 1.9e-294)	+
ECM4 (E = 7.9e-135)	−	ECM7 (E = 3.7e-271)	+/−*
ECM13 (E = 3.8e-026)	+	ECM11 (E = 1.2e-257)	+

*motif 7 from Set B is represented by a degenerate palindromic sequence homologous in the 5′-region to a fragment of motif 4 from the minus strand from Set A, and in the 3′-region to the same fragment from the plus strand.

When specifying the ECM number, hereinafter we will mean the ordinal numbers of the motifs obtained by analyzing the set of sequences from the 23 species, unless otherwise indicated. ECM1 – ECM5 and ECM12 are present in all the species. ECM3 and ECM12 are represented by fragments enriched with AC-dinucleotide repeats and are located in the middle part of intron 1 on the plus strand and in the second half of the 5′UTR both on the plus strand and on the minus strand. The distribution of ECM1, ECM2, ECM3, and 5 suggests that the area of intron 1 and the 5′UTR region, from the small fragment flanking the intron upstream and corresponding to ECM2 to exon 1, has a common evolutionary origin in all the species. It can be seen that this area has numerous deletions and possible degenerate duplications of sequence fragments. ECM4, located near the TSS, is present in all the species, but its location differs among species from different *Drosophila* subgenera. In species of the subgenus *Drosophila*, this motif is located on the minus strand and borders on ECM7, which is located in the promoter region and is specific to these species. ECM7 is also present in these species immediately behind the intron region on the opposite strand. In species of the subgenus *Sophophora*, ECM4 is located on the plus strand and is displaced by 49 bp downstream of the TSS. An exception is the species *D. willistoni*, which diverged earlier than the others from the common ancestor of the presented species of the subgenus *Sophophora*: in *D. willistoni*, a degenerate ECM4 sequence is also identified on the minus strand, as in the species of the subgenus *Drosophila*. In *D. busckii*, a representative of the subgenus *Dorsilopha*, a degenerate form of this motif is displaced upstream of the TSS and is located on the minus strand at the beginning of the intergenic region (Figure 1). The lack of homology of the left 5′UTR fragment in the species of the subgenera *Drosophila* and *Sophophora* is also confirmed by the fact that this region features ECM6, which is unique to *Sophophora*, and ECM13, which is unique to *Drosophila*.

The intergenic region is the most variable of all, its size varies from more than 2000 bp in species of the *virilis* group to an almost complete absence in a number of species of the *obscura* group (*D. pseudoobscura*, *D. persimilis*) and in *D. serrata* from the *montium* group. Sequence homology can be detected only in selected groups of species with significant phylogenetic similarity. Thus, ECM11 was found in species of the *melanogaster* group of the *melanogaster*, *rhopaloa*, *ficusfila*, and *suzukii* subgroups. The position of ECM11 can be significantly shifted relative to the promoter, in particular, due to insertions and the multiplication of AC-repeats in ECM3, as in *D. suzukii*. In the subgenus *Drosophila*, ECM11 is found only in the *virilis* group: it is located on the minus strand distally of the promoter. All the other ECMs of the intergenic sequences identified in species from different phylogenetic clades are represented by degenerate copies.

The general picture of the variation of the entire non-coding sequence suggests that the main role in the regulation of the *ras85D* gene expression is played by the motifs located in the 5′UTR and the intron 1 regions. Even when there is rearrangement in this area, the ECM4 regains its position.

### The *ras85D* Gene Neighbourhood and Rearrangements

The *ras85D* gene is located in the Muller E element, which corresponds to chromosome 2 in *Drosophila* of the *virilis* group (Mitrofanov et al., 2011) and the right arm of chromosome 3 in *Drosophila* of the *melanogaster* group. The ras85D gene in *D. virilis is inverted in relation to neighboring genes-orthologs in D. melanogaster* (Supplementary Figure S3). As a result of the fixed chromosomal rearrangements, the closest gene neighbourhood of *ras85D* in species of the *virilis* group differs from that of all other *Drosophila* species. The genes located upstream of the *ras85D* gene in species of the *virilis* group [*GJ23372* (ortholog of the *D. melanogaster CG31344* gene), *GJ23373* (*D.m*. *Rpb7*) and *GJ23843* (*D.m. CG12241*)] are located at a distance of 5710 kB in the *D. melanogaster* genome (Supplementary Figure S3). The genes located in this place in other species (except for *D. willistoni*) are as follows: *Rlb1* (ortholog of the *GJ10856* gene in *D. virilis*), *mRpL47* (*D.v. GJ10316*), *JHDM2* (*D.v. GJ10857*), and *CG8176* (*D.v. GJ10858*), all of which are located at a distance of 680 Kb from the *ras85D* gene in the *D. virilis* genome (Supplementary Figure S3). The 3′-intergenic region also underwent rearrangement in the ancestor of the *virilis* group. There is an insertion of two genes between the 3′UTR of the *D. virilis ras85D* gene and the downstream set of genes typical for other species. One of them, *CJ23371* (ortholog of the *D. melanogaster AOX2* gene), is located at a distance of 6039 kB from the *ras85D* gene in the *D. melanogaster* genome. The second gene, *GJ26119*, is a chimeric gene that contains a fragment of the coding sequence of the retrotransposon *CR1-1* gene for pol-like protein with endonuclease and reverse transcriptase (RT) domains more than 2500 bp in length. This also points to frequent rearrangement events associated with the activity of transposons. The genetic environment characteristic of the species of the *melanogaster* group remains unchanged in other *Drosophila* species from both subgenera, for example, *D. pseudoobscura*, *D. ananassae*, *D. grimshawi*, and *D. mojavensis*. Only in *D. willistoni*, two genes—*Rlb1* and *mRpL47*—were reversed as a result of an inversion and are located on the opposite strand of the chromosome. Thus, the divergence of species is often accompanied by periods of genome instability and chromosomal rearrangements, which is associated with the activity of mobile elements (Hedges and Deininger, 2007).

### The Participation of Mobile Elements in Evolution of the *ras85D* Intergenic Region, Promoterand downstream 5’UTR sequence

To search for traces of mobile element insertions, we used the GIRI Repbase database and its software Censor tool (Kohany et al., 2006). The search was carried out along the entire length of the sequence from the putative TSS of the upstream gene to exon 1 of *ras85D*. The analysis results are shown in Table 2 and in more detail in the Supplementary Materials (Supplementary Table S1). The tables also include the search results for inverted repeats flanking some species-specific and species group-specific sequences. The analysis of repeats was carried out using the software tool YASS (Noe and Kucherov, 2005). The complete information on the analysis of the intergenic region in *Drosophila* of the *virilis* group is given in Supplementary Table S2. Non-random similarity with the sequences of mobile elements was found in 25 species of *Drosophila*, including 11 species of the *virilis* group.

**TABLE 2:** Homology with the transposon sequences of the *ras85D* noncoding and the upstream intergenic regions in *Drosophila* species.

Species	DNA transposon	LTR retrotransposon	Non-LTR Retrotr.
*D. willistoni*	*Homo5_hAT* ^IS^	*Gypsy1* ^P^	-
*D .pseudoobscura*	*P-1* ^IN^*	-	*L2-10* ^IN^*
*D. persimilis*	-	-	*L2-10* ^IN^*
*D. obscura*	*P-3* ^IS^, *Helitron-N1* ^IS^, *HelitronN-*1^IS^, In.rep^IS^	-	-
*D. suzukii*	*Homo10 hAT* ^IS^	*Gypsy-5* ^IS^, *Gypsy-23* ^IN^***	-
*D. takahashii*	*Hoana5/hAT* ^P^*	-	-
*D. eugracilis*	-	*invaider3-Gypsy* ^IS^, *Gypsy-11* ^IS^	-
*D. elegans*	*P-1N1* ^IS^, *piggyBac-N1* ^IS^, *Hoana5/hAT* ^P^*, *hAT-1* ^P^*	*Gypsy-1* ^IS^, *Gypsy-N1B* ^5’UTR*^	-
*D. ananassae*	-	*Gypsy1-LTR* ^IS^*, *Gypsy20-I* ^IS^*	-
*D. bipectinata*	*Transib-8* ^IS^*, *Transib-2* ^IS^*	*Gypsy-37* ^IS^*, *BEL-17* ^IS^*	-
*D. kikkawai*	*Transib-1* ^IS^	*Gypsy-24* ^IS^, *Gypsy-2* ^IS^	-
*D. busckii*	-	*Gypsy-1* ^IS^	-
*D. albomicans*	*Mariner-4* ^IS^	*Gypsy-24* ^P^***	**-**
*D. hydei*	-	-	*R1-4B* ^IS*^**
*D. mojavensis*	*hAT-2* ^IS^*	-	*Jockey-7* ^IS^*, *R1-3* ^IN^***
*D. grimshawi*	-	*BEL-7* ^IS^**	*CR1-1* ^IS^***
*Sp. gr. virilis*	Uncharacterized X –DNA TE In.rep.^IS^*	-	
*D. kanekoi*	*hAT-8* ^IS^*^V/K^, *Helitron-like-5* ^IS*V/K^	-	*L2-10* ^IN*^***
*D. ezoana*	*DNA8-78* ^IS^*^V/K^, *Helitron-N10* ^IS^*^V/K^	*Gypsy-22* ^IN^****	-
*D. littoralis*	*hAT-11* ^IS^*^V/K^, *Helitron-N10* ^IS^*^V/K^, *hAT-N36* ^IS^*^V/K^	*Gypsy-22* ^IN^****	-
*D. lummei*	-	*Gypsy-1* ^IS^*^V/K^, *Gypsy-22* ^IN^****	-
*D. virilis*	-	*BEL-10* ^IN^****	-
ancestral *virilis- kanekoi* s/gr.	*Polinton-2* ^IS^*^V/K^, *Helitron-N10* ^IS^*^V/K^, *Helitron-1* ^IS^*^V/K^	*Gypsy-22* ^IN^****	-

The superscripts indicate the region-specific position of the motif (IS, intergenic sequence; P, promoter, 5′UTR; IN, intron 1) and asterisks mark the homology of the aligned sequences at the level: *V/K—subgroups, *—own group of species, **—two related groups of species, ***—own Subgenus, ****—two related Subgenera.

Sequence elements of all the analyzed species showing homology with transposon sequences were excluded from the results of the analysis. Homology variants represented mainly by microsatellite repeats were also excluded. On the contrary, homology was considered characteristic of a group or subgroup of species if the consensus of a sequence element shared by the given group could be restored from fragments preserved in different species of this group.

**FIGURE 2:**
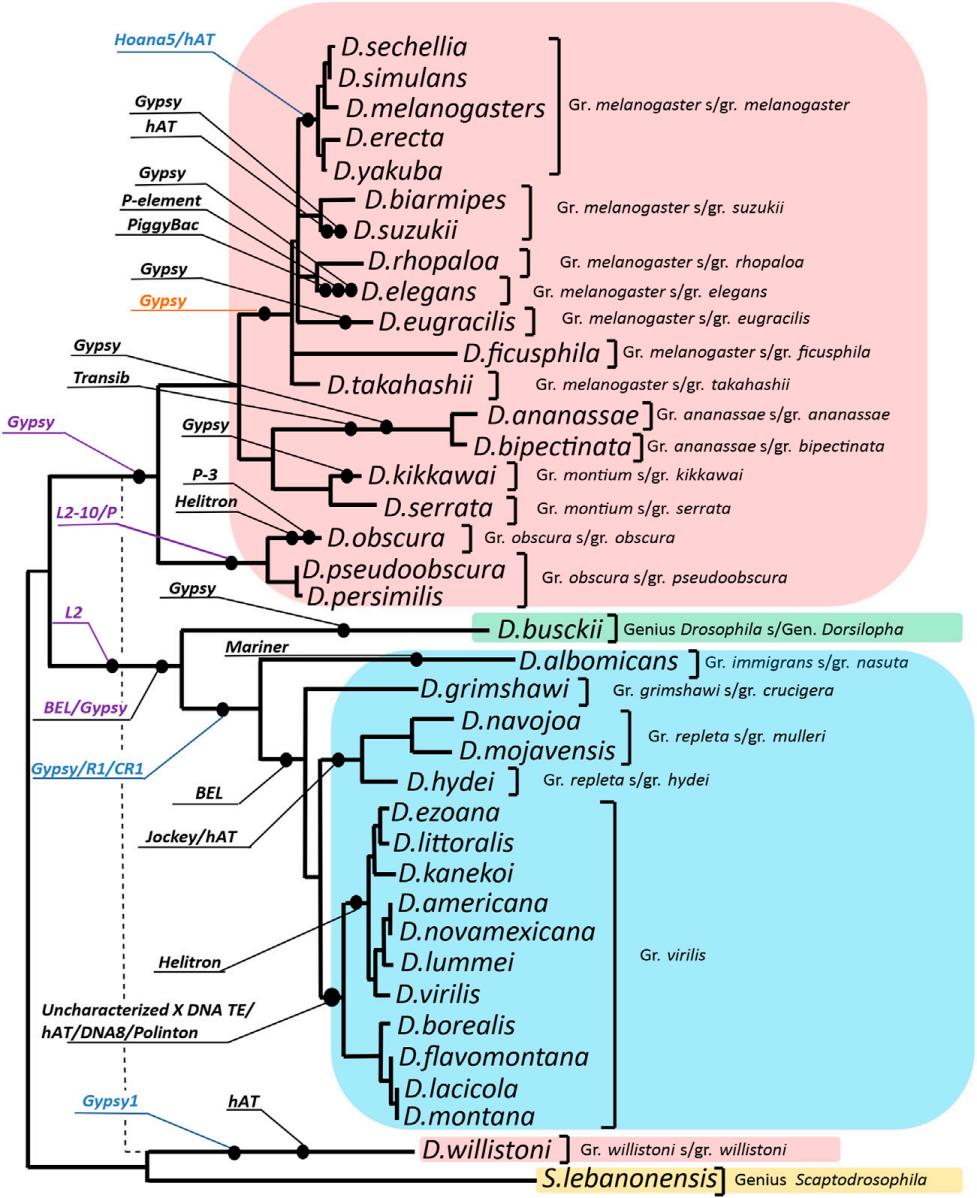
Distribution of the identified transposon insertion events on the evolutionary tree of the analyzed *Drosophila* species. The phylogenetic tree was constructed using the Maximum Likelihood method on the base of the Tamura 3-parameter model. Sequences of exons and 3′UTR of the *ras85D* gene of 37 *Drosophila* species were used. Gamma distribution was used to model evolutionary rate differences among sites [5 categories (+G, parameter = 0.8107)]. The rate variation model allowed some sites to be evolutionarily invariable [(+I), 19.28% sites]. The structure of the resulting phylogenetic tree is generally consistent with the species tree obtained for 155 genomes of *Drosophila* species (Suvorov et al., 2021) excluding *D. willistoni*, for which the position on the species tree is indicated by a dashed line. The callouts specify the transposable element and insertion events on the branches of the evolutionary tree. Callout-designated insertions in the following regions: intergenic region, promoter, 5′UTR, and intron 1, were highlighted in black, blue, purple, and orange, respectively.

According to the results, the following mobile elements may have participated in the formation of the evolutionary variation of the *ras85D* gene promoter region: DNA transposons belonging to the superfamilies *P, Helitron, hAT, Mariner, PiggyBac, Transib, DNA8*, and *Polinton*; LTR retrotransposons such as *Gypsy* and *BEL*; and non-LTR retrotransposons such as *L2, R1* and *CR1*. The events of transposon incorporation in the studied non-coding sequence during the evolutionary divergence of the *Drosophila* species are mapped on the phylogenetic tree (Figure 2).

The *ras85D* gene promoter region in species of the subgenera *Drosophila* and *Sophophora* is represented by three main patterns of ECMs location, all of which have independent origins. The 54 nucleotides before ECM4 in the species of the *montium*, *obscura*, and *ananassae* groups, which are part of the promoter, demonstrate a significant homology not only with each other, but also with a representative of the genus *Scaptodrosophila*, *S.lebanonensis*, which indicates the ancestral state of this region in these three groups of species. In this region, homology is found with the beginning of LTR retrotransposon BEL: this homology continues in the distal direction in *D. ananassae* and *D. bipectinata*. In the upstream part of the intergenic region, these species display homology with the terminal repeats of *Transib* DNA transposon and *Gypsy* LTR retrotransposon. In *D. pseudoobscura*, *D. persimilis*, and *D. serrata*, homology in this region is disrupted by large deletions, which may be a consequence of recombination of similar motifs of the mobile elements involved in the formation of this region. Thus, in *D. kikkawai*, traces of incorporation of a *Gypsy* group transposon are found in this region, while in *D. obscura*, inverted repeats flank the intergenic region and in the proximal part are located close to the region of common homology. These repeats are located in close proximity to or overlap with motifs characteristic of DNA transposon terminal repeats.

On the evolutionary branch of the *obscura* group, an extended insertion into intron 1 shows possible homology with either the 3′UTR region of an *L2* retrotransposon or with the terminal repeats of DNA transposon *P* (Supplementary Table S1). At the same time, in the species of the *pseudoobscura* subgroup, the homology with the transposon is more significant than in *D. obscura*.

For the species of the *melanogaster* group, according to the sequences of *D. takahashii* and *D. elegans*, it was possible to identify homology of the promoter region with inverted repeats of DNA transposons from the *hAT* superfamily. This points to a common evolutionary origin of the intergenic sequence, including the *ras85D* promoter, in most species of this group, by insertion of a mobile element prior to the divergence of the main subgroups of the *melanogaster* group. The noted variation of this region is related both to point mutations and to microindels. In species of the subgroups *melanogaster*, *ficusphila*, and *rhopaloa*, the short basal part of this insertion is deleted, about 10 bp. In the species *D. elegans*, *D. suzukii*, and *D. eugracilis*, later independent insertions of mobile elements belonging to the *P, hAT*, and *Gypsy* groups were noted in the area of the first insertion in the promoter region (Table 2). Moreover, in *D. eugracilis*, this led to the loss of the area of general homology with the other species (Supplementary Figure S4). The fragment of the intergenic region, which has a common origin in the species of this group, is polymorphic and has accumulated multiple deletions and point mutations in the distal part. In this case, the boundaries of deletions in different species do not coincide, which indicates independent events of sequence region loss. In phylogenetically more distant species of the *melanogaster* and *suzukii* subgroups, the area of homology accumulates deletions, so the common evolutionary origin of these regions is difficult to detect without prior alignment with other sister species.

In *D. willistoni*, the area of reliable homology of the non-coding sequence begins in the region of intron 1, but a small sequence fragment of 20 bp in length in the region of the promoter shows a strict homology with the species of the *montium*, *obscura*, and *ananassae* groups. The intergenic region in this species was formed as a result of an inversion rearrangement, and the most likely participant of this event is a retrotransposon related to *Gypsy*. Homology with the LTR of the retrotransposon is shown for the region of the *D. willistoni* sequence immediately behind the promoter region. In this case, the rearrangement completely replaced the ancestral sequence of the intergenic region and the 5′-part of the 5′UTR. The question of how the area of the promoter sequence showing homology with the species of the *montium*, *obscura*, and *ananassae* groups was preserved in this region remains open and will be discussed below.

**FIGURE 3:**
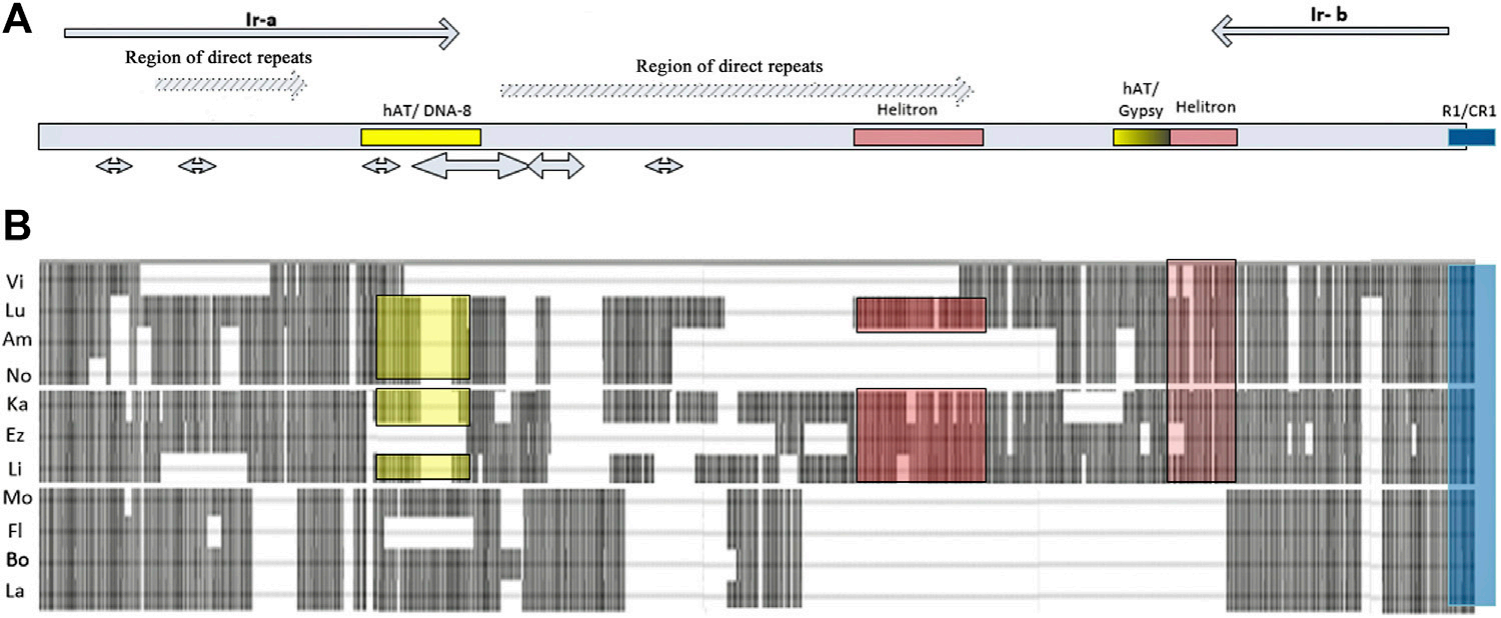
The structure of the *ras85D* upstream intergenic region in *Drosophila* species of the *virili*s group. **(A)** Repeats, palindromes, and areas of homology with mobile elements: inverted repeats flanking the sequence are designated as Ir-a and Ir-b and are represented in the scheme by gray arrows; regions of direct repeats are represented by light gray arrows with diagonal hatching; palindromes are shown by double-headed arrows; areas of homology with terminal repeats of transposons are marked by colored rectangles. **(B)** Insertion-deletion polymorphism of the regulatory region: rectangular gray blocks represent the DNA sequence, dashed lines and gaps between the blocks show the deletions.

In species of the subgenus *Drosophila* (*D. hydei* and *D. grimshawi*), ECM7 in the promoter region exhibits homology (65–70%) with the 5′UTR of non-LTR retrotransposons *R1* and *CR1.* Sequence differences appear in the region upstream of the promoter by 70 bp and lead to the formation of four independent patterns characterizing the species of the groups *virilis*, *repleta*, *grimshawi*, and *immigrans.* In *D. albomicans* (the *immigrans* group), this region shows homology with DNA transposon *Mariner*, in *D. grimshawi*, with LTR retrotransposon *BEL*, in *D. mojavensis*, there is a similarity with non-LTR retrotransposon *Jockey* or DNA transposon *hAT*. In species of the *virilis* group, an inversion rearrangement that affected the *ras85D* gene promoter region was accompanied by the appearance of inverted repeats flanking almost the entire intergenic region. Assessments of the position and structure of the repeats in 11 species of the *virilis* group, carried out using the YASS tool, indicate the fundamental similarity of the inverted repeats and their presence in all the species of this group (Supplementary Table S2). Both ends of the *ras85D* upstream intergenic sequence contain two to four fragments located sequentially and demonstrating significant homology of the inverted sequences (Score> 82; bit-score> 25.3; E-value <0.044). The alignment of the entire area of the intergenic region in species of the *virilis* group is shown in Figure 3. The region of direct repeats occupies the central part of the intergenic sequence. There is an individual direct repeats area in the middle part of the left inverted repeat (hereinafter referred to as Ir-a). As a result, Ir-a is 730 bp in length and turns out to be longer than the right inverted repeat (hereinafter referred to as Ir-b), which is 380 bp in length. Both repeats underwent various deletions in different species, as can be seen from the scheme of the arrangement of homologous regions in different species (Figure 3). The deletion mechanism of repeat divergence is confirmed by a significant homology of overlapping fragments of these repeats (Supplementary Figure S5).

### The Ancient Insertion of *Uncharacterized X DNA* Transposon and its Involvement in the Rearrangement of Chromosome 2 in the *Virilis* Group

**FIGURE 4:**
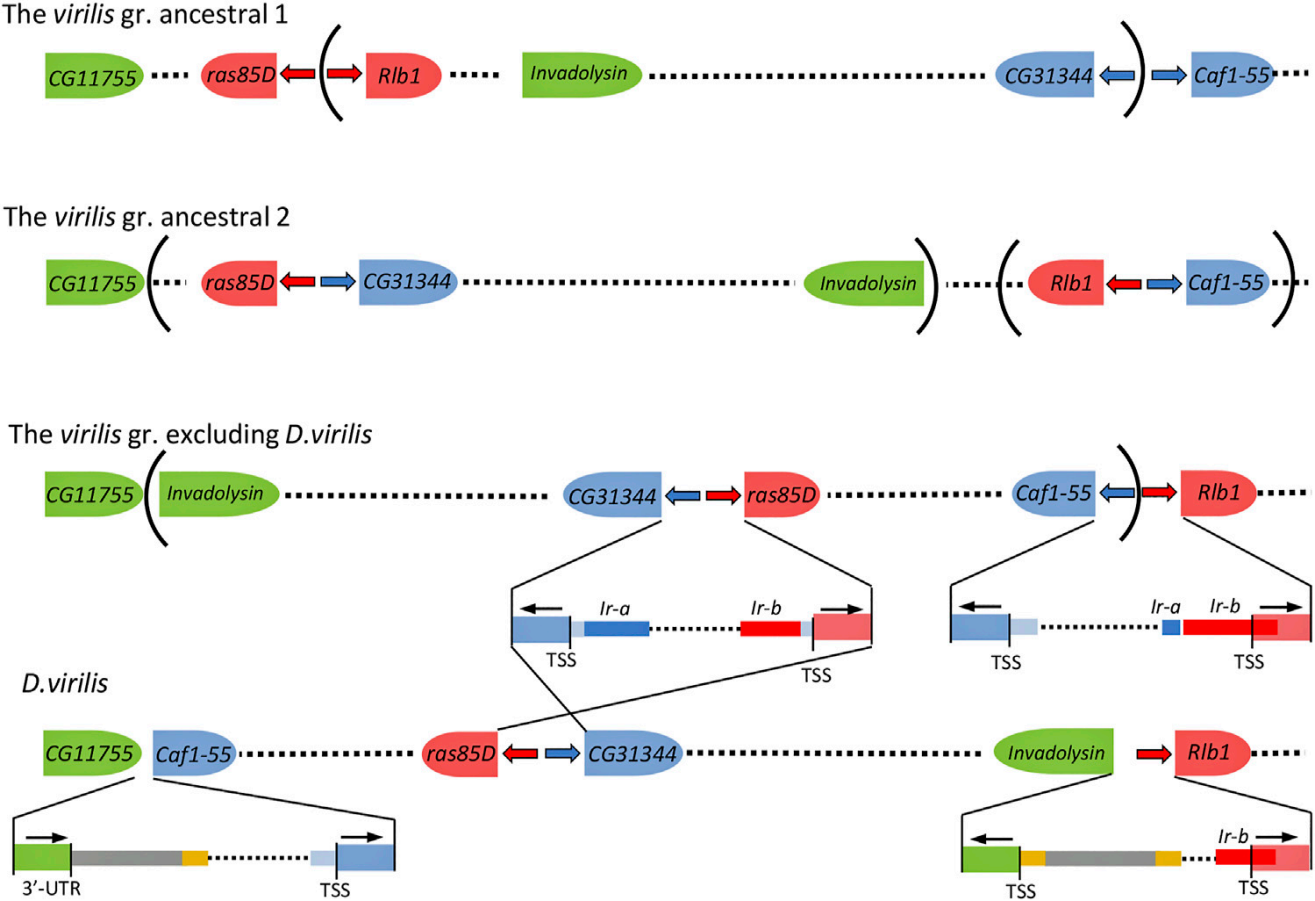
The minimal number of rearrangements required to transform the ancestral state of chromosome 2 to specific for the *virilis* group. Blue and red arrows—the inverted repeats Ir-a and Ir-b, respectively, that marked insertion sites of two copies of the Uncharacterized X DNA Transposon into the *ras85D—Rlb1* and *CG31344—Caf1-55* intergenic regions in the ancestor of the *virilis* species group. *DAIBAM* elements are colored with yellow (inverted terminal sequences) and gray (Reis, et al., 2018). Remnants of ancestral sequences are colored with light blue. The nomenclature of the genes corresponds to the names of *D. melanogaster* orthologs. The representation is not to scale.

There is no reliable homology of the inverted repeats of the *virilis* group with any known mobile element. However, in several species of the group, the 3′-end of Ir-a, 40–44 bp in length, resembles the terminal repeats of DNA transposons belonging to the *hAT, DNA8,* and *Polinton* superfamilies (Figure 2, Supplementary Table S1). In addition, numerous sequences of inverted repeats are found in the *D. virilis* genome. 37 degenerate sequences homologous to the inverted repeats Ir-a and Ir-b from closely related species of the *virilis* group have been found in the *D. virilis* genome by the BLAST method within different scaffolds. The length of these sequences ranges from 54 to 310 bp, while the length of 16 fragments exceeds 190 bp. (Supplementary Table S3). All the fragments are located in non-coding regions of the genome, and more than half of them have double confirmation either by homology with both repeats of the same species, or by homology with one of the repeats from two species. We have hypothesized that traces of inverted terminal repeats of some mobile element can be found along the boundaries of ancient rearrangements, including the rearrangement that led to a change in the gene environment of the *ras85D* gene in the ancestor of the *virilis* group, in regard to the gene order characteristic of other species in the subgenera *Drosophila* and *Sophophora*. To obtain a structure characteristic of the *virilis* group from the ancestral structure preserved in *D. melanogaster* and *D. grimshawi*, at least three inversions are required. One falls onto the *ras85D—Rlb1* and *CG31344—Caf1-55* intergenic regions, and the other two capture the regions of the formed *Rlb1—Caf1-55* and *ras85D—CG31344* blocks and turn them over into the opposite direction (Figure 4). In *D. virilis*, the order of genes in the area of chromosome 2 corresponding to scaffolds sc_13047 and sc_12855 differs from that of other species of the *virilis* group due to one more inversion (2A) described earlier (Reis et al., 2018), which occurred with the participation of DNA transposon *DAIBAM* (*hAT* superfamily) and implicated the same *Caf1-55—Rlb1* intergenic region on one side and the *invadolysin* gene on the other side.

As expected, the prepromoter region of the *D. virilis Rlb1* gene contains one of the inverted Ir-b repeat copies of 190 bp in length (sequence no. 5) (Supplementary Table S3), which exhibits the highest homology with the Ir-b repeat of *D. lacicola*. It occupies the area from the inverted repeat of *DAIBAM* to the TSS of the *Rlb1* gene and continues further into the region of the 5′UTR of the *Rlb1* gene. In the genomes of *D. montana* and *D. americana* that did not undergo the 2A rearrangement, the homology area with repeats is longer and consist of about 300 bp, i.e., more than 80% of the Ir-b repeat (Supplementary Table S4, Supplementary Figure S6).

Another copy, which should be located in the *Caf1-55* gene region and correspond to Ir-a, was not found in the *D. virilis* genome. However, there is a fragment from the right side of the Ir-a repeat of 107 bp in length in the central part of the *Caf1-55—Rlb1* intergenic region of the *D. americana* and *D. montana* genomes. This fragment is located upstream of the area of homology with the Ir-b element by 36 bp, but is oriented with its tail towards the *Caf1-55* gene (Supplementary Table S4, Supplementary Figure S6), i.e., in the direction opposite to the expected. A possible scenario of events leading to this result is considered in experiments with induced genomic instability with chimeric DNA transposons *P* in *D. melanogaster*, which have disruptions of inverted terminal repeats (Georgiev et al., 1997; Pomerantseva et al., 2006). Activation of transposase in this case leads to the formation of complete or partial deletions of the copies of transposons and downstream sequences, and to insertions of inverted sequence fragments. It can be concluded that the change in the *ras85D* upstream intergenic region in *Drosophila* of the *virilis* group occurred under the influence of insertion of some DNA transposon (uncharacterized *X* DNA TE) with inverted terminal repeats and subsequent inversion is associated with this insertion.

**FIGURE 5:**
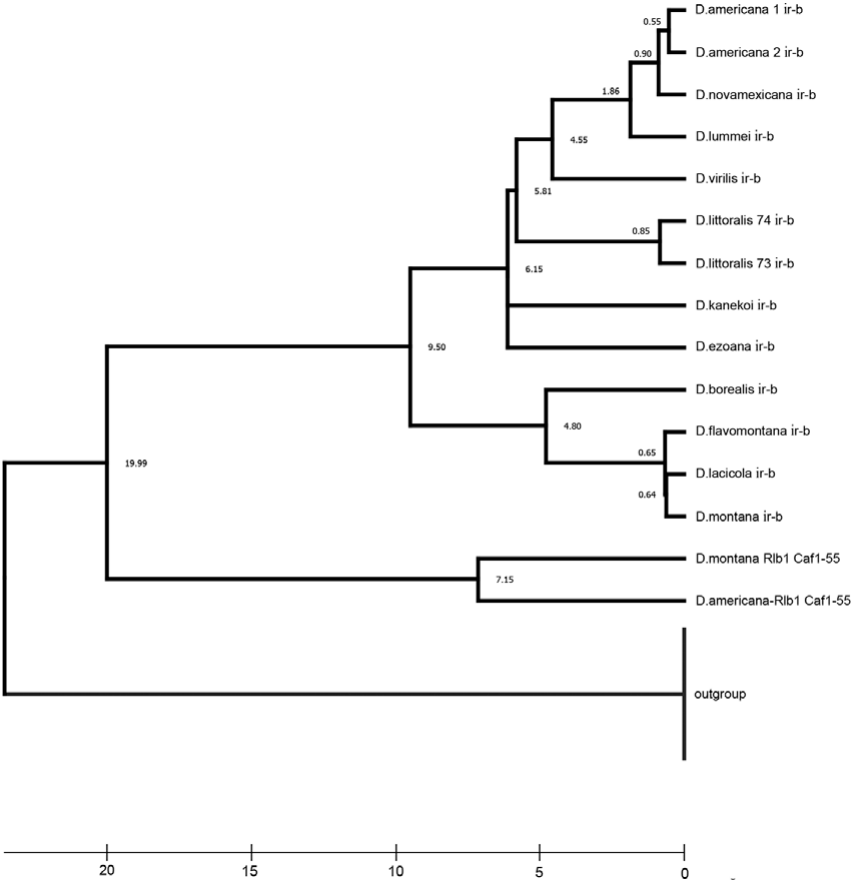
Estimated time of the ancestral rearrangement occurrence, based on the time of mutation accumulation of homologous Ir-b repeats. The Timescale at the bottom of the figure is in millions of years. A timetree inferred by applying the RelTime method (Tamura et al., 2012). The timetree was computed using three calibration constraints. This analysis involved 28 nucleotide sequences. Evolutionary analyses were conducted in MEGA X (Kumar et al., 2018). Divergence times for all branching points in the topology were calculated using the Maximum Likelihood method and Tamura-Nei model (Tamura and Nei, 1993). The estimated log-likelihood value of the topology shown is −4498.05. A discrete Gamma distribution was used to model evolutionary rate differences among sites [three categories (+G, parameter = 3.0235)]. The tree is drawn to scale, with branch lengths measured in the relative number of substitutions per site. This analysis involved 28 nucleotide sequences of the inverted repeats. There were a total of 700 positions in the final dataset.

Taking into account the common origin and homology of Ir-b repeats from the *ras85D—CG31344* and *Rlb1—invadolysin* intergenic regions in *D. virilis*, it is possible to estimate the divergence time of these repeats, which has elapsed since the beginning of the accumulation of polymorphism between the two copies of transposons, as well as the time of the divergence between the right and left inverted terminal repeats of one transposon. The divergence of two repeats (Ir-b) of the ancestral copy of the transposon from the *ras85D—Rlb1* intergenic region is shown relative to the repeats (Ir-a) from another copy of transposon corresponding to the *Caf1-55—CG31344*intergenic region, that were taken as outgroup (Figure 5). The timetree was constructed taking into account the calibration estimates of the divergence time of *D. lummei—D. novamexicana* (2.9 million years), the species of the *D. montana* subgroup (4.8 million years), and all the species of the *virilis* group (9–9.5 million years), obtained by Morales-Hojas using multilocus data (Morales-Hojas et al., 2011). It should be noted that the divergence time of Ir-b repeats located close to the *ras85D* and *Rlb1* genes, i.e., the time elapsed from the moment when the transposon had lost its autonomy, is 20 million years. The picture of species relationship, similar to the generally accepted one in the composition and relationship of subphylads, is reproduced using gaps as the fifth base, but taking into account the length of repeats shortened by a quarter in the *Rlb1* region. Such an estimate leads to a revised assessment of the number of substitutions in these repeats and to an inflation of the divergence time (Figure 5). Nevertheless, the result corresponds to the time period when the common ancestor of the *virilis* group species was existed, after the division of the *virilis*—*repleta* clade, which occurred, according to various estimates, from 48 to 24 million years ago (Ross et al., 2003; Adryan and Russell, 2012).

It can be concluded that mobile elements marked by their presence almost all events associated with significant changes in the sequence of the intergenic region located upstream of the *ras85D* gene. When comparing related species, the degradation of areas formed as a result of insertions and rearrangements can be seen. Moreover, the absence of traces of the coding sequences specific for the corresponding transposons superfamilies in the intergenic region sequence of related species indicates the rapid degradation of a significant part of the mobile element sequence.

### Structure and Origin Sources of the *ras85D* Promoter in Different Species

Replacement of the promoter region suggests that in the absence of a new point of assemblage of the preinitiation complex, the gene will be inactivated. In the case of conserved genes with key functions in significant biological processes, the resulting lethal allele will be displaced from the population. The presence of such alleles, fixed in different species, indicates a high rate of restoration of the functional activity of the gene after rearrangement of the promoter region. Let us consider the structure of the promoters and TSSs of the *ras85D* gene and its orthologs in the studied species.

The use of publicly available NCBI databases, such as SRA (for cDNA libraries), EST, Nucleotide, and TSA, nevertheless, leaves open the question of the correspondence of the cDNA data presented in them to the real TSS position. To assess the accuracy of TSS revealing from these data, we compared the results obtained with the TSS analysis data we received using the standard methods for revealing capped transcript ends: cap-trapped expressed sequence tag (EST), cap analysis of gene expression (CAGE), and 5′-end serial analysis of gene expression (SAGE). The results of detailed cross-genomic analysis of TSSs for *D. melanogaster* by the 5′-SAGE method in combination with NGS of the obtained libraries according to the Illumina/Solexa protocols are published in the MachiBase database (Ahsan et al., 2009). The picture of the distribution of reads in the promoter region obtained in this study has shown the need to take into account their frequencies when revealing the reference TSS. Later Hoskins and co-authors, using the methods CAGE, RACE, and RE EST, and Rach and co-authors, using computational methods, have shown that according to the distribution pattern of TSS reads, promoters are differentiated into classes of broad (or weak peaked), narrow peaked, and broad peaked promoters (Hoskins et al., 2011; Rach et al., 2011). The form of TSS distribution is strictly related to the features of expression regulation: narrow peaked promoters determine the expression of luxury genes with spatial and temporal expression restrictions, while broad promoters determine the constant and ubiquitous expression of housekeeping genes (Hendrix et al., 2008; Juven-Gershon and Kadonaga, 2010; Ni et al., 2010; Hoskins et al., 2011).

**FIGURE 6:**
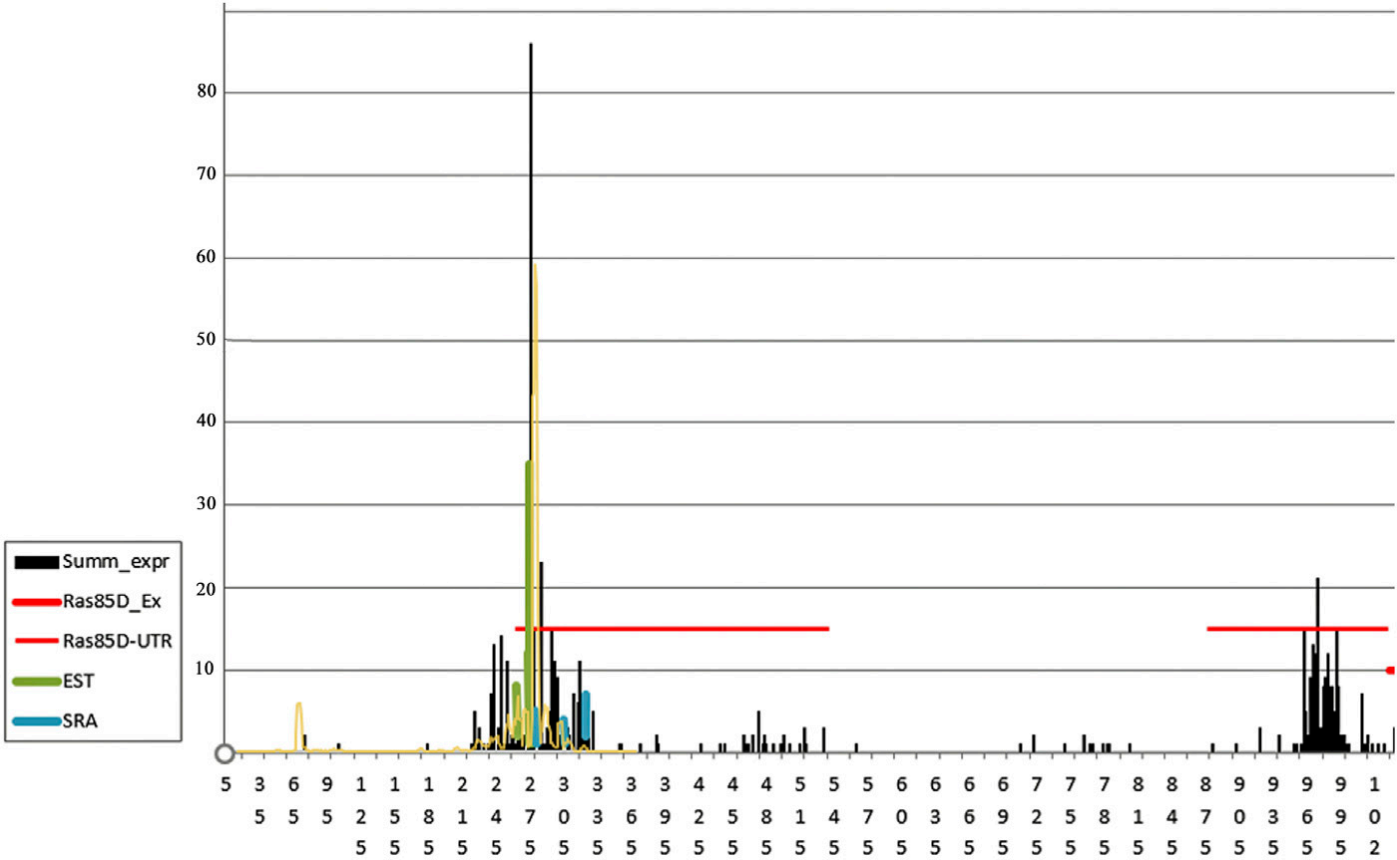
Frequencies of reads around TSS in the intergenic region and 5′UTR of the *ras85D* gene in *D. melanogaster.* Horizontal red lines mark the boundaries of the 5′UTR fragments broken by intron 1. The number of reads is indicated on the Y-axis. The total data on the TSS read frequencies, obtained by the 5′-SAGE method from samples of embryos, larvae, young females, young males, old females, and old males (MachiBase), are highlighted in black; the data obtained from a sample of embryos using the CAGE method are marked in yellow (Hoskins et al., 2011), the reads of the EST and SRA libraries are shown in green and blue **(** Supplementary Table S5 **)**.

The *ras85D* gene is expressed in all cells of an organism throughout its lifetime, but the expression levels of the gene, depending on the tissue type and stage of development, can differ by an order of magnitude or more, according to the SRA data from the public ENA database (European Nucleotide Archive), thus occupying an intermediate position between housekeeping genes and regulated genes. The promoter shape of this gene shows signs of a broad peaked promoter: the reads are densely located in an area of about 100 bp in length and have a pronounced peak in the middle of the area (Figure 6). The figure shows that the SAGE and CAGE data are in good agreement with each other, differing only in the more pronounced TSS peaks for the SAGE data, which is probably caused by an underestimation of the biochemical background formed by degraded and re-capped fragments. The EST data also confirm the noted shape of the promoter, somewhat narrowing the TSS area and bringing it closer to narrow peaked promoters, which is associated with insufficiency of EST data (Hoskins et al., 2011). SRA data do not allow us to determine the shape of the promoter precisely, but mark the area of major gene expression rather accurately.

For most species, only SRA data and the results of predicted estimates of possible TSSs (predicted data) are available, to which, in some cases, data from the TSA and EST libraries are added. The numbers of the SRA and TSA libraries, which were used to perform a BLAST to determine TSSs in *Drosophila* species, as well as the EST data and the accession numbers of predicted mRNA used in the analysis, are presented in Supplementary Table S5. The analysis results do not always coincide, and SRA data in many cases demonstrate the presence of short sequence fragments marked with reads preceding the area of continuous coverage with reads. Although the analysis of the *D. melanogaster* sequence suggests a broad peaked form of the promoter, significant changes in the sequence in other species can lead to both a change in the type of the promoter and the appearance of additional promoters that share in the regulation of the functional activity of the gene (Haberle et al., 2019). The beginning of each fragment of this kind can be considered to be either an erroneous result, or a minor, additional TSS, caused by a random and defective combination of promoter elements. When analyzing the SRA, EST, and TSA libraries, promoter elements were searched in the area of 100 bp from all detected TSSs and in the area of a sharp rise in read frequencies in the promoter neighbourhood. Obviously, such an analysis does not allow the detection of double promoters in the absence of a significant effect of the second promoter, because the most of the data were obtained at one stage of development: imago. However, this analysis allows us to assess the randomness of the coincidence of major and minor TSSs in related species. We considered the following as confirmation of the correct localization of the promoter: coincidence of the TSS localization in the analyzed species according to data from different sources, coincidence with the predicted TSS, and position coincidence in closely related species.

**FIGURE 7:**
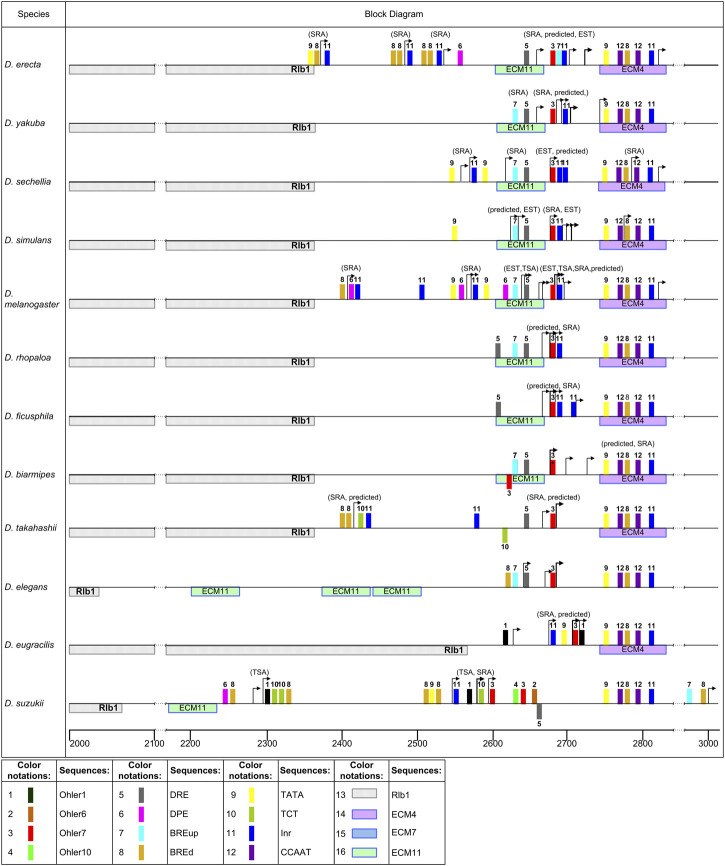
Patterns of promoter elements in the TSS regions of the *ras85D* gene and its orthologs in the studied *Drosophila* species. Distribution of the elements in the promoter region in the *melanogaster* group. The scale below the line shows the position from the start of the overall alignment. Colored rectangles represent promoter elements. The elements shown under the straight line indicate homology with the minus-strand of DNA. TSS are marked with arrows. The gene located upstream is marked on the left side of the diagrams.

**FIGURE 8:**
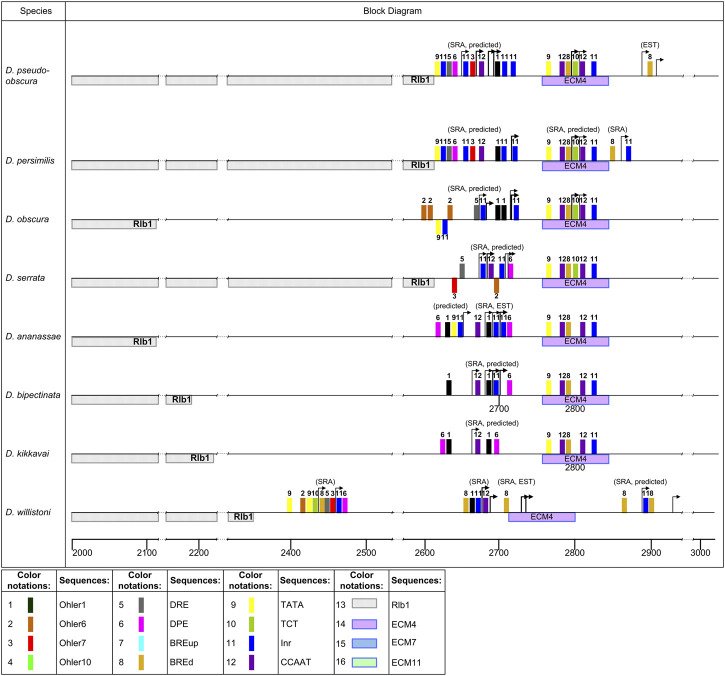
Distribution of the elements in the promoter region in the *obscura, montium, ananassae*, *willistoni* groups. Designations as in Figure 7.

**FIGURE 9:**
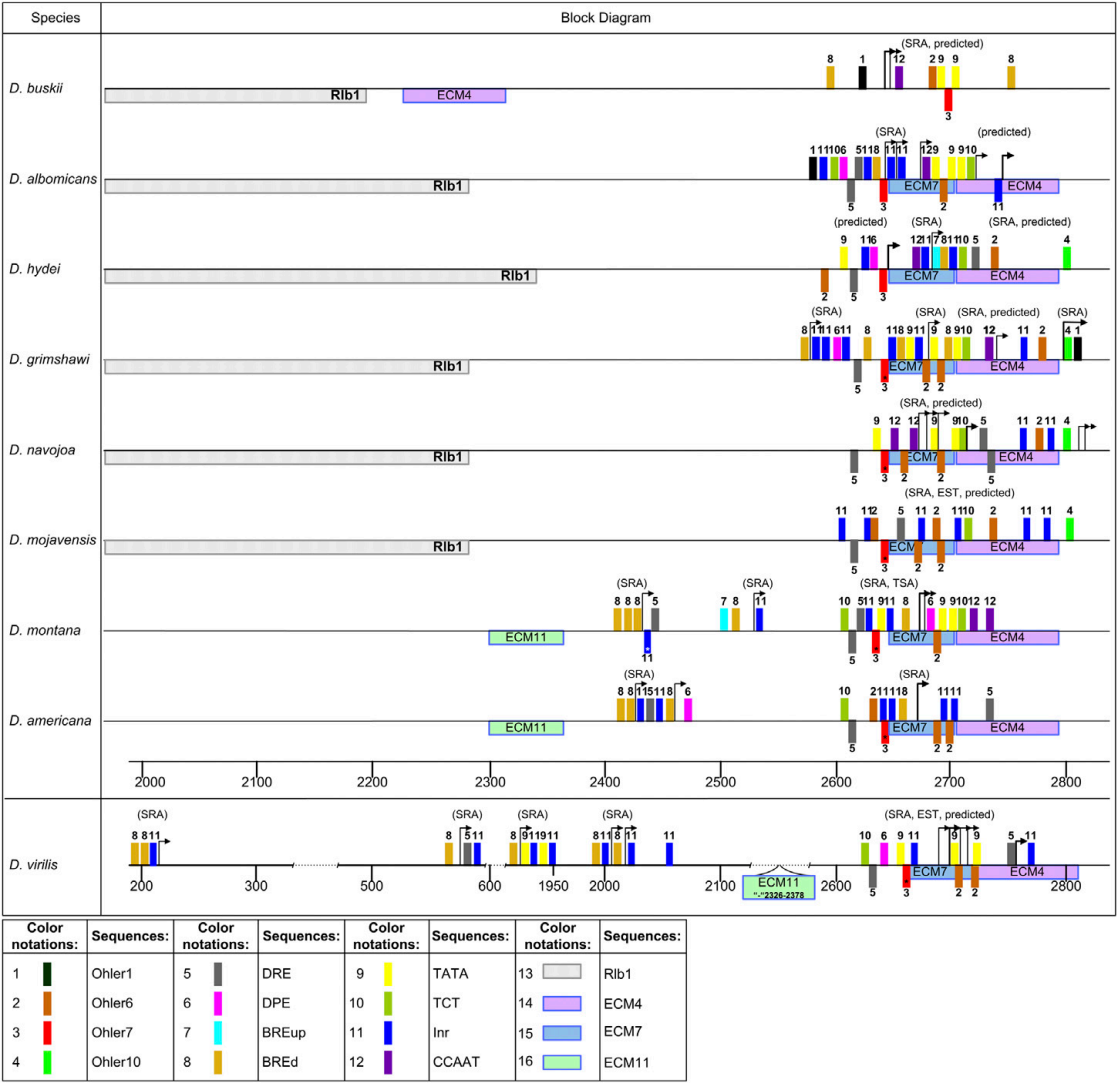
Distribution of the elements in the promoter region in the subgenus *Drosophila*. Designations as in Figure 7.

For information on sequence enrichment with promoter elements in the putative TSS region, see Supplementary Table S6. The distribution of potential promoters in the analyzed species is schematically shown in Figures 7, 8, 9. The positions of the identified promoter elements are shown on aligned sequences, presented in Figure 1. Several typical structures can be distinguished according to pattern similarities in the promoter structures located at the beginning of the area of continuous coverage with reads.

Most of the species belonging to the *melanogaster* group of the subgenus *Sophophora*, with the exception of *D. eugracilis* and *D. suzukii*, show a high homology of the sequence area marked by the beginning of the area of continuous coverage with reads. This area roughly corresponds to the predicted TSS of *ras85D* in *D. melanogaster*, covers an area of 100 bp around the TSS, including ECM11 located upstream, and is closely adjacent to ECM4 downstream (Figure 7, Supplementary Figure S4, the first 10 species). The presence of point mutations, insertions, and deletions does not undermine the conclusion about the general evolutionary origin of this area in these species. A characteristic feature of the putative promoter is the obligatory presence of Ohler7 and DRE elements, with which this area is enriched. The enrichment estimates were obtained both in regard to the intergenic sequence and to randomly generated permutations based on the nucleotide composition of the analyzed sequences using the AME algorithm (McLeay and Bailey, 2010). Such distribution of Ohler7 and DRE elements indicates the effect of selection, which maintains the conserved structure and position of these elements in the promoter region. This region also contains the following elements: BRE down (BRE^d^), Initiator (Inr), and Downstream Promoter Element (DPE). These elements are randomly distributed within the promoter region, and the enrichment of them was statistically insignificant with respect to the intergenic sequence (Supplementary Table S6). Location of reads marking distal TSS positions can vary within 50 bp, but a significant increase in the number of SRA reads, suggesting the highest start frequency, is in most cases associated with the position of the Ohler7 element. Independent transcription starts, located upstream from the main promoter and not connected with it by the area of continuous coverage with reads, were noted for the species *D. erecta*, *D. sechellia*, *D. melanogaster*, *D. takahashii*, and *D. suzukii*. In the area of these TSSs, the elements Inr, TCT, TATA-box, BRE^d^, and DPE may be present, but the canonical arrangement of these elements relative to the transcription start (Lee et al., 2005; Gershenzon et al., 2006; Theisen et al., 2010; Danino et al., 2015; Vo Ngoc et al., 2019) is observed only in some cases. In *D. suzukii*, the element Ohler1 was also found in the area of single starts.

In the species *D. eugracilis* and *D. suzukii* of the *melanogaster* group, insertions of transposones into the intergenic region partially or completely cover the region of the promoter (Figure 7). In *D. eugracilis*, most part of the intergenic region was deleted, and the promoter region remained in the same position relative to ECM4, but lost the DRE and acquired the TATA-box and Ohler1 elements. At the same time, a sharp increase in the number of reads is also associated with the position of the Ohler7 element. The beginning of the area of continuous coverage with reads in *D. suzukii* is shifted upstream on 100 bp from ECM4, in accordance with SRA data. The distribution of TSSs in the range of 50-90 bp in this area suggests that the main promoter in *D. suzukii* belongs to the broad promoter class. It should also be noted that there is a set of TSSs located distally at a distance 250 bp upstream assuming the second promoter. In both cases, the 5′-ends of the distal reads are located to the left of the Ohler1 elements. In the second, most distant promoter, TCT and BRE^d^ elements are also found, while in the main promoter, Inr with TATA and BRE^d^ in the canonical position are additionally observed, as well as Ohler6, Ohler7, and Ohler10 located downstream and DRE positioned on the complementary strand. The predicted TSSs for this species are located deep downstream in the area of continuous coverage with reads.

The region of the putative promoter in seven species of the *obscura*, *montium*, and *ananassae* groups is shifted by about 40 bp from ECM4, in comparison with species of the *melanogaster* group, and has a different composition of promoter elements and a divergent pattern of their reciprocal arrangement (Figure 8). The distribution of distal TSSs in this region also varies within 50–60 bp in the analyzed species. The promoter region in these species is characterized by the presence of CCAAT-box, TATA-box, Inr, DPE, Ohler1, and Ohler7 elements, and has a significant enrichment with the latter two elements. The composition of promoter elements in the species of the *montium* and *ananassae* groups changes insignificantly, and is represented mainly by the elements Ohler1, CCAAT-box, Inr, and DPE. An exception is the species *D. serrata*, in which the deletion of a significant part of the intergenic region reaches the promoter, and is accompanied by the appearance of the Ohler1 element on the minus strand and the DRE element on the plus strand. Degenerate sequences of elements Ohler6 and Ohler7 are noted only on the minus strand. The promoter of species in the *obscura* group has the following characteristic features: in the species *D. pseudoodscura* and *D. persimilis*, the intergenic region is almost entirely deleted; the promoter is on the border with the non-coding sequence of the *Rlb1* gene; the area of Ohler1 elements and downstream Inr elements is shifted to the right; the promoter contains Ohler7 and DRE elements. With an almost complete homology of the promoter sequences of these species, the TSS border of *D. persimilis* is shifted downstream by 70 bp, according to SRA data. The sequence of the *D. obscura* intergenic region was formed with the participation of DNA transposons, and in the left part of the promoter, the fragment from the TATA-box and Inr elements located on the plus strand to the Ohler7 and CCAAT-box elements was replaced by a fragment containing the TATA-box and Inr elements on the minus strand, and several closely spaced elements Ohler6, DRE, and Inr on the plus strand. For all the seven species, no independent transcription starts upstream of the main promoter were found.

The rearrangement of the intergenic region in *D. willistoni* penetrates deep into the intron 1 area, capturing the entire 5′-half of the 5′UTR. At the same time, the TSSs which mark the area of continuous coverage with reads are located only in one area of the sequence of 27 bp in length, which exhibits a considerable homology with the sequences of species of the *montium* and *ananassae* groups corresponding to their characteristic *ras85D* gene promoter. Downstream, this fragment is limited by a region homologous to conserved ECM4, but located on the complementary strand. The promoter of *D. willistoni* is similar to that described for these species in composition of main elements, and includes the elements BRE^d^, Ohler1, Inr, and CCAAT-box (Figure 8). The structure of the element arrangement is similar to the left part of the promoter of these species, where the Ohler1 element is located distally of the Inr and CCAAT-box elements. The area of single starts in *D. willistoni* is located at a distance of 200 bp upstream of the main promoter. It corresponds to the localization of the Ohler7 element, paired with the DRE element, as well as to the canonical position of the TCT and Inr-DPE elements.

Species of the subgenus *Drosophila* exhibit a strong homology and a promoter region pattern unique to these species (Figure 9). It includes ECM7, a fragment of about 40 bp in length located upstream, and a half of ECM4, located downstream on the complementary strand. Distal TSS positions vary within 50 bp and are distributed mainly on the distal, part of the ECM7. The start positions marking the areas of maximum or sharply increasing coverage with reads within the discussed promoter also vary considerably, from the middle of ECM7 to the 3′-end of the inverted ECM4 or even further to the right. In this case, we can talk about the conformity of the promoter shape to a broad promoter, but there is no reason to believe that it has a peak. The composition of the promoter elements is significantly enriched with Ohler6 and Ohler7 motifs, both in terms of their occurrence relative to the rest of the intergenic region and in relation to a randomly generated sample based on the analyzed fragments (Supplementary Table S6). The DRE element does not display a significant enrichment relative to the sequence of the entire inergenic region, but its location at a certain section of the promoter is not accidental. The Ohler7 element in this promoter is always located before the ECM7 sequence on the minus strand, while the DRE elements are located as follows: the first one is placed before the Ohler7 element, and the second is found at the beginning of ECM4. The DRE element is a palindrome, and the sequences showing the greatest homology are present on both strands. Most TSSs show no dependence on the position of the Ohler7 element. Ohler6 is also found predominantly on the minus strand. The promoter is enriched with the core elements INR, DPE, CCAAT-box and BRE^d^, relative to their occurrence in the intergenic region, but their non-random distribution within the promoter sequence itself is not confirmed (Supplementary Table S6). All the analyzed sequences also contain the TATA-box and TCT elements, but their enrichment of the promoter has not been shown. In the species of the groups *repleta*, *grimshawi*, and *immigrans* presented in the analysis, the area of the greatest coverage with reads is shifted downstream and is associated with the elements Ohler6 and Ohler10. Random start sites with the Inr and DRE elements upstream of the promoter are noted for *D. grimshawi* and species of the *virilis* group.

The species *D. busckii* of the subgenus *Dorsilopha* does not have any noticeable homology with the rest of the species in the sequence marked with TSSs. However, the size of the 5′UTR transcripts roughly corresponds to those of the other species, and the elements Ohler1, CCAAT-box, Ohler6, and Ohler7 (on the opposite strand) are present in the start region, as is the TATA-box element (Figure 9). The Ohler1 element preceding the TSS in *D. busckii* is noted in this position in *D. albomicans* and in the species of the groups *ananassae*, *montium*, and *willistoni*.

Thus, the putative region of the main promoter in different *Drosophila* species was formed on the basis of sequences of different origins, which corresponds to the conclusions about the regular capture of the noncoding region of the *ras85D* gene up to intron 1 by rearrangements. We have noted at least six variants of differing promoter structures characteristic of the studied species, which are as follows: the structure noted for the species of the *melanogaster* group, the independent variant in *D. suzukii*, the structure characteristic of the *montium*, *ananassae*, and *obscura* groups, the independent structures in *D. willistoni* and *D. busckii*, as well as the general structure for the species of the subgenus *Drosophila*. Interestingly, *D. willistoni* has a 16 bp section of the promoter region which shows a high homology with the corresponding promoter sequences of the *montium*, *ananassae*, and *obscura* groups, against the background of the surrounding sequence associated with an inversion rearrangement under the probable influence of the *Gypsy* transposon and extending deep into the 5′UTR region. Of interest is also the position of ECM4, which accompanies the main promoter in most species, associated in some cases with a significant increase in expression and located in the species of the subgenus *Drosophila* on the opposite strand as part of the surrounding unrelated sequence.

The composition of the related promoter structures present in each of the three sets of species (Figures 7–9) may change due to accumulated point mutations, insertions or deletions, and rearrangements bordering on the promoter, nevertheless retaining its main features, especially in the right part of the promoter. All the identified structures carry Ohler elements, most often Ohler1, Ohler6, and Ohler7, as the main, constantly present motifs, and the latter two elements are also found on the opposite strand. As noted elsewhere, the DRE element often accompanies Ohler7 (Ohler et al., 2002; Ohler, 2006; Rach et al., 2009). The presence of these elements is typical for broad promoters with low peaks (Rach et al., 2009; Kadonaga, 2012; Danino et al., 2015). There are three main patterns with independent origins in most cases. They are located at a similar distance from the coding sequence and have entirely different structures. Altogether, it implies the possibility of rapid formation (or selection based on the available elements) of an active promoter in an evolutionarily new sequence after the events of insertions of mobile elements or rearrangements.

### The Functional Significance of Evolutionarily Conserved Motifs

According to modENCODE database data obtained using high throughput ChIP-chip and ChIP-seq methods, the *ras85D* gene area of *D. melanogaster* the contains binding sites for more than 40 transcription factors (TFs) and their transcription complex partners from 20 TF superfamilies and four families of coactivator and corepressor proteins (modENCODE Consortium and Roy, 2010; Negre et al., 2011). This set was used as a filter to confirm the specificity of the identified transcription factor binding sites has shown factors. It turned out that only 23 transcription factors were non-randomly distributed over the ECM sequences (Table 3). Transcription factor *lola* and kni binding sites are found most frequently and have been identified in the composition of six and four ECMs, respectively. Enrichment with transcription factor D, disco, Dll, en, Med, pnr, and Ubx binding sites has been detected for three ECMs and enrichment with transcription factor bab1, BEAF-32, sens, Trl, and twi binding sites has been detected for two ECMs.

**TABLE 3:** Enrichment of evolutionarily conserved sequences with transcription factor binding sites (MEME Suite 5.4.1, AME algorithm).

Motif/control	2A/1B	1A/2B	3A/3B/12A	4A/4B/13B	5A/5B	7A/8B/9B	4A/7B	13A/11B
P-val.^1^ ^,^ ^2^ < 0.01; E-val.^1^ ^,^ ^2^ < 0.05	*D, kni, lola, Trl, disco*	*Dll, en, Ubx, bab1, fru*	*Trl, twi, Med*	*BEAF-32, kni, pnr, ttk, Ubx, lola, sens, Dll, en*	*lola, BEAF-32, disco, dl, pnr, kni*	*kni, lola, sc/da,*	*Ubx, bab1, disco, lola, Med, sens, D, Dll, en*	*D, lola, pnr*
P -val.^1^ < 0.01; E -val.^1^ < 0.05	*Med*		*h, lov, ato/da*	*pho*		*twi*		

P, E-val.^1, 2^: the corrected *p*-value of the probability of random enrichment with transcription factor binding sites and the expected number of false positives obtained using two variants of control sequence sets.

1 Obtained by cutting fragments of similar length from the intergenic region, the 5′UTR, and intron 1, except for fragments of the analyzed motif.

2 Generated by random permutations based on the analyzed conserved set of fragments. P, E-val.^1^: indicators p and E, significant only for assessments using the control sequences obtained by method (1). The letter next to the ECM number designates the sequence set that was analyzed. A: Set A (23 species), B: Set B (37 species).

The identified set of TFs has a wide range of functions, from the organization of chromatin and the co-activation and co-repression of transcription complexes, to the control of cell division and cell differentiation processes, cell death and segmentation, as well as the morphogenesis of organs and body systems. The control of the *ras85D* gene expression activity is thus directly related to ontogenetic processes during embryogenesis and the development of the larva and pupa of *Drosophila.* Evolutionary conservatism assumes the support of ECM composition by selection, which determines the set of mandatory regulatory functions. To reveal the specific functions for each of the studied ECMs, we have undertaken a GO enrichment analysis of the biological processes characterizing the sets of specific TFs obtained for these ECMs (Supplementary Table S7).

The biological processes revealed as a result of GO enrichment analysis can be divided into two groups: those that determine the general gene expression activity, and those that depend on the tissue, on the type and state of cells, and on the stage of development. For the analysis, GO processes were ranked according to the adjusted *p*-values; then the first 10 GO-processes associated with tissue and age specificity, as well as all general GO processes falling within the boundaries of the obtained *p*-values were selected. The sets of analyzed genes are represented exclusively by transcription factors and chromatin proteins; therefore, all processes characterizing the formal connection with promoters, DNA, and transcription were removed from the general GO processes. Positive regulation of transcription is characteristic of the TF sets obtained for ECMs 2A/1B and 3A/3B/12A (Table 1), while the ECM1A/2B is not related to an increase or decrease in the basic gene expression. For the remaining ECMs 4A/4B/13B, 5A/5B, 7A/8B/9B, 4A/7B, 13A/11B, both directions of changes in the basic gene expression are shown.

GO processes associated with tissue and cell specificity show functional differences in ECMs. Thus, the effect of the ECM2A/1B sequence on the expression of the *ras85D* gene mediates its participation in the control of cell division, the morphogenesis of salivary glands and tracheal system, retinal innervation and embryo segmentation, as well as cellular responses to BMP and ecdysone stimuli. ECM1A/2B is required to control the development of the leg, wing, ocular, and genital discs and the anteroposterior cell polarization. ECM3A/3B/12A mediate the involvement of the gene in dorso-ventral cell polarization, the development of the nervous system, the differentiation of neurons and photoreceptors, and cuticle formation; ECM5A/5B mediates the participation of the gene in embryonic segmentation and the development of the heart, circulatory system, lymph gland, and peripheral nervous system; ECM7A/8B/9B are needed for the regulation of the mitotic cycle, the formation of neuroblast and stem cell differentiation, the differentiation of chaetes and the development of the peripheral nervous system and Malpighian tubules; ECM4A/4B/13B bring about the role of the gene in the mediated regulation of macromolecule metabolism, the development of genitals, antennae, halteres, wings, and legs, as well as photoreceptor differentiation. ECM4A/7B is similar in functional activity to ECM3A/3B/12A and ECM4A/4B/13B, while ECM13A/11B is similar to ECM5A/5B.

It can be noted that ECM7A and ECM13A, both in terms of the probable composition of transcription factor binding sites and in regard to the participation in GO processes, largely duplicate the regulatory functions of the other motifs that are mandatory in the non-coding sequences of the *ras85D* gene and retain their order and position on the strand. ECM4 differs in that it possesses a set of unique regulatory functions for the given cluster of conserved motifs, accompanies the downstream promoter region in most species, retains its localization in relation to the mandatory motifs, but may be found in an inverted position.

## Discussion

The presented picture of the divergence of the structure of the intergenic region and 5′UTR of the *ras85D* gene in *Drosophila* suggests several important evolutionary conclusions. They concern the rate and possible mechanisms of restoration of the functional activity of the gene after replacement of the promoter and a significant part of the regulatory sequences, as well as the localization of the regulatory sequences of the *ras85D* gene in the intergenic region and, to a greater extent, 5′UTR.

The first and most unexpected conclusion is the ability of the gene to quickly and completely change a significant part of the regulatory sequence, including the promoter region. The surprise of this conclusion lies in the fact that random insertions and rearrangements that change the promoter region of the gene inevitably disrupt its normal expression. *D. melanogaster* lines carrying various *ras85D* mutations in the promoter region (Bellen et al., 1989; Parks et al., 2004 (in Supplementary Table S2)) exist either in a heterozygous state, maintaining a lethal allele on the balancer, or have morphological and physiological abnormalities incompatible with effective survival and reproduction in nature. In this case the time for the recovery of normal or at least minimally sufficient expression of the mutant allele is extremely limited: such alleles are present in the population with a low frequency, and the probability of their complete displacement is inversely proportional to the frequency. The restoration of the functional activity of a gene should occur almost instantly on the scale of evolutionary time, within several tens of generations.

Nevertheless, in the sequences of different species, we see various combinations of fragments of independent origin, which testify to the successful reorganization of the regulatory region, its fixation in the population, and further inheritance by descendant species. Independent rearrangements of the sequence, which covered the non-coding region of the gene up to intron 1, occurred three times: in the common ancestor of the subgenus *Drosophila*, in the subgenus *Sophophora* during the divergence of the ancestral species of the *melanogaster* group from the other groups, and during the later inversion rearrangement in *D. willistoni*. In most cases, such a “new” sequence, which means a sequence of independent origin, contains a promoter which has its own characteristic pattern of elements, distinct in the composition and sequence of element distribution in the corresponding region of the sequence. The similarity of promoter structures of a common origin can be seen primarily from the key elements of a promoter of the broad type, such as Ohler and DRE, as well as CAAT-box and Inr elements. It cannot be argued that all these elements are necessary for the effective functioning of these promoters, but the considerable similarity of patterns assumes the support of each of these variants by selection. A possible scenario for the formation of these patterns, taking into account the short period of restoration of gene expression activity, is the selection of promoter elements from suitable motifs located at an optimal distance to the preserved downstream regulatory sequences and the coding part of the gene.

Subsequent insertions of mobile elements and the accompanying rearrangements of the intergenic region left only the basal fragments of sequences from earlier rearrangements, as, for example, in species of the subgenus *Drosophila*, or “broke up” the ancestral sequences, displacing their fragments distally (as in the species *D. elegans*, *D. suzukii*, and *D. eugracilis* from the *melanogaster* group), or else led to deletions of most of the intergenic region. Such modifications are causing a change in the position of the promoter. For example, in *D. suzukii*, the promoter shifted upstream following a similar displacement of the homology region, while in *D. eugracilis* and *D. persimilis*, the promoter moved downstream. Such events are accompanied by a complete or partial change in the composition of the promoter elements, which again suggests the possibility of a rapid selection of a new optimal combination of elements, within the boundaries of an effective control of gene expression. This assumption is supported by the presence of individual TSSs in the prepromoter region of many species, localized close to the motifs with a high homology with promoter elements, but probably not playing a significant role in the regulation of gene expression. The conclusion about the lack of functional significance of short transcripts from additional TSSs was also made by Hoskins and co-authors, based on the results of analyzing the transcriptome of *D. melanogaster* embryos by the CAGE method (Hoskins et al., 2011). Obviously, an essential condition for choosing a promoter that performs basic gene expression regulation is the accessibility of enhancers to promoter elements, which ensures a complete assembly of the transcription complex (Ohler and Wassarman, 2010; Ibrahim et al., 2018). In this case, the structure of the sequence, including the distribution of elements such as nucleosome depleted regions, insulators, etc., will provide the choice of a promoter and determine the efficiency of expression from different regions of the sequence carrying a particular set of promoter elements. At the same time, the possibility of selecting a suitable composition of promoter elements suggests a wide range of possible variants of the distribution of these elements in the promoter region, with the possibility of their synergetic interactions and an efficient assembly of the transcription complex, which was noted in a number of studies earlier (Dikstein, 2011; Mwangi et al., 2015).

The correspondence between rearrangements of the non-coding region of the gene and the conserved motifs and fragments homologous to the substituted sequence located within them, allows us to conclude that there are some mechanisms for restoring the expression activity of the *ras85D* gene. The appearance of such motifs assumes their direct copying from the replaced sequence. Two examples among the studied regulatory sequence structures directly indicate such a mechanism. One example is the presence of an inverted ECM4 immediately behind the promoter region in the species of the subgenus *Drosophila*: the position of this motif corresponds to the direct ECM4 in all the other species, but is found on the opposite strand in the sequence having an independent evolutionary origin. Another example is the homology of the promoter region in *D. willistoni* to the corresponding region of species of the *ananassae*, *montium*, and *obscura* groups. In the latter case, we are faced with a phylogenetic discordance caused by the presence of an inverted ECM4 in *D. willistoni*, as in the species of the subgenus *Drosophila.* This discordance may be caused by a false positive homology of the compared motifs. It is supported by the least significant E-values of the general ECM pattern in this species (Figure 1), the strong degeneracy of ECM4 itself (p-val. = 7.25e-5 compared to 7.48e-26 <p-val. <1.60 e-13 in other species), and the possibility of alignment of its right side with respect to the downstream sequences in species of the subgenus *Sophophora*. The homology of this motif in species of the subgenus *Drosophila* is highly significant (p-val. <1.60e-13); in the genomes of species of the subgenus *Drosophila*, there are no homologous motifs located outside the analyzed locus (according to BLAT Search Results Genome Browser UCSC). In the *D. melanogaster* genome, this motif is reproduced in intron 1 of the *ine* gene (UCSC Genome Browser, chr2L: 4,475,427-4,475,476) and in the *Nep4—Cpr92F* intergenic region, closer to the *Nep4* promoter (UCSC Genome Browser, chr3R: 20,660,789-20,660,838). *ine* and *Nep4* are involved in the formation of the nervous system, genitals, and excretory system, as well as muscle cell differentiation. The functional activity of these genes largely coincides with that revealed for ECM4, which confirms the Gene Ontology of biological processes indicated for it. An accidental presence of this motif in the composition of the replacement sequence seems unlikely.

The possibility of copying a fragment of the ancestor, or the sequence replaced as a result of a rearrangement, can be explained by the conversion mechanisms of sequence restoration: these mechanisms are activated during the rearrangement process. Being one of the two mechanisms of homologous recombination, the canonical mechanism of meiotic conversion occurs at the sites of double-strand breaks in the chromosome and is associated with the formation of two Holliday junctions (Chen et al., 2007; Do et al., 2014). Conversional replacements of homologous and non-homologous sequences are widespread in *Drosophila* genomes (Chen et al., 2007; Johnson-Schlitz et al., 2007; Wei and Rong, 2007; Kane et al., 2012; Velandia-Huerto et al., 2016), and a significant part of them are associated with double-strand breaks induced by the mobile elements transpositions (Lankenau, 1995; Hedges and Deininger, 2007; Robert et al., 2008; Kane et al., 2012). In the above examples, motifs homologous to ancestral ones, but surrounded by a new sequence, are located at the 3′-end of the rearrangement region, close to the point of the putative transposon insertion. A possible scenario for the formation and maintenance of such variants is associated with “successful” conversional replacement of fragments of an evolutionarily new sequence located in the place of functionally significant regions, such as enhancer blocks and promoter regions. Selection will maintain such sequences, which partially or completely restore the transcriptional activity of the gene, and eliminate lethal and partially lethal alleles.

The third conclusion is related to the analysis of the distribution of evolutionarily conserved motifs in the analyzed sequence in different species. A strict demarcation line turns out to be the boundary of intron 1, beyond which the rearrangements that capture the intergenic region do not extend. A stable pattern of this fragment is formed by ECM1, ECM2, ECM3, and ECM5, and its variation is mainly associated with insertion-deletion polymorphism and with the multiplication of dinucleotide microsatellite repeats and poly-T tracks. A certain conservatism also characterizes the area from the promoter region to intron 1, but in this case the region includes ECM4, which has signs of a conversion transfer to a younger sequence in species of the subgenus *Drosophila*. This observation does not exclude the presence of enhancers involved in the regulation of *ras85D* gene expression in the intergenic region, but suggests that regulatory sequences that are critical for the functional activity of this gene are located downstream of the promoter.

The *ras85D* gene is conserved, its functions are just as conserved and important for the control of ontogenetic processes and the maintenance of normal vital activity of cells, tissues, and the whole organism. The presented picture of the evolution of the non-coding sequence and in particular the prepromoter region of this gene allows us to conclude that each evolutionary lineage has undergone at least one event of replacement of a significant part of this area. It seems that the picture of the distribution of regulatory sequences is not accidental. Perhaps the answers to the questions of how common the observed distribution of conserved and variable regions and regulatory motifs is for other conserved genes, and in which taxonomic groups of living organisms, will bring us closer to understanding to what extent and in what ways the formation of the structure of regulatory sequences can be controlled.

Finally, let us note one more unexpected conclusion. Based on general ideas about the formation of adaptation mechanisms and isolating barriers in the course of evolution, conserved genes should maintain the homeostasis of an organism and serve as the foundation on which all evolutionary innovations are built. However, the results obtained suggest that periodic (but not critical) changes in the regulatory activity of a gene, during the evolution of its non-coding sequence, can have their effect on the ontogenetic process, also leading to evolutionarily significant changes.

Thus, we can conclude:

1) High variation of the prepromoter region and distal part of the 5′UTR is associated with rearrangements caused by insertions of mobile elements. These parts of the sequence are of independent evolutionary origin.
2) Motifs (ECMs) that are critical for the regulation of the processes of development and functioning of cells are located downstream of the promoter and, along with the promoter region, can be transferred to evolutionarily new sequences using the mechanisms of genetic conversion.
3) Evolutionary changes in the regulatory region of a conserved gene, leading in most cases to its inactivation or abnormalities incompatible with normal development, can be restored to an acceptable level in the shortest possible time, practically instantly on the evolutionary time scale.

## Data Availability

The article is based on the analysis of publicly available data. All datasets presented in this study are included in the article/ Supplementary Material.
