# Supplementary material for: Episodes of Rapid Recovery of the Functional Activity of the ras85D Gene in the Evolutionary History of Phylogenetically Distant Drosophila Species
Source: Front Genet. 2022 Jan 12;12:807234. doi: 10.3389/fgene.2021.807234 (PMC8790561; doi:10.3389/fgene.2021.807234)
Supplement: Supplementary file 1 [file DataSheet2.docx]

**Contribution to the Field Statement**

The C-enigma of David Comings and Susumu Ohno testifies to the enormous redundancy of the genome, in which islets of coding sequences are immersed in a sea of non-coding DNA. Noncoding DNA contains many functionally active sequences, from mobile elements and various noncoding RNAs to structurally and regulatorily significant sequences. The implementation of genetic information depends on the coordinated action of most of these sequence elements. It can be expected that the functional activity of genes, and especially of conservative housekeeping genes, is supported by a stable pattern of regulatory elements. This pattern should be inherited by descendant species from a common ancestor and gradually changed during evolution. But this is not the case. The variability of non-coding DNA sequences is an interesting evolutionary puzzle demonstrating dramatic changes in the promoter region and upstream intergenic spacer even in such conservative genes as the *ras85D* of *Drosophila*. As a molecular switch, the protein encoded by this gene retains the structure of domains and functional sites throughout the evolutionary history of **metazoa**. Its molecular and biological functions remain unchanged in different *Drosophila* species. At the same time, the variability of the promoter region, intergenic spacer, and a significant part of the 5'UTR indicate an independent evolutionary origin of these sequences. The current study is intended to answer the questions of how often and in what manner these regions were replaced? How the functional activity of the gene could be preserved and maintained?
