# Supplementary material for: Episodes of Rapid Recovery of the Functional Activity of the ras85D Gene in the Evolutionary History of Phylogenetically Distant Drosophila Species
Source: Front Genet. 2022 Jan 12;12:807234. doi: 10.3389/fgene.2021.807234 (PMC8790561; doi:10.3389/fgene.2021.807234)
Supplement: Supplementary file 2 [file DataSheet1.docx]

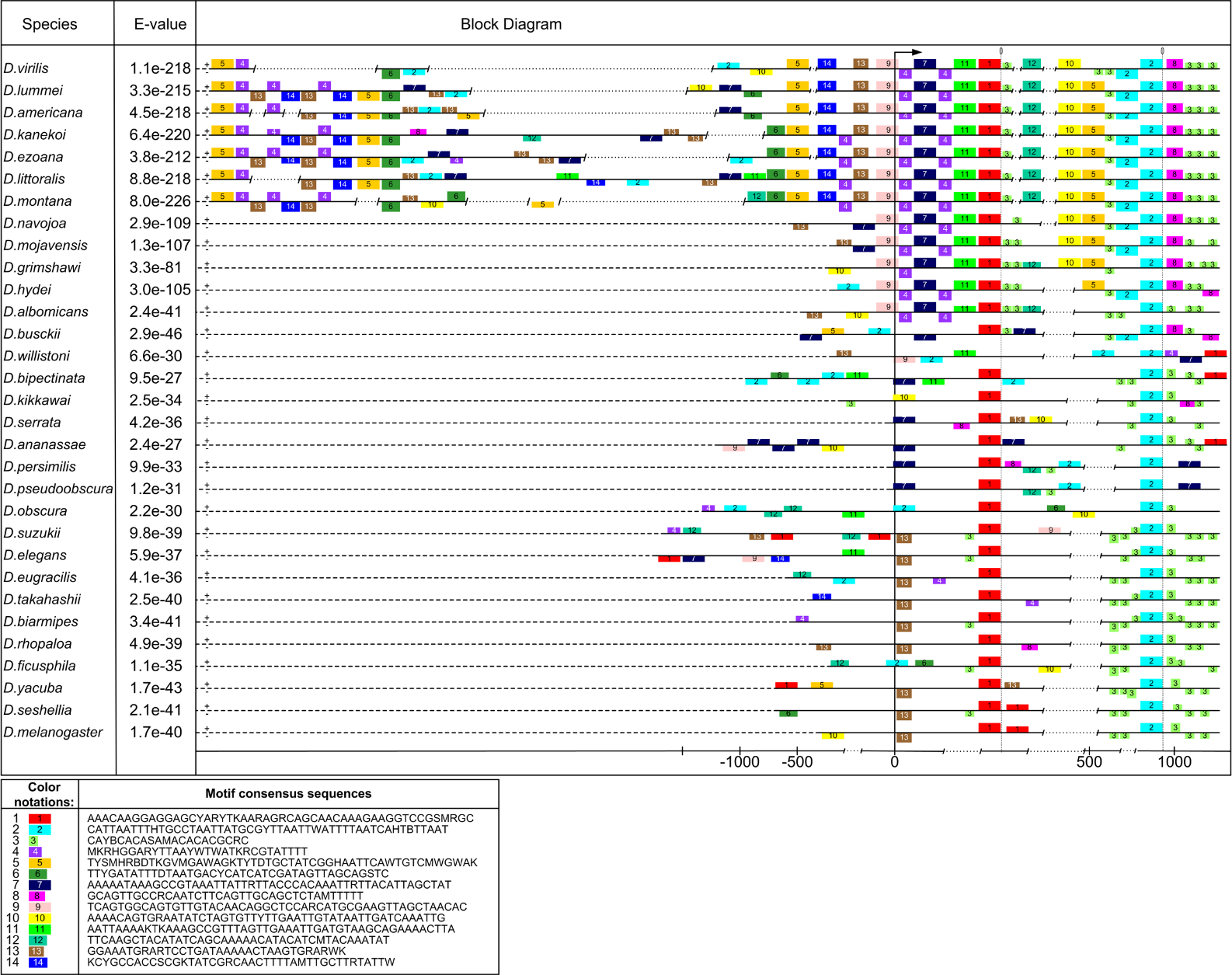


**Supplementary Figure S1. Variability of the intergenic spacer, promoter and 5'UTR region of the *ras85D* gene of *Drosophila* species of the different degrees of relationship.** Scheme of distribution of the evolutionarily conserved sequences (ECMs) and insertion-deletion polymorphism. The structure of the ECMs obtained using the MEME algorithm. The boxes above and below the line show the location of the sequences on the plus-strand and on the minus-strand respectively. The reduced box height marks degenerate ECMs. Dotted lines denote deletions. The structure of the region of two closely related species of the *melanogaster* group (*D. simulans, D. erecta*), one species from the *obscura* group (*D. persimilis*) and four species of the *virilis* group (*D. novamexicana, D. lacicola, D. flavomontana, D. borealis*) are not shown due to the structural similarity. Structural homology is shown for the following options: *D. melanogaster* = *D. simulans, D. yakuba = D. erecta, D. pseudoobscura = D. persimilis, D. americana = D. novamexicana, D. montana = D. lacicola = D. flavomontana = D. borealis.*


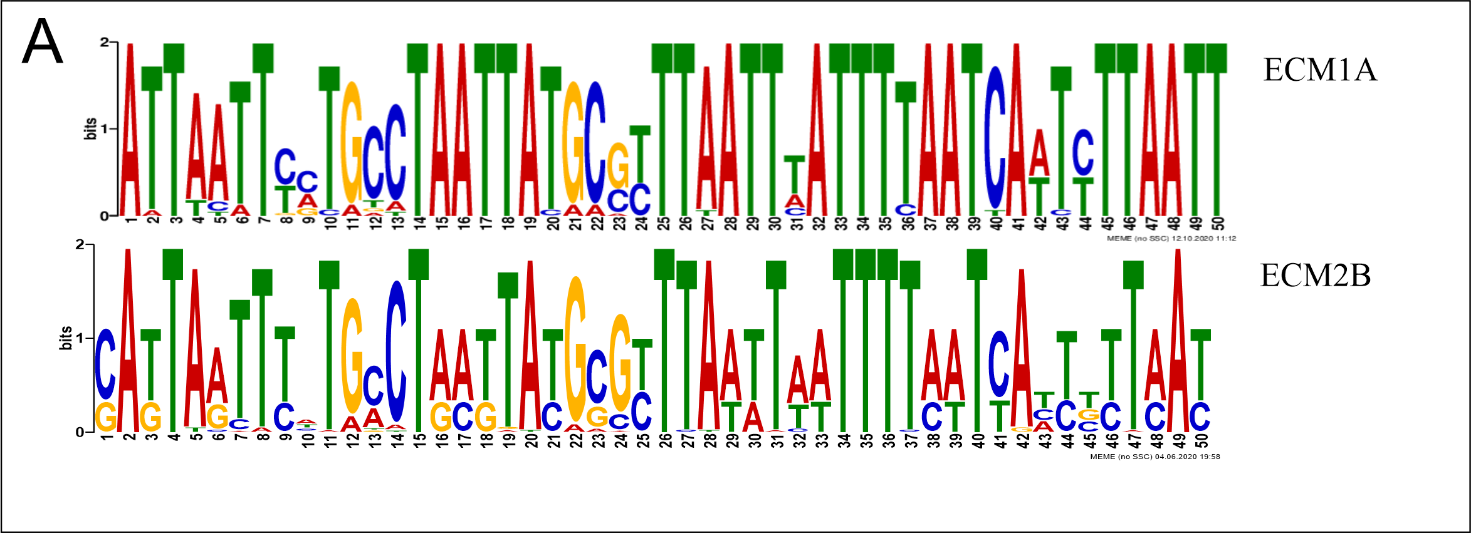


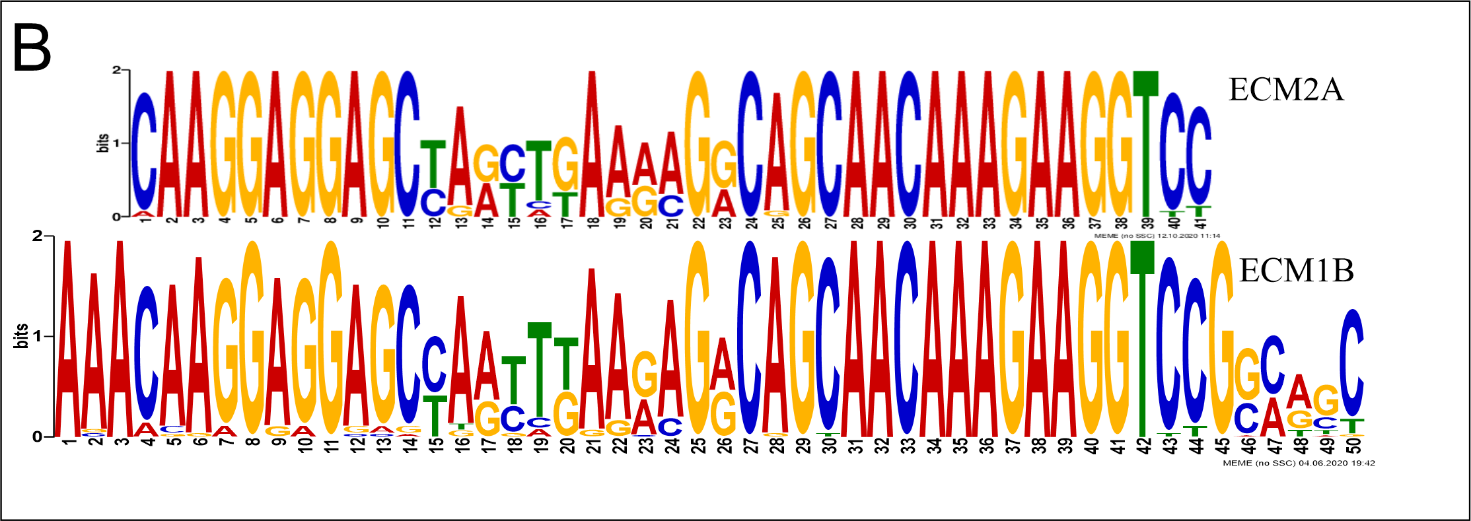


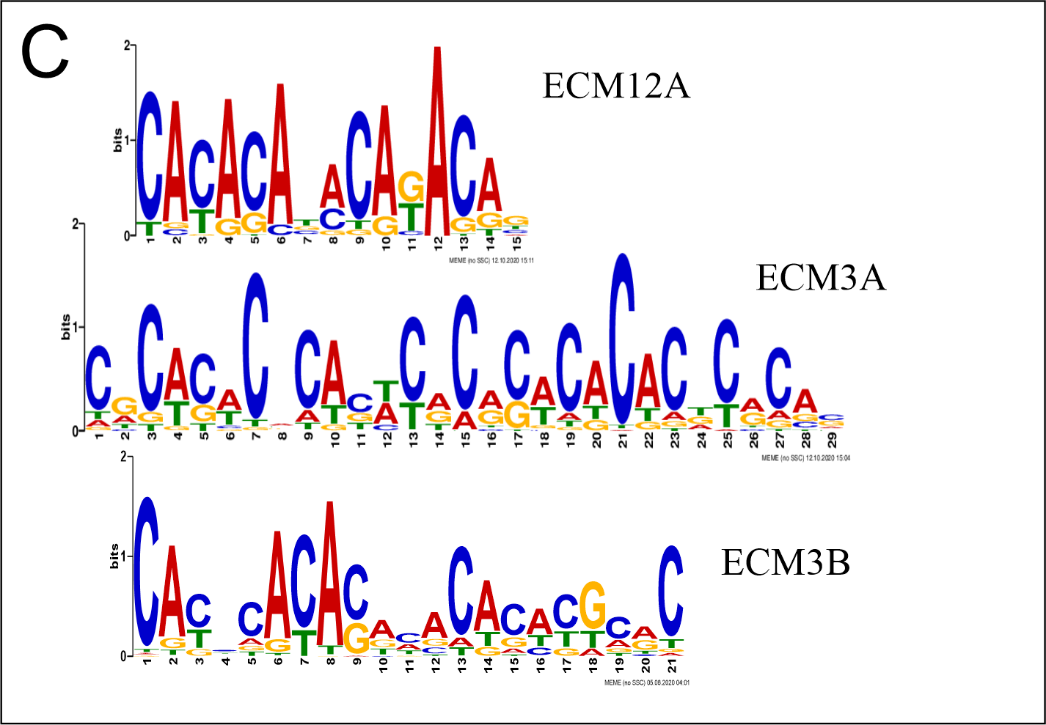


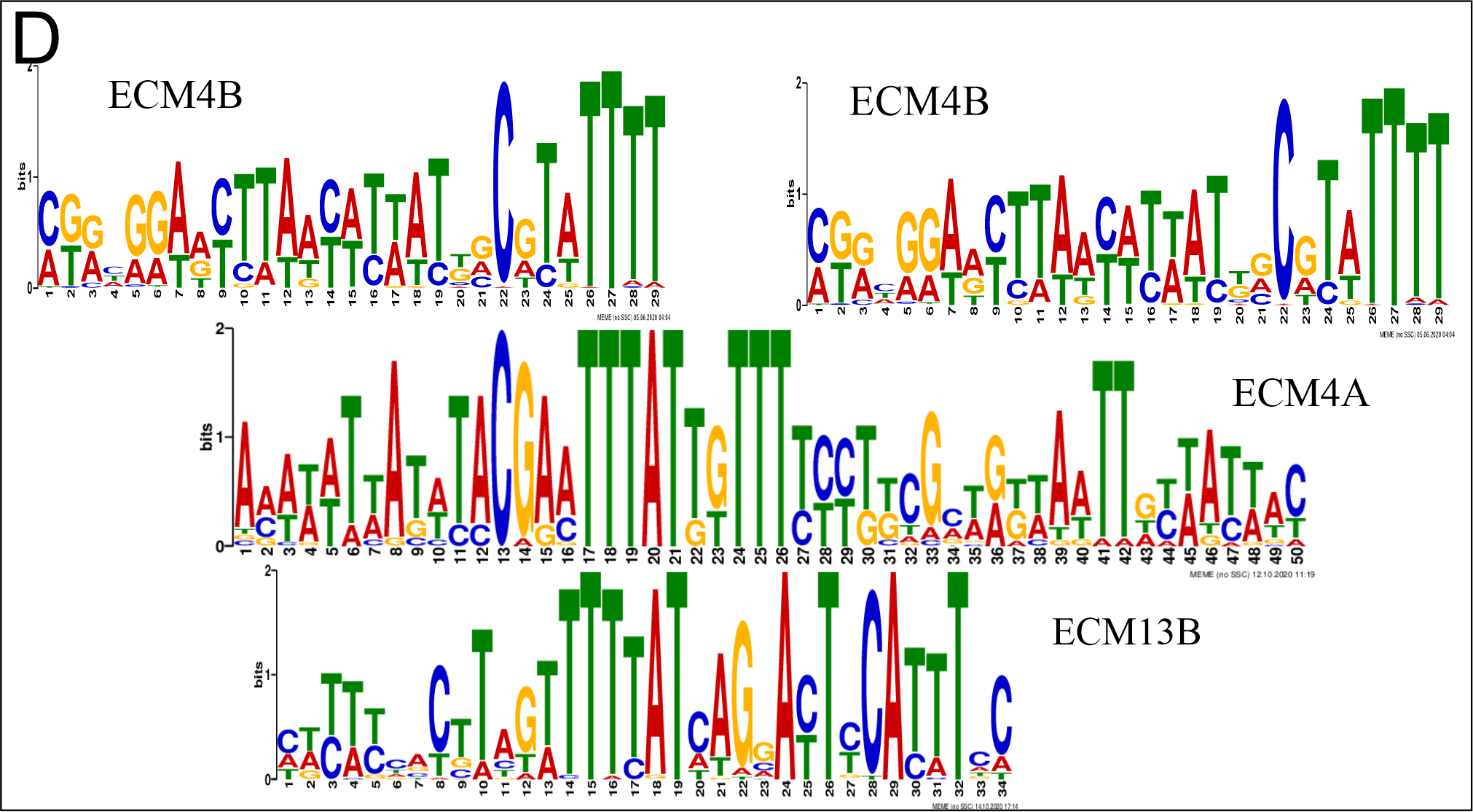


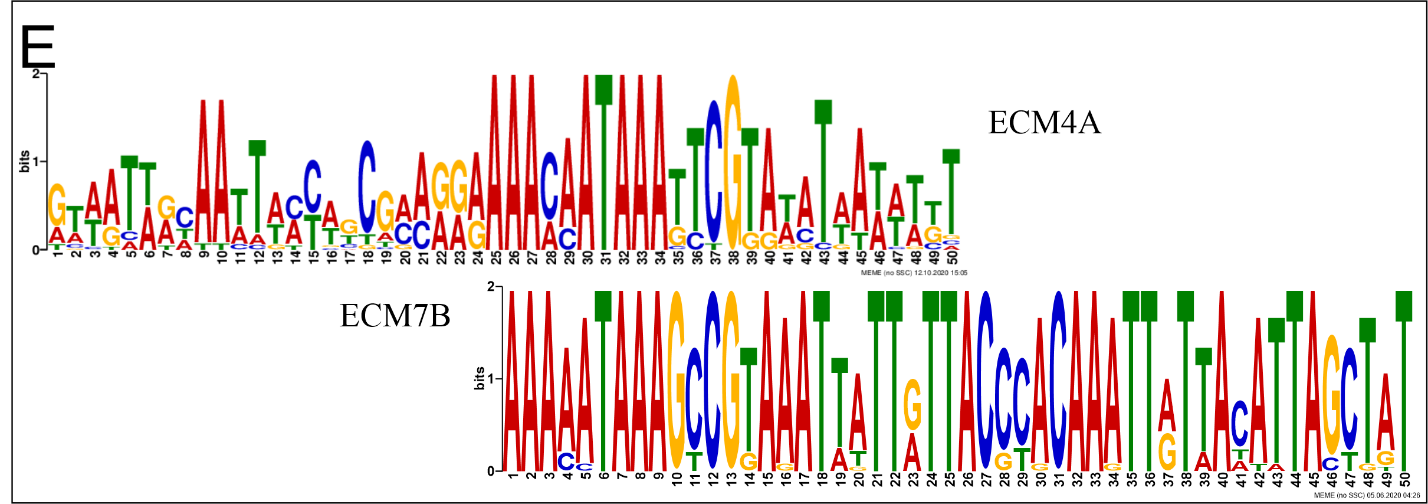


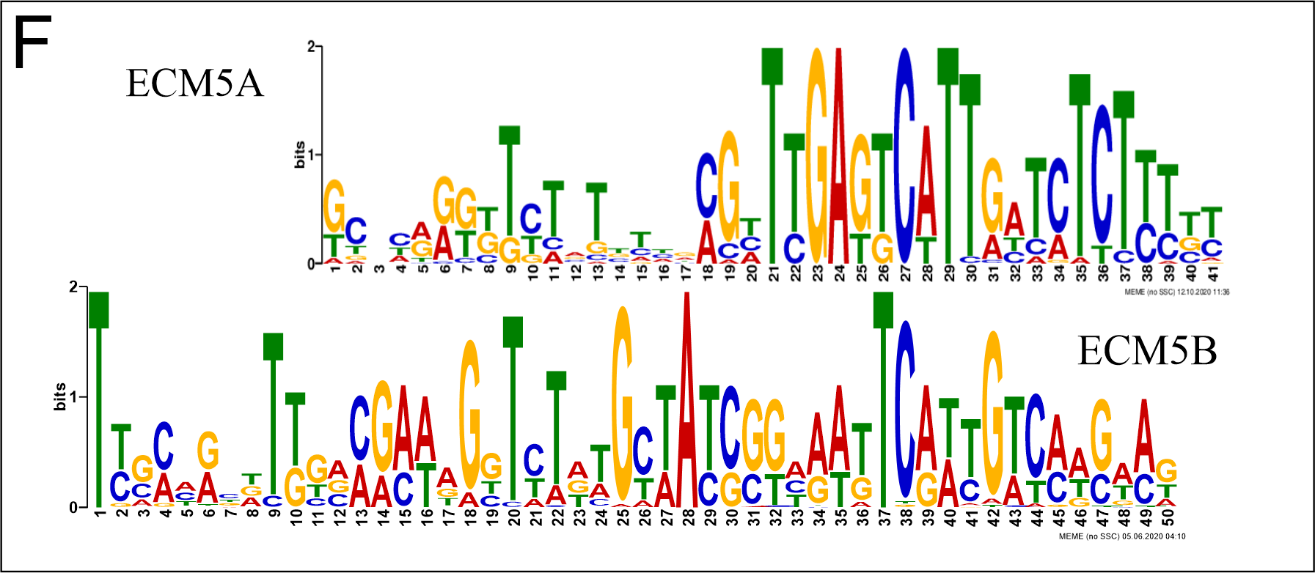


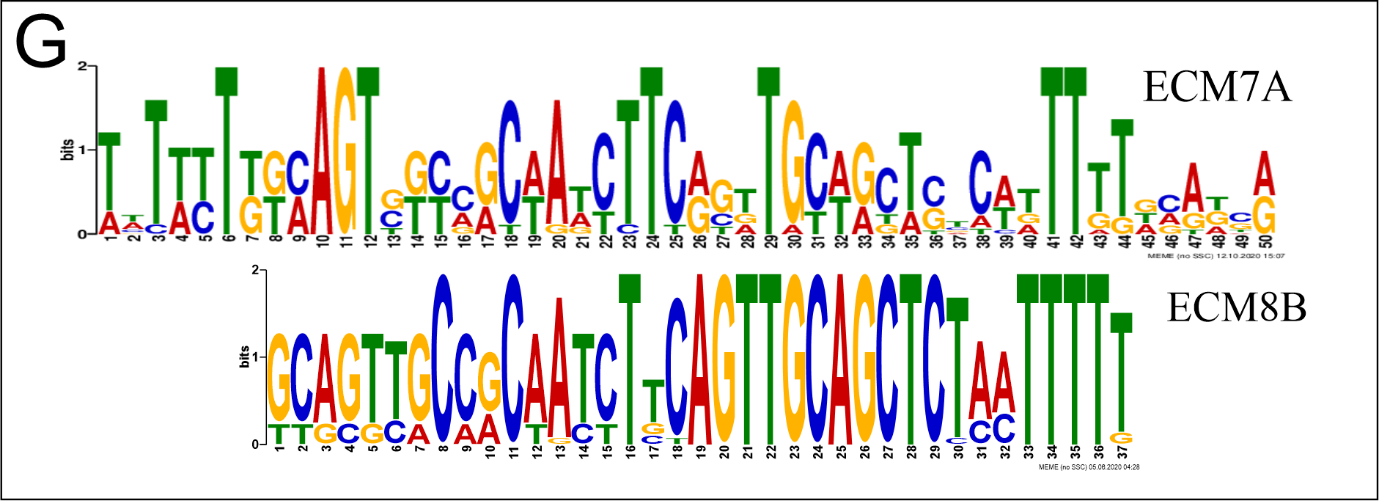


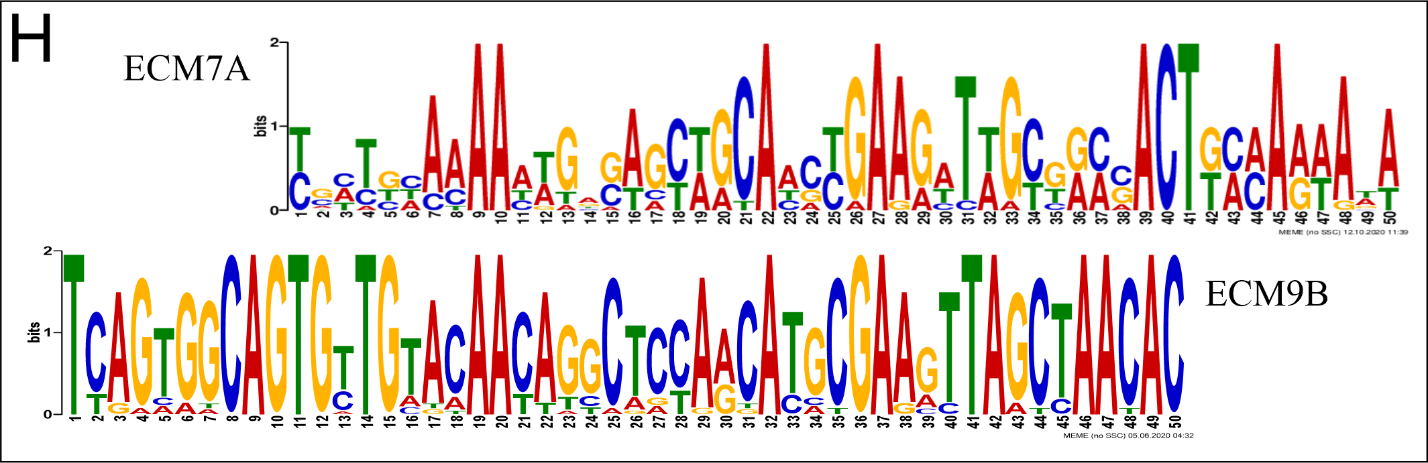


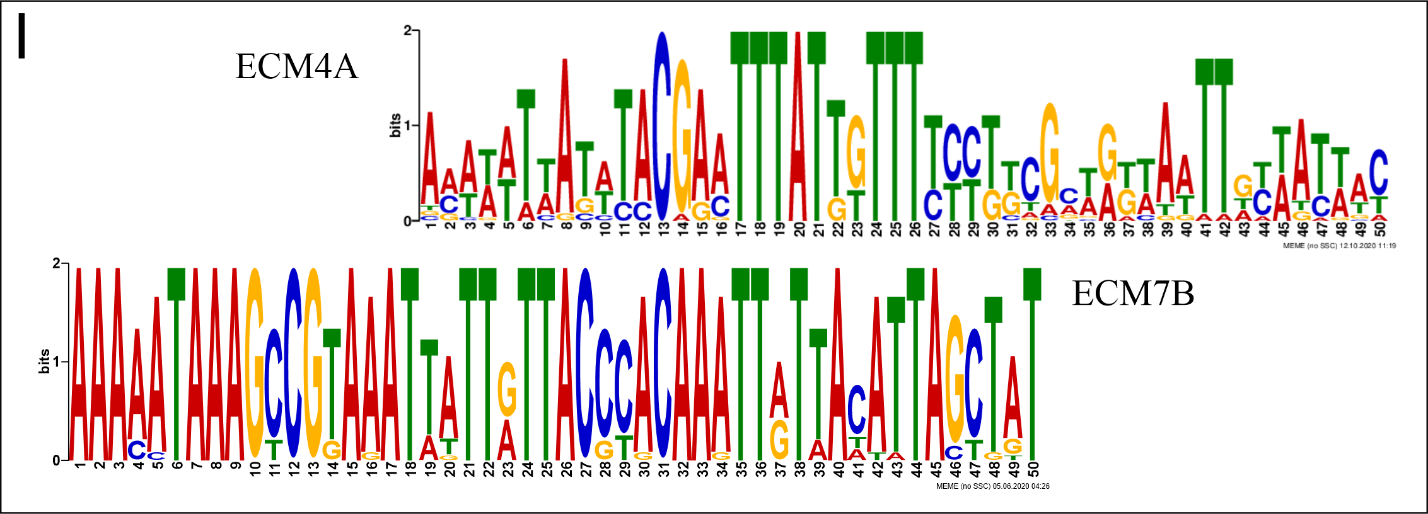


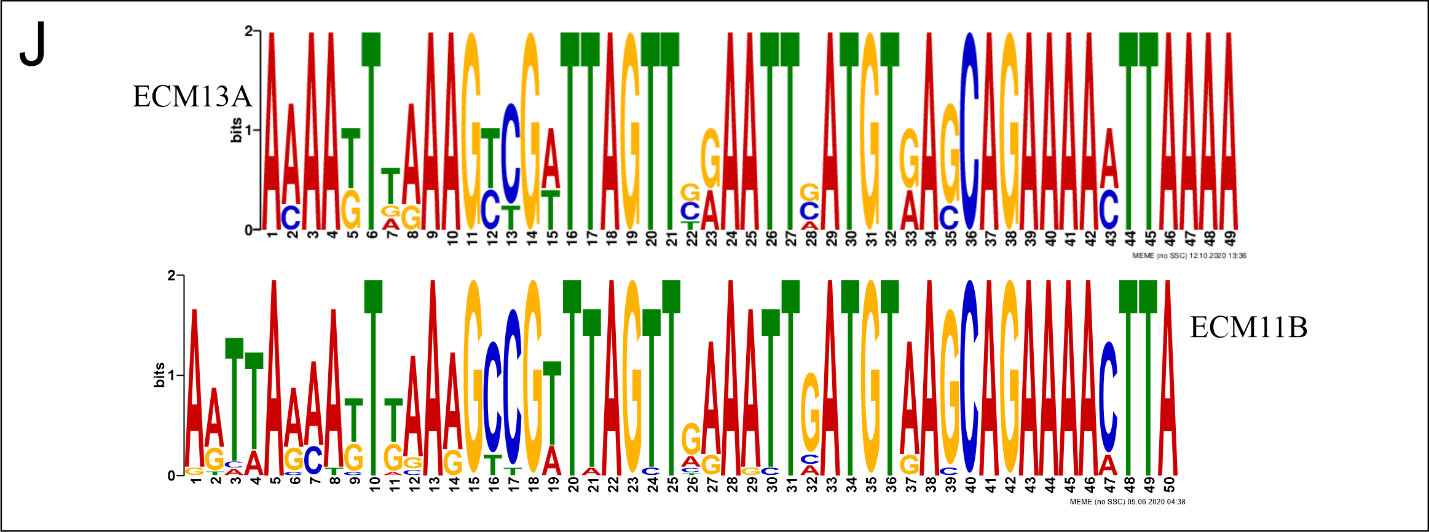


**Supplementary Figure S2. Sequence logos of ECMs.** Structural homology of the ECMs pairs that were obtained on the base of comparison of two sets of sequences: Set A includes 23 species from different groups of *Drosophila*, excluding closely related species that demonstrate high homology of noncoding regions; Set B includes all 37 species. (A) ECM2A : ECM1B; (B) ECM1A : ECM2B; (C) ECM12A : ECM3B : ECM3B; (D) ECM4A : ECM4B : ECM13B; (E) : ECM5A : ECM5B; (F) ECM7A : ECM8B; (G) ECM7A : ECM9B; (H) ECM4A : ECM7B; (I) ECM4A : ECM7B; (J) ECM13A : ECM11B.





**Supplementary Figure S3. Chromosomal rearrangements leading to differences in the position of the *ras85D* gene on the chromosome and in its gene environment in *D. virilis and D. melanogaster*.** Arrows point to the replacement of the genetic environment of the *ras85D* gene in the *D. virilis* genome relative to *D. melanogaster.* The scales indicate positions of the loci on the *D. melanogaster* chromosome 3R, and the scaffold 13047 of *D. virilis*.

| Species | Homol. bound.  1* | | Homol. bound.  2* | | *Transposon Name** | Source | Transposon class | Similarity | Dir | Score | Gene Region | Homology in related Sp. |
| --- | --- | --- | --- | --- | --- | --- | --- | --- | --- | --- | --- | --- |
| *D. willistoni* | 145 | 206 | 2409 | 2470 | *Homo5_hAT* | DMo | DNA Tr. | 0.7377 | c | 108* | Intergenic Sp. | - |
|  | 333 | 431 | 2601 | 2799 | *Gypsy1* | SM | LTR Retrotr. | 0.7742 | c | 247 | Pr | - |
| *D. pseudo-obscura* | 567 | 649 | 3602 | 3684 | *L2-10* | Hmel | Non-LTR R. | 0.7901 | d | 280 | Intron1 | Gr.*obscura* |
|  | 580 | 648 | 3615 | 3683 | *P-1* | DBp | DNA Tr. | 0.7941 | c | 215 | Intron1 | Gr.*obscura* |
| *D. persimilis* | 538 | 620 | 3573 | 3684 | *L2-10* | Hmel | Non-LTR R. | 0.7901 | d | 280 | Intron1 | Gr*.obscura* |
| *D. obscura* | 63 | 46 | 2022 | 2040 | *P-3* | AP | DNA Tr. | 0.7561 | с | 11 | Intergenic Sp. | *-* |
|  | 395 | 515 | 2372 | 2492 | *Helitron-N1* | DAzt | DNA Tr. | 0.7686 | c | 598 | Intergenic Sp. | *-* |
|  | 379 | 517 | 2356 | 2494 | *HelitronN-1* | DPe | DNA Tr. | 0.7482 | c | 594 | Intergenic Sp. | *-* |
|  | 537 | 588 | 2514 | 2565 | *Inverted repeat* (YASS) | - | - | E_val_ = 0.0861 | c-d | 74 | Intergenic Sp. | *-* |
|  | 1 | 51 | 1978 | 2028 |  |  |  |  |  |  |  |  |
| *D. suzukii* | 127 | 190 | 2137 | 2200 | *Homo10 hAT* | DMo | DNA Tr. | 0.6563 | c | 95* | Intergenic Sp. | *-* |
|  | 377 | 475 | 2387 | 2485 | *Gypsy-5* | DTa | LTR Retrotr | 0.6300 | c | 95* | Intergenic Sp. | *-* |
|  | 516 | 584 | 2526 | 2594 | *Gypsy-15* | DRh | LTR Retrotr | 0.6338 | c | 91* | Intergenic Sp. | *-* |
|  | 872 | 911 | 2985 | 3026 | *Gypsy-23* | PBa | LTR Retrotr. | 0.8049 | d | 221 | Intron1 | S/gen. *Sophophora* |
| *D. takahashii* | 188 | 270 | 2125 | 2639 | *Hoana5* | DAna | DNA Tr./hAT | 0.7907 | c | 367 | Intergenic Sp.-Promoter | gr. *melanogaster* |
| *D. eugracilis* | 27 | 105 | 2524 | 2602 | *INVADER3_I_Gypsy* | DMe | LTR Retrotr | 0.6076 | c | 100* | Intergenic Sp. | *-* |
|  | 54 | 104 | 2551 | 2601 | *Gypsy-11* | DRh | LTR Retrotr | 0.6471 | c | 95* | Intergenic Sp. | *-* |
| *D. elegans* | 90 | 182 | 1990 | 2125 | *Gypsy-1* | DBi | LTR Retrotr. | 0.6277 | с | 91* | Intergenic Sp. | *-* |
|  | 224 | 280 | 2167 | 2247 | *P-1N1* | DBp | DNA Tr. | 0.7018 | d | 95* | Intergenic Sp. | *-* |
|  | 439 | 538 | 2406 | 2505 | *piggyBac-N1* | DWil | DNA Tr. | 0.6408 | c | 95* | Intergenic Sp. | *-* |
|  | 557 | 647 | 2524 | 2617 | *hAT-1* | DRh | DNA Tr. | 0.7978 | c | 398 | Intergenic Sp.-Promoter | gr. *melanogaster* |
|  | 578 | 633 | 2545 | 2600 | *hAT-1* | DRh | DNA Tr. | 0.7500 | d | 106* |  |  |
|  | 589 | 673 | 2556 | 2643 | *Hoana5* | DAna | DNA Tr./hAT | 0.8605 | c | 508 |  |  |
|  | 1318 | 1367 | 4297 | 4352 | *Gypsy-N1B* | RPr | LTR Retrotr. | 0.8200 | c | 227 | 5’UTRb |  |
| *D. ananassae* | 253 | 315 | 2404 | 2467 | *Gypsy1-LTR* | DMe | LTR Retrotr. | 0.6875 | d | 101* | Intergenic Sp. | gr. *ananassae* |
|  | 266 | 372 | 2417 | 2524 | *Gypsy20-I* | DYa | LTR Retrotr. | 0.6389 | d | 110* | Intergenic Sp. | gr*. ananassae* |
| *D. bipectinata* | 25 | 90 | 2133 | 2207 | *Transib-8* | DRh | DNA Tr. | 0.6857 | d | 105* | Intergenic Sp. | gr. *ananassae* |
|  | 133 | 220 | 2154 | 2264 | *Gypsy-37* | DEl | LTR Retrotr. | 0.6591 | c | 103* | Intergenic Sp. | gr. *ananassae* |
|  | 226 | 301 | 2361 | 2436 | *Transib-2* | DBi | DNA Tr. | 0.6316 | c | 98* | Intergenic Sp. | gr. *ananassae* |
|  | 396 | 445 | 2541 | 2595 | *BEL-17 LTR* | DTa | LTR Retrotr. | 0.6800 | d | 91* | Intergenic Sp. | gr. *ananassae,* |
| *D. kikkawai* | 1 | 59 | 2386 | 2444 | *Gypsy-2* | DBi | LTR Retrotr. | 0.5780 | d | 99* | Intergenic Sp. | *-* |
|  | 11 | 48 | 2396 | 2433 | *Transib-1* | DFi | DNA Tr. | 0.7368 | d | 105* | Intergenic Sp. | *-* |
|  | 47 | 132 | 2432 | 2517 | *Gypsy-24* | DY | LTR Retrotr. | 0.6047 | c | 115* | Intergenic Sp. | *-* |
| *D. busckii* | 14 | 83 | 2124 | 2193 | *Gypsy-1* | DBi | LTR Retrotr. | 0.7500 | d | 220 | Intergenic Sp. | *-* |
| *D. albomicans* | 223 | 313 | 2470 | 2560 | *Mariner-4* | DK | DNA Tr. | 0.6458 | d | 109* | Intergenic Sp. | *-* |
|  | 309 | 358 | 2556 | 2608 | *Gypsy-24* | DY | LTR Retrotr. | 0.6778 | c | 114* | Intergenic Sp., promoter | **S/gen. *Drosophila*** |
| *D. hydei* | 191 | 238 | 2572 | 2621 | *R1-4B* | DK | Non-LTR R. | 0.7083 | d | 101* | Intergenic Sp. | **S/gen. *Drosophila*** |
| *D. mojavensis* | 136 | 215 | 2408 | 2509 | *Jockey-7* | DEl | Non-LTR R. | 0.7073 | d | 113* | Intergenic Sp. | gr. *replete* |
|  | 167 | 241 | 2441 | 2535 | *hAT-2* | DY | DNA Tr. | 0.6933 | d | 107* | Intergenic Sp. | gr. *repleta* |
| *D. grimshawi* | 47 | 125 | 2282 | 2377 | *BEL-7* | DAn | LTR Retrotr. | 0.6582 | c | 100* | Intergenic Sp. | gr. *repleta, grimshawi* |
|  | 253 | 310 | 2535 | 2594 | *CR1-1* | DAn | Non-LTR R. | 0.6552 | d | 101* | Intergenic Sp. | **S/gen. *Drosophila*** |
| *D. kanekoi* | 593 | 673 | 634 | 782 | *hAT-8* | DBp | DNA Tr. | 0.7882 | c | 217 | Intergenic Sp. | s/gr. *virilis, kanekoi* |
|  | 1280 | 1342 | 1655 | 1732 | *Helitron-like-5* | Hmel | DNA Tr. | 0.7705 | c | 246 | Intergenic Sp. | s/gr. *virilis, kanekoi* |
|  | 2296 | 2402 | 3114 | 3282 | *L2-10* | Hmel | Non-LTR R. | 0.7723 | c | 250 | Intron1 | S/gen. *Drosophila, Dorsilopha* |
|  | 58 | 252 | 59 | 270 | *Inverted repeat* (YASS) | - | DNA Tr. (ancestral) | E_val_ = 1.50E-11 | c-d | 199 | Intergenic Sp. | Gr. *virilis* |
|  | 1842 | 2041 | 2363 | 2588 |  |  |  |  |  |  |  |  |
|  | 267 | 582 | 286 | 622 | *Inverted repeat* (YASS) | - | DNA Tr. (ancestral) | E_val_ = 1.03E-50 | c-d | 666 | Intergenic Sp. |  |
|  | 1626 | 1950 | 2142 | 2495 |  |  |  |  |  |  |  |  |
| *D. ezoana* | 540 | 700 | 554 | 888 | *DNA8-78* | AP | DNA Tr. | 0.7580 | d | 305 | Intergenic Sp. | s/gr. *virilis, kanekoi* |
|  | 769 | 943 | 1533 | 1708 | *Helitron-N10* | LMi | DNA Tr. | 0.7184 | c | 351 | Intergenic Sp. | s/gr. *virilis, kanekoi* |
|  | 1967 | 2056 | 2968 | 3261 | *Gypsy-22* | DWil | LTR Retrotr. | 0.7753 | d | 243 | Intron1 | S/gen. *Drosophila, Dorsilopha* |
|  | 115 | 263 | 116 | 268 | *Inverted repeat* (YASS) | - | DNA Tr. (ancestral) | E_val_ = 3.55E-09 | c-d | 169 | Intergenic Sp. | Gr. *virilis* |
|  | 1521 | 1661 | 2354 | 2535 |  |  |  |  |  |  |  |  |
|  | 272 | 576 | 277 | 762 | *Inverted repeat* (YASS) | - | DNA Tr. (ancestral) | E_val_ = 1.53E-29 | c-d | 412 | Intergenic Sp. |  |
|  | 1341 | 1634 | 2161 | 2503 |  |  |  |  |  |  |  |  |
| *D. littoralis* | 475 | 606 | 648 | 845 | *hAT-11* | LSal | DNA Tr. | 0.7385 | d | 214 | Intergenic Sp. | s/gr. *virilis, kanekoi* |
|  | 1060 | 1129 | 1659 | 1728 | *Helitron-N10* | LMi | DNA Tr. | 0.7639 | c | 270 | Intergenic Sp. |  |
|  | 1464 | 1501 | 2071 | 2108 | *hAT-N36* | LMi | DNA Tr. | 0.8684 | d | 229 | Intergenic Sp. |  |
|  | 2203 | 2292 | 2968 | 3261 | *Gypsy-22* | DWil | LTR Retrotr. | 0.7753 | d | 243 | Intron1 | S/gen. *Drosophila, Dorsilopha* |
|  | 212 | 508 | 367 | 738 | *Inverted repeat* (YASS*)* | - | DNA Tr. (ancestral) | E_val_ = 1.22E-44 | c-d | 595 | Intergenic Sp. | Gr. *virilis* |
|  | 1474 | 1774 | 2081 | 2388 |  |  |  |  |  |  |  |  |
|  | 103 | 228 | 104 | 383 | *Inverted repeat* (YASS) | - | DNA Tr. (ancestral) | E_val_ = 1.32E-06 | c-d | 140 | Intergenic Sp. |  |
|  | 1791 | 1903 | 2405 | 2541 |  |  |  |  |  |  |  |  |
| *D. lummei* | 488 | 541 | 2084 | 2137 | *Gypsy-1* | DFi | LTR Retrotr. | 0.7963 | c | 257 | Intergenic Sp. | s/gr. *virilis, kanekoi* |
|  | 2182 | 2271 | 2969 | 3262 | *Gypsy-22* | DWil | LTR Retrotr. | 0.7578 | d | 243 | Intron1 | S/gen. *Drosophila, Dorsilopha* |
|  | 256 | 569 | 287 | 618 | *Inverted repeat* (YASS) | - | DNA Tr. (ancestral) | E_val_ = 1.52E-19 | c-d | 294 | Intergenic Sp. | Gr. *virilis* |
|  | 1544 | 1829 | 2140 | 2488 |  |  |  |  |  |  |  |  |
|  | 134 | 242 | 135 | 272 | *Inverted repeat* (YASS*)* | - | DNA Tr. (ancestral) | E_val_ = 5.35E-11 | c-d | 192 | Intergenic Sp. | Gr. *virilis* |
|  | 1730 | 1829 | 2361 | 2488 |  |  |  |  |  |  |  |  |
|  | 552 | 633 | 601 | 738 | *Inverted repeat (*YASS*)* | - | DNA Tr. (ancestral) | E_val_ = 0.060 | c-d | 84 | Intergenic Sp. | Gr. *virilis* |
|  | 1485 | 1567 | 2081 | 2170 |  |  |  |  |  |  |  |  |
| *D. virilis* | 1393 | 1429 | 2967 | 3192 | *BEL-10* | DWil | LTR Retrotr. | 0.8684 | d | 228 | **Intron1** | S/gen. *Drosophila, Dorsilopha* |
|  | 77 | 144 | 78 | 145 | *Inverted repeat* (YASS) | - | DNA Tr. (ancestral) | E_val_ = 0.0005 | c-d | 104 | Intergenic Sp. | Gr. *virilis* |
|  | 1122 | 1056 | 2501 | 2569 |  |  |  |  |  |  |  |  |
|  | 181 | 346 | 409 | 596 | *Inverted repeat* (YASS) | - | DNA Tr. (ancestral) | E_val_ = 1.58E-13 | c-d | 217 | Intergenic Sp. | Gr. *virilis* |
|  | 931 | 765 | 2168 | 2366 |  |  |  |  |  |  |  |  |
|  | 119 | 231 | 120 | 468 | *Inverted repeat* (YASS*)* | - | DNA Tr. (ancestral) | E_val_ = 0.083 | c-d | 77 | Intergenic Sp. | Gr. *virilis* |
|  | 1057 | 1089 | 2502 | 2535 |  |  |  |  |  |  |  |  |
| ancestral *virilis-kanekoi* s/gr. | 535 | 575 | 552 | 602 | *Polinton-2* | DBi | DNA Tr. | 0.8537 | c | 205 | Intergenic Sp. | s/gr. *virilis, kanekoi* |
|  | 1054 | 1247 | 1533 | 1728 | *Helitron-N10* | LMi | DNA Tr. | 0.7202 | c | 372 | Intergenic Sp. |  |
|  | 1621 | 1714 | 2123 | 2220 | *Helitron-1* | DK | DNA Tr. | 0.6939 | d | 225 | Intergenic Sp. |  |
|  | 2310 | 2399 | 2969 | 3260 | *Gypsy-22* | DWil | LTR Retrotr. | 0.7753 | d | 243 | Intron1 | S/gen*. Drosophila, Dorsilopha* |

**Supplementary Table S1. Motifs of the *ras85D* noncoding regions and the upstream intergenic spacer of *Drosophila* species of different degrees of relationship that have homology to transposon sequences.** Homol. bound.1 – position of the motif on the sequence of an intergenic spacer of a particular species; Homol. bound.2 - position of the motif on the alignment of the intergenic spacer sequences of all species analyzed, taking deletions into account; counting from 5’-end of intergenic spacer sequences; Dir – orientation of the identified fragment: *d* – direct, *c* – complementary; Reg – gene region where the motif is located; Pr – promoter region. Int1 - intron 1 region; Intergenic Sp. – *ras85D* upstream intergenic spacer region; 5’UTRa - left part of the 5’UTR from TSS to intron 1. Source: DAna - *Drosophila ananassae*, DAzt - *Drosophila Azteca*, DBi - *Drosophila biarmipes*, DBp - *Drosophila bipectinate*, DBu - *Drosophila buzzatii*, DEl – *Drosophila elegans*; DFi - *Drosophila ficusphila*; DK - *Drosophila kikkawai*; DMe - *Drosophila melanogaster*; DMo - *Drosophila mojavensis*; DPe - *Drosophila persimilis*; DRh - *Drosophila rhopaloa*; DTa – *Drosophila takahashii*; DWil - *Drosophila willistoni*; DY – *Drosophila yakuba*; AP - *Acyrthosiphon pisum*; Hmel - *Heliconius Melpomene*; LMi - *Locusta migratoria*; LSal - *Lepeophtheirus salmonis*; PBa - *Pogonomyrmex barbatus*; Sin - *Solenopsis invicta*. The motifs of homology with transposons common for all groups of species were not taken into account. In the “Score” column the asterisk designates the values obtained using the YASS tool and the library of sequences of 57 transposon superfamilies from 52 *Drosophila* species of total amount - 2280 records.

| Positions | | | | Statistics | | | | Mutations | | | |  | Sp. |
| --- | --- | --- | --- | --- | --- | --- | --- | --- | --- | --- | --- | --- | --- |
| # | Repeat 1 | Repeat 2 | size | sense | E-value | score | bit-score | entropy | #ts | #tv | bias | bias.pr |  |
| 1 | (1087-1006) | (376-457) | 82/82 | reverse | 0.000 | 240 | 69.22 | 4.30 | 13 | 5 | 8, 6, 4 | 2.37E-02 | *D. lacicola* |
| 2 | (1301-1149) | (110-268) | 153/159 | reverse | 0.000 | 238 | 68.67 | 4.47 | 14 | 23 | 16, 11, 10 | 1.01E-02 |  |
| 3 | (155-254) | (273-374) | 100/102 | forward | 0.000 | 172 | 50.33 | 3.46 | 11 | 20 | 11, 8, 12 | 1.73E-02 |  |
| 4 | (1243-1168) | (299-374) | 76/76 | reverse | 0.000 | 163 | 47.82 | 3.85 | 10 | 12 | 7, 8, 7 | 3.50E-02 |  |
| 5 | (744-871) | (858-991) | 128/134 | forward | 0.000 | 109 | 32.82 | 4.40 | 18 | 26 | 14, 13, 17 | 1.40E-02 |  |
| 6 | (469-434) | (434-469) | 36/36 | Pal. | 0.021 | 86 | 26.43 | 3.45 | 2 | 6 | 3, 3, 2 | 8.54E-02 |  |
| 7 | (814-840) | (917-943) | 27/27 | forward | 6.691 | 56 | 18.09 | 3 | 4 | 4 | 2, 3, 3 | 8.54E-02 |  |
| 8 | (701-680) | (510-531) | 22/22 | reverse | 8.112 | 55 | 17.81 | 3.32 | 2 | 3 | 1, 2, 2 | 1.23E-01 |  |
| 9 | (1106-1058) | (854-902) | 49/49 | reverse | 9.835 | 54 | 17.54 | 3.12 | 4 | 16 | 9, 5, 6 | 2.23E-02 |  |
| 1 | (1291-1134) | (103-255) | 158/153 | reverse | 0.000 | 298 | 85.43 | 4.55 | 17 | 22 | 17, 9, 13 | 6.26E-03 | *D. montana* |
| 2 | (1072-991) | (363-444) | 82/82 | reverse | 0.000 | 265 | 76.25 | 4.43 | 12 | 3 | 7, 5, 3 | 2.51E-02 |  |
| 3 | (1244-1153) | (267-361) | 92/95 | reverse | 0.000 | 161 | 47.32 | 3.97 | 11 | 17 | 10, 11, 7 | 1.83E-02 |  |
| 4 | (154-241) | (271-361) | 88/91 | forward | 0.000 | 141 | 41.75 | 3.37 | 14 | 15 | 10, 10, 9 | 2.70E-02 |  |
| 5 | (729-862) | (843-982) | 134/140 | forward | 0.001 | 101 | 30.63 | 4.17 | 12 | 35 | 13, 20, 14 | 7.36E-03 |  |
| 6 | (562-406) | (406-562) | 157/157 | Pal. | 0.077 | 79 | 24.51 | 3.87 | 20 | 43 | 22, 19, 22 | 1.13E-02 |  |
| 7 | (530-494) | (494-530) | 37/37 | reverse | 0.077 | 79 | 24.51 | 2.92 | 2 | 7 | 1, 4, 4 | 3.20E-02 |  |
| 8 | (456-421) | (421-456) | 36/36 | Pal. | 0.640 | 68 | 21.45 | 3.52 | 2 | 8 | 4, 3, 3 | 7.11E-02 |  |
| 9 | (600-669) | (1220-1290) | 70/71 | forward | 1.141 | 65 | 20.61 | 3.03 | 14 | 16 | 9, 10, 11 | 2.45E-02 |  |
| 10 | (801-825) | (904-928) | 25/25 | forward | 1.384 | 64 | 20.33 | 3.32 | 2 | 4 | 2, 2, 2 | 1.23E-01 |  |
| 11 | (37-79) | (211-253) | 43/43 | forward | 5.336 | 57 | 18.39 | 2.81 | 11 | 7 | 6, 8, 4 | 2.37E-02 |  |
| 12 | (1002-1044) | (1066-1109) | 43/44 | forward | 7.847 | 55 | 17.83 | 3.17 | 4 | 11 | 6, 7, 2 | 1.26E-02 |  |
| 1 | (1829-1544) | (256-569) | 286/314 | reverse | 0.000 | 294 | 84.36 | 4.95 | 38 | 48 | 32, 27, 27 | 7.21E-03 | *D. lummei* |
| 2 | (101-246) | (223-364) | 146/142 | forward | 0.000 | 262 | 75.46 | 4.59 | 16 | 26 | 15, 13, 14 | 1.81E-02 |  |
| 3 | (1829-1730) | (134-242) | 100/109 | reverse | 0.000 | 192 | 55.97 | 4.63 | 12 | 13 | 10, 6, 9 | 1.93E-02 |  |
| 4 | (1567-1485) | (552-633) | 83/82 | reverse | 0.060 | 84 | 25.91 | 3.45 | 11 | 20 | 13, 10, 8 | 1.46E-02 |  |
| 5 | (566-606) | (1193-1234) | 41/42 | forward | 0.088 | 82 | 25.35 | 3.70 | 3 | 7 | 3, 4, 3 | 7.11E-02 |  |
| 6 | (868-816) | (816-868) | 53/53 | Pal. | 0.232 | 77 | 23.96 | 3.24 | 8 | 11 | 7, 7, 5 | 3.43E-02 |  |
| 7 | (729-774) | (928-975) | 46/48 | forward | 0.501 | 73 | 22.85 | 3.58 | 11 | 5 | 6, 8, 2 | 8.37E-03 |  |
| 8 | (1061-1013) | (194-245) | 49/52 | reverse | 1.595 | 67 | 21.18 | 3 | 3 | 13 | 6, 1, 9 | 1.86E-03 |  |
| 9 | (275-298) | (557-580) | 24/24 | forward | 1.595 | 67 | 21.18 | 3.17 | 2 | 4 | 1, 2, 3 | 8.23E-02 |  |
| 10 | (769-788) | (803-822) | 20/20 | forward | 2.346 | 65 | 20.62 | 2.92 | 1 | 2 | 1, 2, 0 | 1.11E-01 |  |
| 11 | (1882-1861) | (103-125) | 22/23 | reverse | 3.450 | 63 | 20.06 | 3.32 | 1 | 2 | 0, 2, 1 | 1.11E-01 |  |
| 12 | (1602-1622) | (1731-1751) | 21/21 | forward | 3.450 | 63 | 20.06 | 3 | 2 | 3 | 2, 2, 1 | 1.23E-01 |  |
| 13 | (613-565) | (178-229) | 49/52 | reverse | 4.185 | 62 | 19.79 | 3.08 | 8 | 8 | 6, 3, 7 | 2.23E-02 |  |
| 14 | (1819-1839) | (1864-1884) | 21/21 | forward | 7.465 | 59 | 18.95 | 2.95 | 3 | 2 | 3, 2, 0 | 4.12E-02 |  |
| 1 | (931-765) | (181-346) | 167/166 | reverse | 0.000 | 217 | 62.88 | 4.52 | 28 | 17 | 14, 13, 18 | 1.16E-02 | *D. virilis* |
| 2 | (1122-1056) | (77-144) | 67/68 | reverse | 0.001 | 104 | 31.45 | 4.25 | 7 | 12 | 6, 6, 7 | 4.01E-02 |  |
| 3 | (250-193) | (3-57) | 58/55 | reverse | 0.056 | 79 | 24.5 | 3.52 | 9 | 10 | 6, 8, 5 | 3.00E-02 |  |
| 4 | (1089-1057) | (199-231) | 33/33 | reverse | 0.083 | 77 | 23.94 | 3.12 | 3 | 6 | 4, 3, 2 | 6.40E-02 |  |
| 5 | (998-946) | (737-790) | 53/54 | reverse | 0.122 | 75 | 23.39 | 3.18 | 7 | 10 | 7, 5, 5 | 3.80E-02 |  |
| 6 | (993-1018) | (1010-1035) | 26/26 | forward | 0.122 | 75 | 23.39 | 3.24 | 2 | 3 | 1, 1, 3 | 8.23E-02 |  |
| 7 | (823-799) | (32-55) | 25/24 | reverse | 0.471 | 68 | 21.44 | 3.42 | 2 | 2 | 1, 2, 1 | 1.48E-01 |  |
| 8 | (293-336) | (362-406) | 44/45 | forward | 0.571 | 67 | 21.16 | 3.58 | 1 | 9 | 5, 1, 4 | 2.13E-02 |  |
| 9 | (99-116) | (890-907) | 18/18 | forward | 1.018 | 64 | 20.33 | 3.12 | 1 | 1 | 0, 0, 2 | 1.11E-01 |  |
| 10 | (981-942) | (942-981) | 40/40 | Pal. | 7.000 | 54 | 17.55 | 3.32 | 4 | 10 | 8, 3, 3 | 1.26E-02 |  |
| 1 | (1140-962) | (273-454) | 179/182 | reverse | 0.000 | 209 | 60.57 | 4.77 | 23 | 32 | 20, 19, 16 | 1.18E-02 | *D. americana* |
| 2 | (1234-1132) | (159-262) | 103/104 | reverse | 0.000 | 106 | 31.97 | 3.94 | 20 | 22 | 14, 14, 14 | 1.94E-02 |  |
| 3 | (579-531) | (531-579) | 49/49 | Pal. | 0.017 | 87 | 26.69 | 2.81 | 2 | 11 | 6, 1, 6 | 7.53E-03 |  |
| 4 | (1208-1167) | (138-183) | 42/46 | reverse | 0.025 | 85 | 26.14 | 3.51 | 5 | 5 | 4, 3, 3 | 7.11E-02 |  |
| 5 | (1232-1200) | (191-223) | 33/33 | reverse | 0.044 | 82 | 25.3 | 2.92 | 4 | 4 | 3, 3, 2 | 8.54E-02 |  |
| 6 | (1005-1025) | (1134-1154) | 21/21 | forward | 1.702 | 63 | 20.03 | 3 | 2 | 3 | 2, 2, 1 | 1.23E-01 |  |
| 7 | (64-22) | (22-64) | 43/43 | Pal. | 2.063 | 62 | 19.75 | 2.92 | 2 | 10 | 4, 4, 4 | 6.52E-02 |  |
| 8 | (484-514) | (907-936) | 31/30 | forward | 4.456 | 58 | 18.64 | 2.85 | 0 | 6 | 2, 3, 1 | 8.23E-02 |  |
| 9 | (948-921) | (463-490) | 28/28 | reverse | 6.549 | 56 | 18.08 | 3 | 4 | 4 | 1, 5, 2 | 2.56E-02 |  |
| 10 | (1222-1241) | (1267-1286) | 20/20 | forward | 9.625 | 54 | 17.53 | 3 | 3 | 2 | 3, 2, 0 | 4.12E-02 |  |
| 1 | (786-711) | (711-786) | 76/76 | Pal. | 0.000 | 368 | 104.99 | 5.05 | 0 | 0 | 0, 0, 0 | 1.00E+00 | *D. borealis* |
| 2 | (1103-1024) | (375-454) | 80/80 | reverse | 0.000 | 244 | 70.4 | 4.45 | 11 | 5 | 9, 3, 4 | 9.30E-03 |  |
| 3 | (1312-1160) | (129-272) | 153/144 | reverse | 0.000 | 223 | 64.56 | 4.25 | 17 | 25 | 15, 12, 15 | 1.57E-02 |  |
| 4 | (167-253) | (290-373) | 87/84 | forward | 0.000 | 186 | 54.26 | 3.74 | 8 | 13 | 5, 8, 8 | 2.50E-02 |  |
| 5 | (1270-1150) | (284-406) | 121/123 | reverse | 0.000 | 176 | 51.48 | 4.43 | 19 | 22 | 14, 14, 13 | 1.94E-02 |  |
| 6 | (277-247) | (247-277) | 31/31 | Pal. | 0.000 | 106 | 32.01 | 4.17 | 2 | 0 | 0, 0, 2 | 1.11E-01 |  |
| 7 | (1322-1285) | (103-141) | 38/39 | reverse | 0.002 | 99 | 30.07 | 3.64 | 4 | 4 | 3, 2, 3 | 8.54E-02 |  |
| 8 | (811-931) | (907-1030) | 121/124 | forward | 0.012 | 89 | 27.28 | 4.33 | 14 | 22 | 11, 14, 11 | 1.78E-02 |  |
| 9 | (371-340) | (340-371) | 32/32 | Pal. | 0.066 | 80 | 24.78 | 3 | 2 | 6 | 2, 3, 3 | 8.54E-02 |  |
| 10 | (68-17) | (17-68) | 52/52 | reverse | 0.098 | 78 | 24.22 | 2.92 | 6 | 14 | 7, 6, 7 | 3.82E-02 |  |
| 11 | (956-900) | (806-862) | 57/57 | reverse | 1.196 | 65 | 20.61 | 3.70 | 9 | 14 | 8, 8, 7 | 3.35E-02 |  |
| 12 | (418-459) | (901-944) | 42/44 | forward | 3.804 | 59 | 18.94 | 3.66 | 5 | 8 | 5, 6, 2 | 2.26E-02 |  |
| 13 | (489-448) | (448-489) | 42/42 | Pal. | 9.975 | 54 | 17.55 | 2.95 | 4 | 10 | 4, 6, 4 | 4.39E-02 |  |
| 1 | (1950-1626) | (267-582) | 325/316 | reverse | 0.000 | 666 | 188.07 | 5.29 | 48 | 40 | 31, 33, 24 | 4.32E-03 | *D. kanekoi* |
| 2 | (724-940) | (1010-1234) | 217/225 | forward | 0.000 | 343 | 98.08 | 4.95 | 30 | 38 | 24, 22, 22 | 1.14E-02 |  |
| 3 | (2041-1842) | (58-252) | 200/195 | reverse | 0.000 | 199 | 57.97 | 4.58 | 26 | 37 | 20, 22, 21 | 1.24E-02 |  |
| 4 | (177-265) | (290-383) | 89/94 | forward | 0.000 | 145 | 42.92 | 3.73 | 13 | 15 | 9, 7, 12 | 1.52E-02 |  |
| 5 | (679-628) | (628-679) | 52/52 | Pal. | 0.005 | 97 | 29.55 | 2.97 | 0 | 9 | 2, 7, 0 | 1.83E-03 |  |
| 6 | (858-801) | (801-858) | 58/58 | Pal. | 0.014 | 92 | 28.16 | 3.73 | 8 | 12 | 6, 7, 7 | 3.82E-02 |  |
| 7 | (579-618) | (1380-1414) | 40/35 | forward | 0.066 | 84 | 25.93 | 3.12 | 2 | 2 | 2, 1, 1 | 1.48E-01 |  |
| 8 | (446-348) | (348-446) | 99/99 | Pal. | 0.118 | 81 | 25.09 | 4 | 16 | 31 | 15, 15, 17 | 1.60E-02 |  |
| 9 | (233-208) | (208-233) | 26/26 | Pal. | 0.457 | 74 | 23.14 | 2.92 | 0 | 4 | 0, 2, 2 | 7.41E-02 |  |
| 10 | (948-986) | (1455-1493) | 39/39 | forward | 0.457 | 74 | 23.14 | 3.17 | 5 | 7 | 4, 2, 6 | 2.61E-02 |  |
| 11 | (749-694) | (414-469) | 56/56 | reverse | 1.457 | 68 | 21.47 | 2.81 | 11 | 12 | 8, 5, 10 | 1.56E-02 |  |
| 12 | (827-858) | (982-1013) | 32/32 | forward | 1.767 | 67 | 21.19 | 2.95 | 5 | 4 | 4, 4, 1 | 3.20E-02 |  |
| 13 | (821-891) | (1453-1523) | 71/71 | forward | 1.767 | 67 | 21.19 | 3.52 | 11 | 19 | 11, 11, 8 | 2.01E-02 |  |
| 14 | (1152-1110) | (1110-1152) | 43/43 | reverse | 3.826 | 63 | 20.08 | 3.52 | 2 | 13 | 5, 5, 5 | 5.27E-02 |  |
| 15 | (874-831) | (595-638) | 44/44 | reverse | 4.642 | 62 | 19.8 | 3.34 | 5 | 10 | 4, 7, 4 | 3.14E-02 |  |
| 1 | (1634-1341) | (272-576) | 294/305 | reverse | 0.000 | 412 | 117.24 | 5.16 | 33 | 44 | 26, 27, 24 | 9.73E-03 | *D. ezoana* |
| 2 | (166-273) | (281-389) | 108/109 | forward | 0.000 | 236 | 68.23 | 4.17 | 11 | 15 | 10, 9, 7 | 2.39E-02 |  |
| 3 | (1661-1521) | (115-263) | 141/149 | reverse | 0.000 | 169 | 49.58 | 4.47 | 19 | 22 | 12, 15, 14 | 1.68E-02 |  |
| 4 | (145-100) | (100-145) | 46/ 46 | Pal. | 0.010 | 92 | 28.14 | 3.75 | 6 | 8 | 5, 4, 5 | 5.27E-02 |  |
| 5 | (545-626) | (564-646) | 82/ 83 | forward | 0.084 | 81 | 25.08 | 3.01 | 6 | 17 | 5, 8, 10 | 1.56E-02 |  |
| 6 | (64-22) | (22-64) | 43/ 43 | Pal. | 0.102 | 80 | 24.8 | 3.25 | 2 | 8 | 2, 4, 4 | 5.33E-02 |  |
| 7 | (9-68) | (1459-1518) | 60/ 60 | forward | 0.183 | 77 | 23.97 | 3.55 | 12 | 7 | 7, 7, 5 | 3.43E-02 |  |
| 8 | (1204-1161) | (1161-1204) | 44/ 44 | Pal. | 0.705 | 70 | 22.02 | 3.5 | 4 | 12 | 4, 6, 6 | 3.91E-02 |  |
| 9 | (191-285) | (1316-1410) | 95/ 95 | forward | 1.258 | 67 | 21.18 | 3.57 | 16 | 20 | 14, 9, 13 | 1.26E-02 |  |
| 10 | (990-937) | (147-199) | 54/ 53 | reverse | 1.526 | 66 | 20.9 | 3 | 6 | 13 | 7, 5, 7 | 3.43E-02 |  |
| 11 | (864-814) | (222-271) | 51/ 50 | reverse | 1.850 | 65 | 20.63 | 3.70 | 5 | 13 | 7, 3, 8 | 1.36E-02 |  |
| 12 | (314-362) | (1524-1570) | 49/ 47 | forward | 2.722 | 63 | 20.07 | 3.64 | 3 | 9 | 3, 5, 4 | 5.22E-02 |  |
| 13 | (548-494) | (494-548) | 55/ 55 | Pal. | 4.004 | 61 | 19.51 | 3.32 | 12 | 11 | 7, 8, 8 | 3.35E-02 |  |
| 14 | (1443-1419) | (433-457) | 25/ 25 | reverse | 4.857 | 60 | 19.23 | 3 | 2 | 5 | 2, 3, 2 | 9.60E-02 |  |
| 15 | (1623-1609) | (1097-1111) | 15/ 15 | reverse | 8.665 | 57 | 18.4 | 3.12 | 1 | 0 | 0, 0, 1 | 3.33E-01 |  |
| 1 | (1774-1474) | (212-508) | 301/297 | reverse | 0.000 | 595 | 167.76 | 5.31 | 24 | 50 | 24, 32, 18 | 1.56E-03 | *D. littoralis* |
| 2 | (686-752) | (867-936) | 67/ 70 | forward | 0.000 | 164 | 48.07 | 4.07 | 5 | 9 | 1, 8, 5 | 3.77E-03 |  |
| 3 | (1903-1791) | (103-228) | 113/126 | reverse | 0.000 | 140 | 41.41 | 4.64 | 12 | 14 | 9, 10, 7 | 2.39E-02 |  |
| 4 | (774-844) | (866-935) | 71/ 70 | forward | 0.000 | 126 | 37.52 | 3.55 | 8 | 13 | 9, 8, 4 | 1.39E-02 |  |
| 5 | (931-889) | (889-931) | 43/ 43 | Pal. | 0.000 | 114 | 34.19 | 4.18 | 0 | 2 | 1, 1, 0 | 2.22E-01 |  |
| 6 | (593-470) | (470-593) | 124/124 | Pal. | 0.002 | 102 | 30.86 | 3.12 | 10 | 38 | 15, 18, 15 | 1.42E-02 |  |
| 7 | (932-888) | (796-841) | 45/46 | reverse | 0.002 | 101 | 30.58 | 3.43 | 1 | 9 | 3, 2, 5 | 4.27E-02 |  |
| 8 | (792-854) | (1259-1319) | 63/ 61 | forward | 0.052 | 85 | 26.13 | 3.91 | 14 | 6 | 7, 5, 8 | 2.86E-02 |  |
| 9 | (1583-1510) | (1510-1583) | 74/ 74 | Pal. | 0.063 | 84 | 25.86 | 3.06 | 10 | 18 | 10, 9, 9 | 2.79E-02 |  |
| 10 | (530-458) | (458-530) | 73/ 73 | Pal. | 0.077 | 83 | 25.58 | 2.82 | 4 | 21 | 7, 11, 7 | 1.81E-02 |  |
| 11 | (584-475) | (475-584) | 110/110 | Pal. | 0.202 | 78 | 24.19 | 3.46 | 6 | 32 | 13, 12, 13 | 2.08E-02 |  |
| 12 | (931-881) | (708-755) | 51/ 48 | reverse | 0.359 | 75 | 23.36 | 3.68 | 5 | 9 | 5, 5, 4 | 5.27E-02 |  |
| 13 | (1032-954) | (954-1032) | 79/ 79 | Pal. | 0.359 | 75 | 23.36 | 2.92 | 10 | 21 | 9, 13, 9 | 1.62E-02 |  |
| 14 | (1204-1241) | (1227-1262) | 38/ 36 | forward | 0.359 | 75 | 23.36 | 3.08 | 3 | 6 | 2, 4, 3 | 6.40E-02 |  |
| 15 | (1586-1563) | (1563-1586) | 24/ 24 | Pal. | 1.382 | 68 | 21.41 | 2.92 | 4 | 0 | 2, 2, 0 | 7.41E-02 |  |
| 16 | (1945-1900) | (1397-1442) | 46/ 46 | reverse | 1.675 | 67 | 21.14 | 3.10 | 7 | 7 | 5, 1, 8 | 3.77E-03 |  |
| 17 | (1501-1465) | (502-538) | 37/ 37 | reverse | 2.031 | 66 | 20.86 | 2.87 | 3 | 7 | 5, 1, 4 | 2.13E-02 |  |
| 18 | (590-647) | (1274-1333) | 58/ 60 | forward | 2.031 | 66 | 20.86 | 3.33 | 6 | 14 | 9, 3, 8 | 7.95E-03 |  |
| 19 | (84-123) | (898-937) | 40/ 40 | forward | 2.462 | 65 | 20.58 | 2.85 | 3 | 10 | 5, 4, 4 | 5.65E-02 |  |
| 20 | (1277-1259) | (781-799) | 19/ 19 | reverse | 6.445 | 60 | 19.19 | 3.17 | 0 | 3 | 1, 0, 2 | 1.11E-01 |  |
| 21 | (618-657) | (1121-1160) | 40/ 40 | forward | 6.445 | 60 | 19.19 | 3.28 | 2 | 12 | 4, 6, 4 | 4.39E-02 |  |

**Supplementary Table S2. Location of direct and inverted repeats and palindromes in the intergenic spacer of the *Drosophila* *virilis* species group.** Counting from 5’-end of the *ras85D* upstream intergenic spacer sequences. Pal. – palindrome.


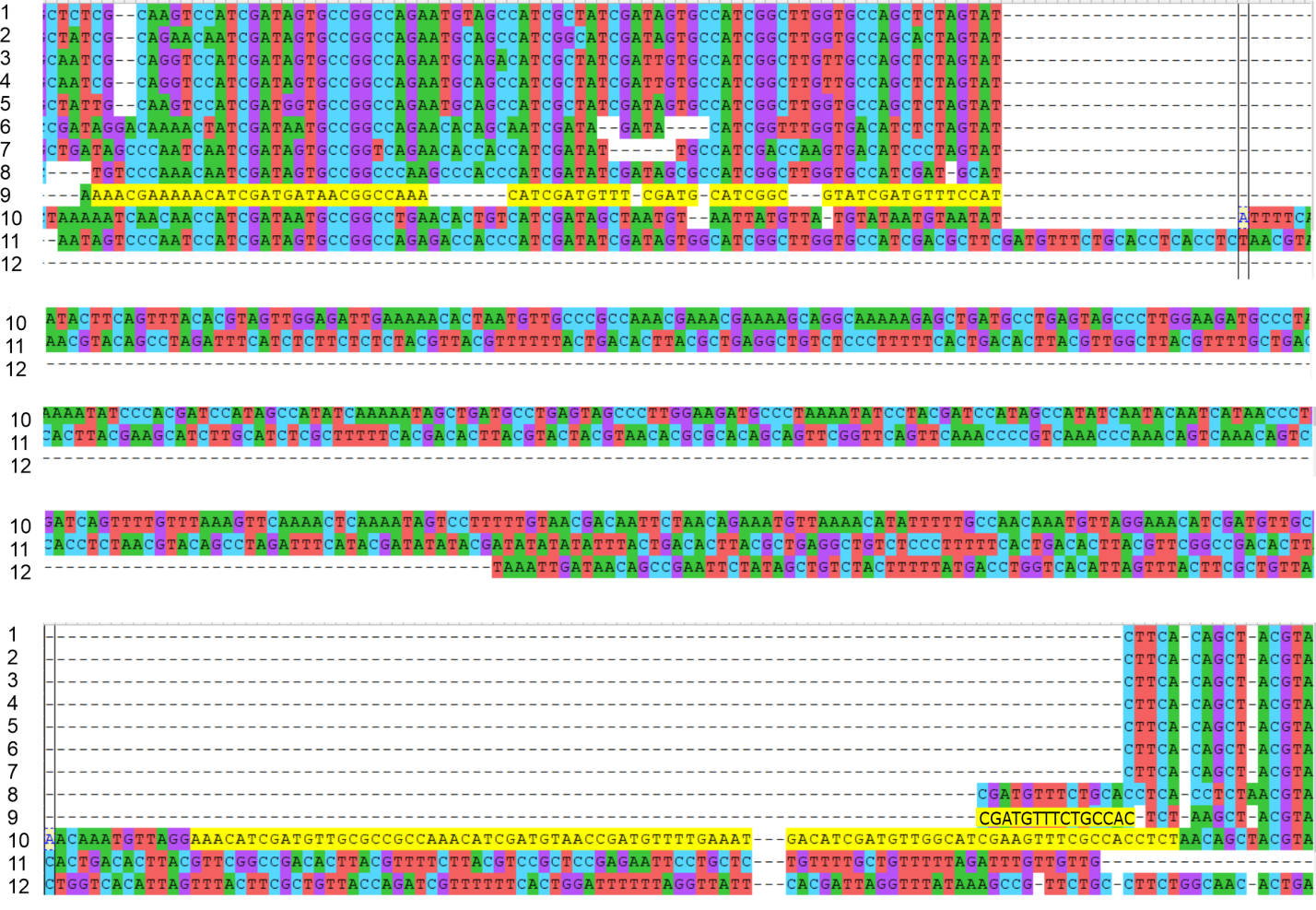


**Supplementary Figure S4. An example of the alignment of the promoter region in twelve species of the *D. melanogaster* species group.** Numbers of sequences correspond to the following species: *D. erecta* (1), *D. yakuba* (2), *D. sechellia* (3), *D. simulans* (4), *D. melanogaster* (5), *D. rhopaloa* (6), *D. ficusphila* (7), *D. biarmipes* (8), *D. takahashii* (9), *D. elegans* (10), *D. suzukii* (11), *D. eugracilis* (12). The similarity of *D. takahashii* and *D. elegans* promoter regions with DNA transposons of the *hAT* superfamily is highlighted.

| ## | *D. virilis* | | | | | | | | | | | Inverted repeat | | | | |
| --- | --- | --- | --- | --- | --- | --- | --- | --- | --- | --- | --- | --- | --- | --- | --- | --- |
|  | Sequence ID | genome scaffold | align. | mis mat. | Gap opens | START | END | E-value | Score | Identity % | Str. | species | repeate | Start | End | N** |
| * | NW_002014424.1 | 13047 | 384 | 0 | 0 | 4177220 | 4177603 | 0 | 693 | 100 | + | *D.virilis* | Ir-a | 1 | 384 | all |
|  | NW_002014424.1 | 13047 | 369 | 0 | 0 | 4178009 | 4178377 | 0 | 666 | 100 | - | *D.virilis* | Ir-b | 1 | 369 | all |
| 1 | NW_002014452.1 | 12726 | 79 | 17 | 1 | 1404288 | 1404360 | 0.032 | 41 | 70.886 | + | *D.ezoana* | Ir-b | 46 | 124 | 2 |
| 2 | NW_002014489.1 | 12734 | 173 | 25 | 2 | 476534 | 476676 | 1.04E-14 | 82.4 | 68.208 | + | *D.lacicola* | Ir-b | 28 | 200 | 6 |
|  | NW_002014489.1 | 12734 | 243 | 40 | 9 | 476703 | 476500 | 9.09E-13 | 77 | 67.49 | - | *D.lummei* | Ir-a | 265 | 507 | 6 |
| 3 | NW_002014485.1 | 12736 | 84 | 13 | 1 | 158447 | 158528 | 1.80E-14 | 82.4 | 82.143 | + | *D.littoralis* | Ir-a | 316 | 399 | 5 |
|  | NW_002014485.1 | 12736 | 105 | 22 | 1 | 158524 | 158422 | 1.35E-13 | 78.8 | 77.143 | - | *D.kanekoi* | Ir-b | 20 | 124 | 5 |
| 4 | NW_002014487.1 | 12799 | 74 | 13 | 1 | 501446 | 501518 | 2.45E-10 | 68 | 81.081 | + | *D.kanekoi* | Ir-b | 21 | 94 | 4 |
|  | NW_002014487.1 | 12799 | 142 | 33 | 4 | 501520 | 501383 | 9.26E-06 | 53.6 | 69.718 | - | *D.ezoana* | Ir-a | 449 | 584 | 6 |
| 5 | NW_00`2014432.1 | 12855 | 190 | 37 | 7 | 4860018 | 4860205 | 2.00E-17 | 91.5 | 72.632 | + | *D.lacicola* | Ir-b | 121 | 297 | 4 |
|  | NW_002014432.1 | 12855 | 146 | 42 | 1 | 4860205 | 4860061 | 1.47E-09 | 66.2 | 70.548 | - | *D.ezoana* | Ir-a | 103 | 248 | 3 |
| 6 | NW_002014432.1 | 12855 | 82 | 11 | 1 | 10114265 | 10114339 | 7.01E-11 | 69.8 | 78.049 | + | *D.kanekoi* | Ir-b | 5 | 86 | 6 |
|  | NW_002014432.1 | 12855 | 286 | 59 | 13 | 10114340 | 10114065 | 4.24E-16 | 87.8 | 69.231 | - | *D.littoralis* | Ir-a | 329 | 595 | 5 |
| 7 | NW_002014420.1 | 12875 | 75 | 15 | 2 | 9601494 | 9601566 | 9.5 | 33.7 | 70.667 | + | *D.lacicola* | Ir-a | 102 | 171 | 2 |
| 8 | NW_002014420.1 | 12875 | 254 | 49 | 7 | 11884893 | 11885143 | 5.89E-27 | 123 | 71.654 | + | *D.littoralis* | Ir-a | 92 | 325 | 6 |
|  | NW_002014420.1 | 12875 | 291 | 58 | 7 | 11885183 | 11884904 | 7.48E-36 | 152 | 72.509 | - | *D.kanekoi* | Ir-b | 40 | 319 | 6 |
| 9 | NW_002014420.1 | 12875 | 127 | 41 | 0 | 12621030 | 12621156 | 0.003 | 44.6 | 67.717 | + | *D.kanekoi* | Ir-b | 124 | 250 | 1 |
|  | NW_002014420.1 | 12875 | 75 | 21 | 0 | 12621156 | 12621082 | 0.058 | 41 | 72 | - | *D.ezoana* | Ir-a | 299 | 373 | 2 |
| 10 | NW_002014449.1 | 12958 | 310 | 76 | 7 | 698577 | 698881 | 8.73E-25 | 116 | 69.355 | + | *D.littoralis* | Ir-a | 128 | 423 | 6 |
|  | NW_002014449.1 | 12958 | 292 | 59 | 7 | 698833 | 698552 | 2.00E-36 | 154 | 72.603 | - | *D.lacicola* | Ir-b | 17 | 297 | 6 |
| 11 | NW_002014449.1 | 12958 | 248 | 60 | 10 | 1921861 | 1922104 | 0.001 | 46.4 | 66.129 | + | *D.littoralis* | Ir-a | 93 | 320 | 2 |
|  | NW_002014449.1 | 12958 | 200 | 44 | 4 | 1922126 | 1921932 | 5.02E-25 | 116 | 74 | - | *D.lacicola* | Ir-b | 50 | 246 | 1 |
| 12 | NW_002014421.1 | 12963 | 151 | 21 | 1 | 1014649 | 1014795 | 9.53E-38 | 159 | 83.444 | + | *D.lacicola* | Ir-a | 516 | 666 | 1 |
| 13 | NW_002014421.1 | 12963 | 118 | 25 | 3 | 13713150 | 13713255 | 0.058 | 41 | 67.797 | + | *D.ezoana* | Ir-a | 509 | 625 | 1 |
| 14 | NW_002014421.1 | 12963 | 95 | 19 | 0 | 14269443 | 14269537 | 9.12E-16 | 86 | 80 | + | *D.kanekoi* | Ir-b | 5 | 99 | 5 |
|  | NW_002014421.1 | 12963 | 123 | 27 | 4 | 14269537 | 14269418 | 7.60E-07 | 57.2 | 72.358 | - | *D.ezoana* | Ir-a | 446 | 564 | 4 |
| 15 | NW_002014421.1 | 12963 | 205 | 42 | 0 | 17803679 | 17803883 | 1.54E-44 | 181 | 79.512 | + | *D.kanekoi* | Ir-b | 5 | 209 | 6 |
|  | NW_002014421.1 | 12963 | 223 | 52 | 2 | 17803883 | 17803665 | 5.73E-28 | 127 | 72.646 | - | *D.lummei* | Ir-a | 314 | 531 | 6 |
| 16 | NW_002014421.1 | 12963 | 62 | 11 | 3 | 18644311 | 18644252 | 0.2 | 39.2 | 77.419 | - | *D.ezoana* | Ir-a | 78 | 138 | 4 |
| 17 | NW_002014480.1 | 12967 | 143 | 20 | 1 | 445287 | 445149 | 1.72E-34 | 149 | 83.217 | - | *D.lacicola* | Ir-a | 518 | 660 | 1 |
| 18 | NW_002014431.1 | 12970 | 122 | 25 | 3 | 269436 | 269544 | 2.10E-04 | 48.2 | 68.852 | + | *D.lummei* | Ir-b | 63 | 184 | 5 |
| 19 | NW_002014431.1 | 12970 | 69 | 9 | 2 | 497067 | 497133 | 2.45E-10 | 68 | 84.058 | + | *D.kanekoi* | Ir-b | 20 | 88 | 4 |
|  | NW_002014431.1 | 12970 | 59 | 9 | 2 | 497123 | 497066 | 1.13E-04 | 50 | 81.356 | - | *D.ezoana* | Ir-a | 469 | 526 | 6 |
| 20 | NW_002014431.1 | 12970 | 71 | 12 | 0 | 963997 | 963927 | 1.54E-12 | 75.2 | 83.099 | - | *D.lacicola* | Ir-b | 133 | 203 | 4 |
| 21 | NW_002014431.1 | 12970 | 206 | 38 | 6 | 1063916 | 1064104 | 6.29E-14 | 80.6 | 68.932 | + | *D.littoralis* | Ir-a | 140 | 336 | 4 |
|  | NW_002014431.1 | 12970 | 145 | 34 | 0 | 1064106 | 1063962 | 8.00E-23 | 109 | 76.552 | - | *D.kanekoi* | Ir-b | 77 | 221 | 6 |
| 22 | NW_002014431.1 | 12970 | 241 | 64 | 5 | 4185109 | 4185342 | 2.62E-16 | 87.8 | 69.295 | + | *D.littoralis* | Ir-b | 20 | 257 | 3 |
|  | NW_002014431.1 | 12970 | 79 | 21 | 0 | 4185214 | 4185136 | 3.72E-04 | 48.2 | 73.418 | - | *D.littoralis* | Ir-a | 287 | 365 | 6 |
| 23 | NW_002014431.1 | 12970 | 107 | 23 | 1 | 6666632 | 6666526 | 1.36E-13 | 78.8 | 76.636 | - | *D.littoralis* | Ir-b | 25 | 129 | 4 |
|  | NW_002014431.1 | 12970 | 109 | 25 | 3 | 6667200 | 6667304 | 7.46E-04 | 46.4 | 70.642 | + | *D.lacicola* | Ir-b | 123 | 228 | 4 |
| 24 | NW_002014431.1 | 12970 | 90 | 22 | 1 | 8664585 | 8664497 | 1.44E-06 | 55.4 | 74.444 | - | *D.lacicola* | Ir-b | 129 | 218 | 2 |
|  | NW_002014431.1 | 12970 | 74 | 11 | 2 | 8664725 | 8664794 | 6.57E-05 | 50 | 75.676 | + | *D.kanekoi* | Ir-b | 28 | 98 | 3 |
| 25 | NW_002014431.1 | 12970 | 192 | 37 | 2 | 9126390 | 9126570 | 2.45E-29 | 131 | 75 | + | *D.kanekoi* | Ir-b | 20 | 211 | 4 |
|  | NW_002014431.1 | 12970 | 278 | 64 | 5 | 9126654 | 9126389 | 3.05E-24 | 114 | 69.424 | - | *D.littoralis* | Ir-a | 128 | 396 | 6 |
| 26 | NW_002014442.1 | 13042 | 232 | 58 | 9 | 2694454 | 2694680 | 2.90E-06 | 55.4 | 67.672 | + | *D.kanekoi* | Ir-a | 324 | 543 | 2 |
|  | NW_002014442.1 | 13042 | 203 | 47 | 3 | 2694642 | 2694441 | 2.78E-28 | 127 | 74.877 | - | *D.lacicola* | Ir-b | 3 | 202 | 4 |
| 27 | NW_002014424.1 | 13047 | 130 | 31 | 2 | 452295 | 452423 | 3.00E-09 | 64.4 | 71.538 | + | *D.virilis* | Ir-a | 182 | 306 | 5 |
|  | NW_002014424.1 | 13047 | 117 | 17 | 1 | 452430 | 452315 | 2.78E-28 | 127 | 84.615 | - | *D.ezoana* | Ir-b | 8 | 124 | 5 |
| 28 | NW_002014424.1 | 13047 | 247 | 75 | 3 | 467096 | 467341 | 9.89E-12 | 73.4 | 67.206 | + | *D.ezoana* | Ir-a | 293 | 534 | 6 |
|  | NW_002014424.1 | 13047 | 294 | 74 | 6 | 467341 | 467055 | 2.99E-28 | 127 | 70.748 | - | *D.littoralis* | Ir-b | 11 | 299 | 6 |
| 29 | NW_002014424.1 | 13047 | 231 | 64 | 2 | 498585 | 498355 | 3.31E-18 | 95.1 | 69.264 | - | *D.kanekoi* | Ir-a | 268 | 491 | 3 |
| 30 | NW_002014419.1 | 13049 | 73 | 17 | 1 | 2231608 | 2231679 | 2.14E-04 | 48.2 | 75.342 | + | *D.lacicola* | Ir-b | 150 | 222 | 3 |
| 31 | NW_002014419.1 | 13049 | 251 | 66 | 2 | 18196251 | 18196494 | 1.27E-26 | 122 | 70.916 | + | *D.littoralis* | Ir-b | 5 | 255 | 4 |
|  | NW_002014419.1 | 13049 | 272 | 76 | 7 | 18196494 | 18196231 | 1.20E-10 | 69.8 | 67.279 | - | *D.ezoana* | Ir-a | 294 | 560 | 6 |
| 32 | NW_002014419.1 | 13049 | 133 | 23 | 1 | 24100300 | 24100431 | 1.61E-28 | 129 | 81.955 | + | *D.lacicola* | Ir-a | 511 | 643 | 1 |
| 33 | NW_002014450.1 | 13050 | 229 | 45 | 4 | 2086598 | 2086808 | 5.39E-25 | 116 | 71.179 | + | *D.kanekoi* | Ir-b | 40 | 265 | 4 |
|  | NW_002014450.1 | 13050 | 246 | 46 | 8 | 2086837 | 2086605 | 9.97E-18 | 93.3 | 69.106 | - | *D.littoralis* | Ir-a | 140 | 368 | 4 |
| 34 | NW_002014457.1 | 13052 | 79 | 10 | 1 | 548036 | 548112 | 9.15E-16 | 86 | 84.81 | + | *D.littoralis* | Ir-b | 5 | 83 | 6 |
|  | NW_002014457.1 | 13052 | 92 | 15 | 2 | 548105 | 548015 | 1.80E-14 | 82.4 | 81.522 | - | *D.littoralis* | Ir-a | 341 | 431 | 6 |
| 35 | NW_002014457.1 | 13052 | 54 | 7 | 1 | 997110 | 997057 | 1.44E-06 | 55.4 | 83.333 | - | *D.lacicola* | Ir-b | 86 | 137 | 3 |
| 36 | NW_002014457.1 | 13052 | 244 | 44 | 9 | 1233146 | 1233368 | 1.02E-14 | 82.4 | 68.443 | + | *D.lummei* | Ir-b | 4 | 235 | 6 |
|  | NW_002014457.1 | 13052 | 212 | 49 | 4 | 1233278 | 1233078 | 5.51E-21 | 104 | 71.698 | - | *D.littoralis* | Ir-a | 277 | 488 | 6 |
| 37 | NW_002014453.1 | 13246 | 97 | 18 | 3 | 2466573 | 2466480 | 1.39E-09 | 66.2 | 77.32 | - | *D.littoralis* | Ir-a | 348 | 443 | 5 |

**Supplementary Table S3. Spread of sequences homologous to inverted repeats (Ir-a and Ir-b) of six species of the *virilis* group (*D. lacicola, D. kanekoi, D. littoralis, D. ezoana, D. lummei, D. virilis*) in *D. virilis* genome.** All sequences are located within non-coding DNA. Ir-a - 5'-inverted repeat; Ir-b - 3'-inverted repeat. N ** - The number of species from the indicated ones, in which the sequences of inverted repeats confirm homology with this fragment of the *D. virilis* genome. * - the inverted repeats Ir-a and Ir-b of *D. virilis* themselves.


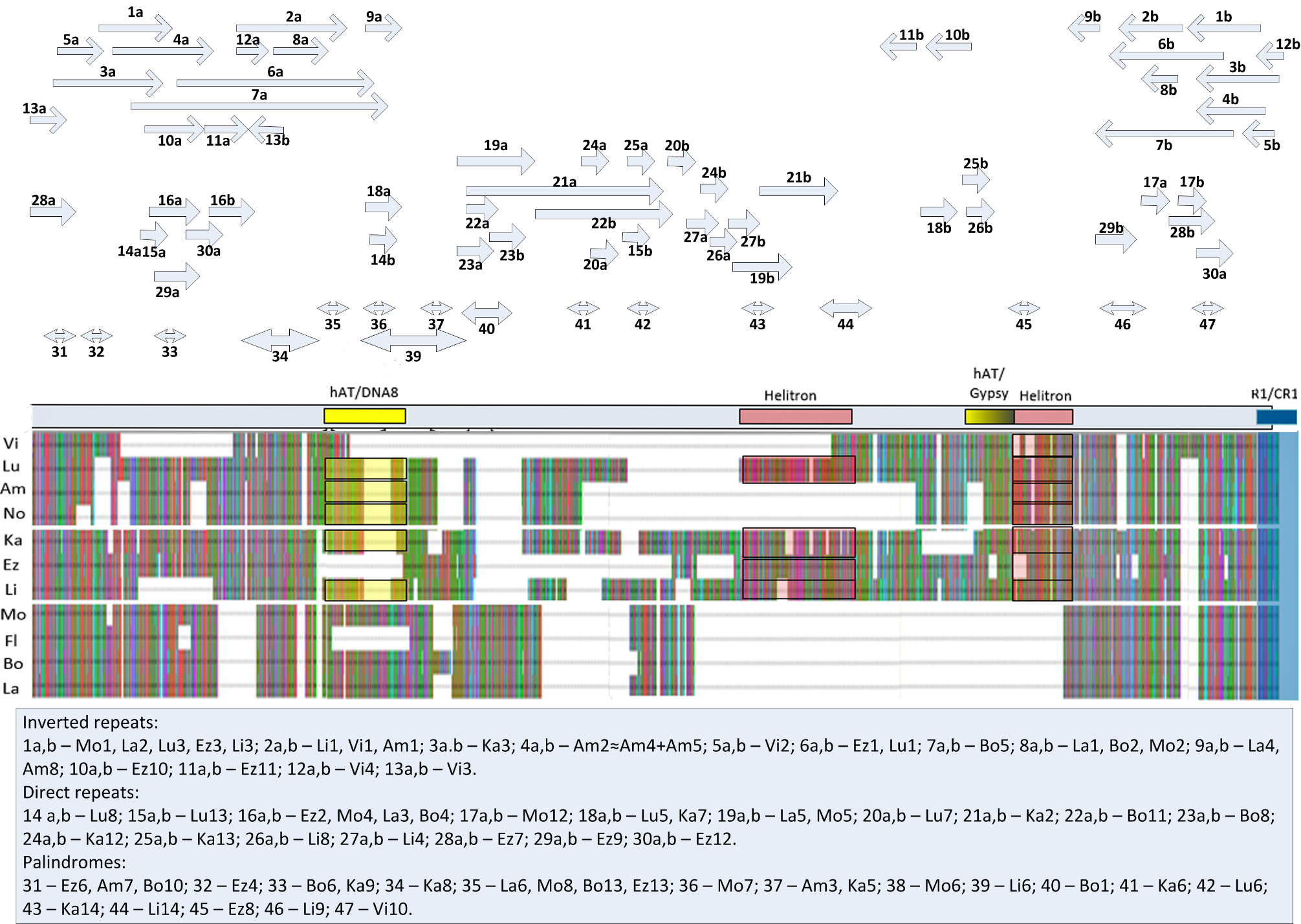


**Supplementary Figure S5. The structure of the *ras85D* upstream intergenic spacer in *Drosophila* species of the *virili*s group.** Arrows indicate pairs of direct and inverted repeats, as well as palindromes identified in the *ras85D* upstream intergenic spacer in *Drosophila* species of the *virilis* group. Homologous repeats are designated with the same numbers. The distal repeat of each pair is designated with «а», the proximal one - with «b».  Location of the repeats on the sequence shown in Table S2. Counting from 5’-end of intergenic spacer sequences. Vi – *D. virilis*; Lu – *D. lummei*; Am – *D. americana*; No – *D. novamexicana*; Ka – *D. kanekoi*; Ez – *D. ezoana*; Li – *D. littoralis*; Mo – *D. montana*; Fl – *D. flavomontana*; Bo – *D. borealis*; La – *D. lacicola*

| Positions | | | | Statistics | | | | Mutations | | | | |
| --- | --- | --- | --- | --- | --- | --- | --- | --- | --- | --- | --- | --- |
| # | Rlb1 _Caf155 | *D.americana*  ir-b | size | sense | E-value | score | bit-score | entropy | #ts | #tv | bias | bias.pr |
| 1 | (892-1084) | (93-291) | 193/ 199 | f | 2.4e-15 | 235 | 68.1 | 4.550 | 9 | 6 | 6, 5, 4 | 4.39e-02 |
| 2 | (1040-1123) | (332-415) | 84/84 | f | 1.2e-12 | 203 | 59.1 | 4.134 | 11 | 13 | 12, 5, 7 | 7.58e-03 |
| 3 | (1184-1289) | (423-534) | 106/ 112 | f | 1.6e-05 | 118 | 35.4 | 4.512 | 12 | 20 | 11, 12, 9 | 2.05e-02 |
|  | Rlb1_ Caf155 | *D.americana*  ir-a | size | sense | E-value | score | bit-score | entropy | #ts | #tv | bias | bias.pr |
| 1 | (855-749) | (5-112) | 108/107 | r | 0.00108751 | 94 | 28.74 | 3.78212 | 14 | 17 | 8, 12, 11 | 1.73e-02 |

**Supplementary Table S4. The sequences of the *Caf1-55 - Rlb1* intergenic spacer of *D. americana* homologous to ir-a и ir-b repeats.** Identification of homologous fragments and assessment of their similarity were obtained using the YASS tool. The statistics of the significance and characteristics of the revealed mutational differences are presented. The p-statistics are given for this sequence. The results are presented on Figure S6.


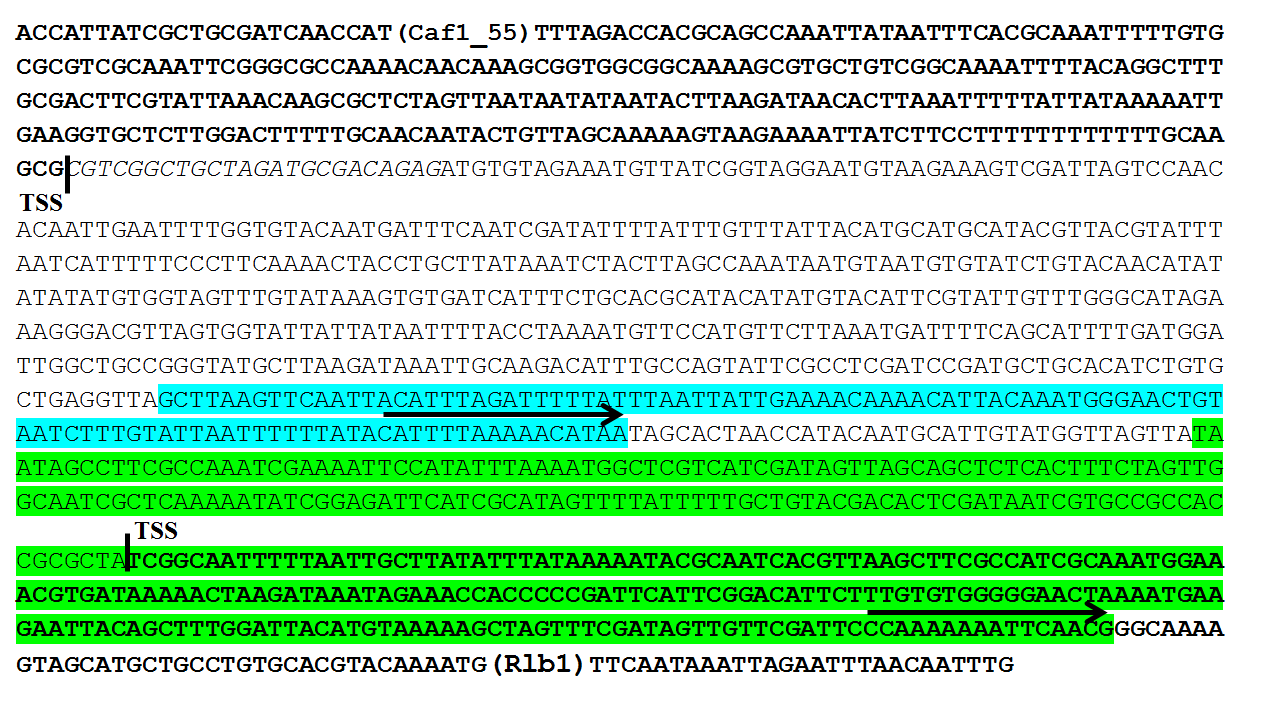


**Supplementary Figure S6. Sequences of the *Caf1-55 - Rlb1* intergenic spacer of *D. americana* homologous to Ir-a and Ir-b.** Sequences of the *Caf1-55* and *Rlb1* genes are highlighted in bold. Areas of homology to Ir-a and Ir-b are highlighted in light-blue and green respectively. Arrows shows the direction of the repeats.

| Species | Entrez SRA DB | Entrez EST DB | Entrez TSA DB | Entrez Nucleotide DB |
| --- | --- | --- | --- | --- |
| *D. erecta* | DRR129468, SRR6968105, SRR6968100 | + | **-** | XM_001980780 |
|  | **264-284 (2689-2709)** | **296 (2740)** |  | **266 (2695)** |
| *D. yakuba* | SRR9678418, SRR9678474, SRR1560493 | **-** | **-** | XM_002096914 |
|  | **244-275 (2666-2701)** |  |  | **269 (2695)** |
| *D. sechellia* | DRR164933, SRR10197235, SRR10197232 | + | **-** | XM_002031855 |
|  | **254, 341 (2685, 2804)** | **199-212 (2629-2642)** |  | **266 (2701)** |
| *D. simulans* | ERR4471022 | + | **-** | XM_016177119 |
|  | **253 (2684)** | **234-277 (2665-2712)** |  | **213 (2643)** |
| *D. melanogaster* | DRR016399, SRR14127352, SRR14470605 | + | GHHI01001131, JV230655 | NM_057351 |
|  | **246-272 (2677-2707)** | **223-246 (2654-2677)** | **234-256 (2665-2687)** | **260 (2695)** |
| *D. ficusphila* | SRR346750, SRR346728 | **-** | **-** | XM_017191603, XM_017191604 |
|  | **244-273 (2682-2728)** |  |  | **242 (2680)** |
| *D. rhopaloa* | SRR346755 | **-** | **-** | XM_017120230 |
|  | **283 (2687)** |  |  | **280 (2684)** |
| *D. biarmipes* | SRR346719 | **-** | GHHN01002114, GHHN01002115, GHHN01002116 | XM_017094926 |
|  | **269-280 (2700-2712)** |  | **296 (2728)** | **269 (2700)** |
| *D. takahashii* | SRR346757, SRR346756 | **-** | **-** | XM_017147175 |
|  | **265 (2720)** |  |  | **238 (2686)** |
| *D. eugracilis* | SRR346728 | **-** | **-** | XM_017218278, XM_017218287 |
|  | **80-128 (2639-2714)** |  |  | **107 (2691)** |
| *D. elegans* | SRR346726 | **-** | **-** | XM_017262065, XM_017262066 |
|  | **614-649 (2665-2705)** |  |  | **639 (2695)** |
| *D. suzukii* | SRR1002989 |  | GHHJ01003075, GHHJ01012914-GHHJ01012916, GHHJ01012919-GHHJ01012930, GHHJ01015441, GHHJ01015442, GHHJ01017099, GHHJ01017101, GHHJ01022270, GHHJ01025809, GHHJ01025810, GHHJ01029748, GHHJ01029750, GHHJ01033436 | XM_017084544, XM_036818393 |
|  | **525-551 (2595-2625)** |  | **222-263 (2292-2333), 497 (2567)** | **743 (2896)** |
| *D. pseudo-obscura* | DRR003873 | **+** | **-** | XM_001359398 |
|  | **17-41 (2635-2675)** | **135 (2864)** |  | **23 (2641)** |
| *D. persimilis* | SRR6181055, SRR7243863, SRR7243873 | **-** | **-** | XM_002017247 |
|  | **85-124 (2774-2826)** |  |  | **108 (2799)** |
| *D. obscura* | DRR055215, DRR055231, DRR055207 | **-** | **-** | XM_022357755 |
|  | **602-626 (2665-2691)** |  |  | **600 (2663)** |
| *D. serrata* | SRR5481716, SRR5481719, SRR5481723 | **+** | **-** | XM_020953174 |
|  | **16-33 (2673-2698)** | **20 (2677)** |  | **1 (2637)** |
| *D. ananassae* | SRR166824 | **+** | **-** | XM_001955758 |
|  | **443 (2682)** | **449 (2688)** |  | **411 (2640)** |
| *D. bipectinata* | SRR346722 | **-** | **-** | XM_017235881, XM_017235882 |
|  | **438-457(2674-2696)** |  |  | **435 (2671)** |
| *D. kikkawai* | SRR346730 | **-** | **-** | XM_017164810 |
|  | **194-212(2667-2686)** |  |  | **191 (2652)** |
| *D. willistoni* | SRR7243913, SRR7243914, SRR341132 | **+** | **-** | XM_023180102, XM_002072843 |
|  | **330-371 (2684-2728)** | **480 (2946)** |  | **377, 461 (2744, 2926)** |
| *D. busckii* | SRR1805120, SRR1804796 | **-** | **-** | XM_017989628, XM_017989629 |
|  | **477 (2675)** |  |  | **461, 466 (2659, 2664)** |
| *D. albomicans* | SRR10075789, SRR10875322, SRR4416169 | **-** | **-** | XM_034261207 |
|  | **319-352 (2653-2686)** |  |  | **397 (2736)** |
| *D. hydei* | SRR6039975, SRR7362824, SRR7362821 | **-** | **-** | XM_030222830, XM_030222831, XM_030222832 |
|  | **233-262 (2702-2732)** |  |  | **207, 315 (2674, 2791)** |
| *D. grimshawi* | SRR7253572, SRR3355288, SRR7253581 | **+** | **-** | XM_032737006, XM_032737007, XM_032737008 |
|  | **317 (2687)** | **358-382 (2732-2759)** |  | **378 (2755)** |
| *D. navojoa* | SRR7973857, SRR7973858 | **-** | **-** | XM_018112610 |
|  | **317-349 (2697-2731)** |  |  | **315 (2695)** |
| *D. mojavensis* | SRR11678586 | **+** | **-** | XM_002000267 |
|  | **311-330 (2695-2714)** | **323 (2707)** |  | **303 (2685)** |
| *D. montana* | SRR10960337, SRR2910692 | **-** | GECM01028457 | **-** |
|  | **1329-1349 (2579-2599)** |  | **1337 (2587)** |  |
| *D. americana* | SRR5279019, SRR5279026 | **-** | **-** | **-** |
|  | **1339 (2682)** |  |  |  |
| *D. virilis* | SRR5839427, SRR5278994, SRR2096049, SRR2096035 | **+** | **-** | XM_015171938, XM_015171939 |
|  | **1159 (2688)** | **1155 (2684)** |  | **1149, 1173 (2678, 2704)** |

**Supplementary Table S5. Positions of the transcription start sites of the *ras85D* gene and its orthologs in drosophila species of the subgenera *Sophophora*, *Drosophila*, and *Dorsilopha*, obtained by analyzing data from public Entrez SRA, EST, TSA, Nucleotide databases**. The accession numbers of the analyzed libraries for data from SRA and TSA databases are given. The TSSs listed in the table mark the beginning of the area of ​​continuous coverage with reads identified by the analysis of the SRA and TSA libraries. TSS positions are represented by the b.p. from the distal end of the intergenic spacer; the positions on the general alignment is shown in parentheses. The hyphenated double positions mark an area of significant increase in the number of reads downstream of the beginning of the area of ​​continuous coverage with reads.

| Species | Promoter position | Promoter element | Alternative ID | Control group | Chein | p-val. | E-val. | True positive |
| --- | --- | --- | --- | --- | --- | --- | --- | --- |
| *melano-gaster* group | Main  2627-2747 | Ohler7 | DBKBNCA- DCHCTRDY | Spacer | + | 1.25e-5 | 1.25e-5 | 9(81.8%) |
|  |  |  |  | Shuffled | + | 9.85e-15 | 9.85e-15 | 8(80%) |
|  |  | DRE | NNHWATC- GATANNN | Spacer | + | 1.34e-6 | 1.34e-6 | 10(100%) |
|  |  |  |  | Shuffled | + | 1.12e-15 | 1.12e-15 | 9(90%) |
|  |  | BRE^u^ | SSRCGCC | Spacer | + | 1.80e-2 | 2.34e-1 | 4(40%) |
|  |  | INR | TCAKTGY | Spacer | + | 7.26e-1 | 9.44e0 | 5(50%) |
|  |  | DPE | VHRSWY-VNN | Spacer | **+** | 9.21e-1 | 1.20e1 | 5 (50.0%) |
|  |  |  |  | Shuffled | **+** | 1.00e0 | 1.30e1 | 5 (50.0%) |
| *montium, ananas-sae, obscura* groups | conserv. seq.4 2718-2860 | CCAAT-box | HNNRRCCAATSR | Spacer | + | 7.17e-1 | 9.32e0 | 1(12.5%) |
|  |  | TCT* | YTCTTTT | Spacer | + | 1.13e-1 | 1.13e-1 | 3(37.5%) |
|  |  |  |  | Shuffled | + | 3.52e-1 | 3.52e-1 | 3(37.5%) |
|  |  | Inr | HTCAGTY | Spacer | + | 1.00e0 | 1.00e0 | 2(25.0%) |
|  |  |  |  | Shuffled | + | 1.00e0 | 1.00e0 | 2(25.0%) |
|  |  | BRE^d^ | RTDKKKK | Spacer | + | 1.00e0 | 1.00e0 | 5(62.5%) |
|  |  |  |  | Shuffled | + | 5.81e-1 | 7.55e0 | 5(62.5%) |
|  |  | TATA+ CCAAT | ACGAATTTATTGTTT | Xstreme | + | Streme E-value 1.9e-022 | | 8(100%) |
|  |  | CCAAT +Inr | TTYTTWRRTGGTAAT | Xstreme | + | Streme E-value 1.2e-005 | | 8(100%) |
|  | Main  2620-2717 | Inr | TCAKTGY | Spacer | + | 4.90e-1 | 6.37e0 | 6(85.7%) |
|  |  |  |  | Shuffled | + | 9.94e-1 | 1.29e1 | 4(57.1%) |
|  |  | DRE | WATCGATA | Spacer | + | 1.20e-1 | 1.20e-1 | 2(28.6%) |
|  |  |  |  | Shuffled | + | 4.15e-1 | 4.15e-1 | 2(28.6%) |
|  |  | DPE | VHRSWY-VNN | Spacer | + | 6.47e-2 | 8.41e-1 | 6(85.7%) |
|  |  |  |  | Shuffled | + | 1.00e0 | 1.30e1 | 4(57.1%) |
|  |  | CCAAT-box | HNNRRCCAATSR | Spacer | + | 1.00e0 | 1.00e0 | 5 (71.4%) |
|  |  |  |  | Shuffled | + | 1.00e0 | 1.00e0 | 5 (71.4%) |
|  |  | Ohler7 | DBKBNCA- DCHCTRDY | Spacer | + | 8.38e-3 | 8.38e-3 | 3(42.9%) |
|  |  |  |  | Shuffled | + | 1.69e-5 | 1.69e-5 | 3(42.9%) |
|  |  | Ohler1 | NVNVYGG-TCACACTR | Spacer | + | 2.62e-3 | 2.62e-3 | 4 (57.1%) |
|  |  |  |  | Shuffled | + | 1.04e-2 | 1.04e-2 | 4 (57.1%) |
|  |  | TATA+Inr | CAATATRTAT | Xstreme | + | Streme E-value 3.5e-002 | | 4 (57.1%) |
|  |  | CCAAT-box | AGCCAAAATGGCGAC | Xstreme | + | Streme E-value 7.0e-014 | | 6(85.7%) |
| *obscura* group | conserv. seq.4 2718-2860 | TCT* | TTCTTTTA | Spacer | **+** | 1.26e-4 | 1.26e-4 | 3(100%) |
|  |  |  |  | Shuffled | **+** | 4.41e-4 | 4.41e-4 | 3(100%) |
|  |  | Inr* | TTAGTGG | Spacer | **+** | 1.22e-2 | 1.22e-2 | 2(66.7%) |
|  |  |  |  | Shuffled | **+** | 1.80e-3 | 1.80e-3 | 2(66.7%) |
|  |  | Ohler7 | DBKBNCA- DCHCTRDY | Spacer | + | 5.93e-2 | 5.93e-2 | 2(66.7%) |
|  |  |  |  | Shuffled | + | 4.82e-4 | 4.82e-4 | 2(66.7%) |
| s/gen. *Drosophila* | Main without conserv. seq.4  2619-2726 | Inr | TCAKTGY | Spacer | + | 9.19e-6 | 1.19e-4 | 13(86.7%) |
|  |  |  |  | Shuffled | + | 4.27e-3 | 5.54e-2 | 13(86.7%) |
|  |  | DRE | HWATCG-ATA | Spacer | - | 7.89e-1 | 7.89e-1 | 9(60.0%) |
|  |  |  |  | Shuffled | - | 4.17e-5 | 4.17e-5 | 8(53.3%) |
|  |  | DPE** | KCGGTTSK | Spacer | - | 1.00e0 | 1.00e0 | 1(6.7%) |
|  |  |  |  | Spacer** | - | 5.19e-5 | 6.75e-4 | 9(60.0%) |
|  |  | TATA* | TATAAAAR | Spacer | + | 1.00e0 | 1.00e0 | 2 (13.3%) |
|  |  |  |  | Shuffled | + | 1.00e0 | 1.00e0 | 2 (13.3%) |
|  |  | Ohler7 | DBKBNCA- DCHCTRDY | Spacer | - | 1.25e-15 | 1.25e-15 | 13(86.7%) |
|  |  |  |  | Shuffled | - | 2.74e-17 | 2.74e-17 | 13(86.7%) |
|  |  | Ohler6 | WKBYGGT-ATTTTTHV | Spacer | +/- | 9.95e-1 | 9.95e-1 | 4(26.7%) |
|  |  |  |  | Shuffled | +/- | 9.95e-1 | 9.95e-1 | 2(13.3%) |
|  | grimshavi, replete gr., downstream TSS  2764-2866 | Ohler1 | NVNVYGG-TCACACTR | Spacer | + | 1.00e0 | 1.00e0 | 1(25.0%) |
|  |  | Ohler6* | BYGGTATTTTT | Spacer | +/- | 5.46e-1 | 5.46e-1 | 3(75.0%) |
|  |  | Ohler10 | CVAMCSVAACGV | Spacer | + | 1.00e0 | 1.00e0 | 3(75.0%) |
|  |  | Inr** | HTCAGTYB | Spacer** |  | 1.00e0 | 1.00e0 | 3(75.0%) |
|  | Main + conserv. seq.4  2619-2782 | INR | TCAKTGY | Spacer | + | 3.14e-7 | 4.09e-6 | 16(100%) |
|  |  |  |  | Shuffled | + | 1.00e0 | 1.30e1 | 16(100%) |
|  |  | DPE | VHRSWY-VNN | Spacer | + | 6.91e-6 | 8.98e-5 | 11(68.8%) |
|  |  |  |  | Shuffled | + | 8.73e-1 | 1.14e1 | 10(62.5%) |
|  |  | CCAAT-box | AGCCAAAATGGCGAC | Spacer | + | 9.80e-6 | 1.27e-4 | 14(87.5%) |
|  |  |  |  | Shuffled | + | 5.25e-2 | 6.83e-1 | 14(87.5%) |
|  |  | BRE^d^ | RTDKKKK | Spacer | + | 2.01e-5 | 2.61e-4 | 15(93.8%) |
|  |  |  |  | Shuffled | + | 1.00e0 | 1.30e1 | 15(93.8%) |
|  |  | Ohler6 | WKBYGGT-ATTTTTHV | Spacer | +/- | 1.13e-9 | 5.67e-9 | 15(93.8%) |
|  |  |  |  | Shuffled | +/- | 6.93e-8 | 3.47e-7 | 15(93.8%) |
|  |  | Ohler7 | DBKBNCA- DCHCTRDY | Spacer | - | 5.37e-4 | 2.68e-3 | 16(100%) |
|  |  |  |  | Shuffled | - | 1.77e-4 | 8.85e-4 | 16(100%) |
|  |  | DRE | HWATCG-ATA | Spacer | +/- | 2.73e-1 | 1.36e0 | 9(56.3%) |
|  |  |  |  | Shuffled | +/- | 9.40e-5 | 4.70e-4 | 9(56.3%) |
| *D. buscii* | Promoter 2602-2712 | Ohler6 | WKBYGGT-ATTTTTHV | Shuffled | +/- | 1.00e0 | 5.00e0 | 1(100.0%) |
|  |  | CCAAT-box | HNNRRCCAATSR | Shuffled | +/- | 9.97e-1 | 1.30e1 | 1(100.0%) |
|  |  | BRE^d^ | RTDKKKK | Shuffled | + | 1.00e0 | 1.30e1 | 1(100.0%) |
|  |  | TATA-Box | STATAAAARS | Shuffled | - | 1.00e0 | 1.30e1 | 1(100.0%) |

**Supplementary Table S6. Composition and enrichment of the TSS region of the ras85D gene with promoter elements in the analyzed Drosophila species.** The algorithms of the analytical platform MEME Suite 5.4.1 were used. * - used the core of the sequence; ** - 'PWM score' assigned to a sequence is a “total hits”, in all other cases 'PWM score' is an “Average odds score”. In column “control group”: Spacer - control sequences were obtained by cutting fragments of the upstream intergenic spacer of the corresponding Drosophila species; Shuffled - control sequences are generated by random permutations based on the composition of the analyzed sequences. In both cases, the AME algorithm is applied. Xstreme - to analyze small and more heterogeneous samples of promoter sequences in the obscura, ananassae, and montium groups, the Xstreme algorithm was used (Grant, Bailey, 2021). The column "Chein" indicates the position of the identified motifs on the "+" or "-" DNA strands. The column "True positive" contains the absolute and relative number of sequences with identified motifs. The assessments of the presence and enrichment were obtained for the following elements: TATA-Box, CCAAT-box, BREu, BREd, Inr, TCT, DPE, Ohler1, Ohler6, Ohler7, Ohler10, DRE. Assessments were calculated for each group of sequences with a common evolutionary origin and a similar TSS pattern. Two sets of sequences were used as controls. The first set was obtained by cutting fragments of similar length from the upstream region of the intergenic spacer used in the sequence analysis. The second set was generated by random permutations based on the composition of the analyzed sequences. In the first case, the hypothesis about the random distribution of promoter elements in the entire region of the intergenic spacer was tested. In the second case, the hypothesis about the random distribution of the identified elements in the promoter region was tested. The control sequences generated only by random permutations were used for single sequences not included in any of the groups.

| conserv.seq. | GO | Overlap | Adj.p-val. | Z-score | Genes |
| --- | --- | --- | --- | --- | --- |
| 2A/1B | nuclear division (GO:0000280) | 6/623 | 5.8E-08 | -1.05 | D;kni; disco; lola; Med;Trl |
|  | **positive regulation of nucleic acid-templated transcription (GO:1903508)** | 6/630 | 5.8E-08 | -1.04 | D;kni; disco;lola; Med;Trl |
|  | negative regulation of salivary gland boundary specification (GO:0045705) | 5/458 | 1.1E-06 | -1.02 | D;kni; disco;lola; Med |
|  | regulation of salivary gland boundary specification (GO:0045704) | 4/204 | 3.8E-06 | -1.22 | D;disco; lola;Trl |
|  | regulation of cellular macromolecule biosynthetic process (GO:2000112) | 3/161 | 0.0001 | -1.02 | D;Med;Trl |
|  | branched duct epithelial cell fate determination, open tracheal system (GO:0046845) | 3/170 | 0.0001 | -0.90 | disco;Med; Trl |
|  | **positive regulation of phosphorylation of RNA polymerase II C-terminal domain (GO:1901409)** | 3/240 | 0.0003 | -1.13 | lola;Med; Trl |
|  | **positive regulation of gene expression (GO:0010628)** | 3/242 | 0.0003 | -1.05 | D;lola;Med |
|  | **positive regulation of chromatin organization (GO:1905269)** | 2/34 | 0.0004 | -1.74 | D;kni |
|  | cellular response to BMP stimulus (GO:0071773) | 3/279 | 0.0005 | -1.03 | D;kni;Trl |
|  | blastoderm segmentation (GO:0007350) | 2/44 | 0.0005 | -1.67 | D;disco |
|  | eclosion rhythm (GO:0008062) | 2/47 | 0.0006 | -1.59 | D;kni |
|  | retinal ganglion cell axon guidance (GO:0031290) | 2/54 | 0.0007 | -1.55 | D;Med |
|  | trunk segmentation (GO:0035290) | 2/56 | 0.0007 | -1.51 | D;Med |
| 1A/2B | leg disc morphogenesis (GO:0007478) | 3/61 | 1.7E-05 | -1.35 | bab1;Dll; Ubx |
|  | imaginal disc-derived genitalia development (GO:0007484) | 2/10 | 6.9E-05 | -2.92 | Dll;en |
|  | imaginal disc-derived appendage development (GO:0048737) | 2/15 | 0.0001 | -2.94 | Dll;Ubx |
|  | imaginal disc-derived male genitalia development (GO:0007485) | 2/26 | 0.0002 | -2.14 | Dll;en |
|  | genital disc development (GO:0035215) | 2/25 | 0.0002 | -1.98 | Dll;en |
|  | imaginal disc-derived wing morphogenesis (GO:0007476) | 3/240 | 0.0002 | -1.14 | Dll;fru;en |
|  | anterior/posterior pattern specification (GO:0009952) | 2/46 | 0.0006 | -1.36 | en;Ubx |
|  | eye-antennal disc development (GO:0035214) | 2/54 | 0.0008 | -1.82 | bab1;Dll |
|  | imaginal disc-derived leg morphogenesis (GO:0007480) | 2/68 | 0.0012 | -1.42 | Dll;Ubx |
|  | negative regulation of macromolecule metabolic process (GO:0010605) | 2/73 | 0.0012 | -1.68 | Dll;en |
| 3A/3B/12A | **positive regulation of transcription from RNA polymerase II promoter (GO:0045944)** | 4/305 | 9.8E-05 | -1.08 | twi;ato; da;Trl |
|  | **positive regulation of transcription, DNA-templated (GO:0045893)** | 4/379 | 0.0002 | -1.01 | twi;ato; da;Trl |
|  | R8 cell fate commitment (GO:0007460) | 2/14 | 0.0003 | -3.18 | h;ato |
|  | R8 cell differentiation (GO:0045465) | 2/16 | 0.0003 | -2.97 | h;ato |
|  | oogenesis (GO:0048477) | 3/216 | 0.0009 | -1.10 | lov;da;Trl |
|  | nervous system development (GO:0007399) | 3/246 | 0.0010 | -1.08 | h;ato;da |
|  | compound eye photoreceptor fate commitment (GO:0001752) | 2/41 | 0.0013 | -1.57 | h;ato |
|  | neuron differentiation (GO:0030182) | 2/45 | 0.0014 | -1.76 | Med;da |
|  | circulatory system development (GO:0072359) | 2/55 | 0.0016 | -1.70 | twi;Med |
|  | gland morphogenesis (GO:0022612) | 2/55 | 0.0016 | -1.63 | twi;h |
|  | dorsal/ventral axis specification (GO:0009950) | 2/54 | 0.0016 | -1.55 | twi;Med |
|  | dorsal/ventral pattern formation (GO:0009953) | 2/56 | 0.0016 | -1.51 | twi;Med |
| 4A/4B/13B | **negative regulation of gene expression (GO:0010629)** | 6/245 | 3.1E-08 | -1.05 | Dll;pnr;pho; kni;en;ttk |
|  | **negative regulation of nucleic acid-templated transcription (GO:1903507)** | 5/132 | 1.1E-07 | -1.42 | pnr;pho; kni; en;ttk |
|  | **positive regulation of transcription, DNA-templated (GO:0045893)** | 6/379 | 2.9E-07 | -1 | Dll;pnr;pho; en;lola;ttk |
|  | negative regulation of cellular macromolecule biosynthetic process (GO:2000113) | 5/179 | 3.7E-07 | -1.41 | pnr;pho; kni; en;ttk |
|  | **negative regulation of transcription from RNA polymerase II promoter (GO:0000122)** | 5/192 | 4.6E-07 | -1.18 | sens;pnr; kni;en;Ubx |
|  | **positive regulation of gene expression (GO:0010628)** | 5/204 | 5.3E-07 | -1.21 | sens;Dll;pnr; lola;Ubx |
|  | regulation of gene expression (GO:0010468) | 6/458 | 5.3E-07 | -1.01 | Dll;pho;kni; en;lola;Ubx |
|  | blastoderm segmentation (GO:0007350) | 3/34 | 9.5E-06 | -1.75 | pnr;kni;en |
|  | segmentation (GO:0035282) | 3/47 | 2.4E-05 | -1.59 | pnr;kni;en |
|  | negative regulation of macromolecule metabolic process (GO:0010605) | 3/73 | 7.7E-05 | -1.68 | Dll;pho;en |
|  | regulation of cellular macromolecule biosynthetic process (GO:2000112) | 4/266 | 7.9E-05 | -1.36 | pho;kni; lola;Ubx |
|  | compound eye photoreceptor development (GO:0042051) | 3/78 | 8.2E-05 | -1.36 | sens;lola; ttk |
|  | imaginal disc-derived genitalia development (GO:0007484) | 2/10 | 0.0001 | -2.9 | Dll;en |
|  | trunk segmentation (GO:0035290) | 2/11 | 0.0001 | -2.9 | kni;en |
|  | imaginal disc-derived appendage development (GO:0048737) | 2/15 | 0.0002 | -2.88 | Dll;Ubx |
|  | **positive regulation of nucleic acid-templated transcription (GO:1903508)** | 3/135 | 0.0003 | -1.42 | Dll;pnr;lola |
| 5A/5B | **positive regulation of nucleic acid-templated transcription (GO:1903508)** | 4/135 | 1.2E-06 | -1.45 | pnr;dl; disco; lola |
|  | **positive regulation of gene expression (GO:0010628)** | 4/204 | 4.7E-06 | -1.22 | pnr;dl; disco; lola |
|  | **positive regulation of transcription, DNA-templated (GO:0045893)** | 4/379 | 4.5E-05 | -1.01 | pnr;dl; disco; lola |
|  | **negative regulation of nucleic acid-templated transcription (GO:1903507)** | 3/132 | 9.7E-05 | -1.42 | pnr;kni;dl |
|  | negative regulation of cellular macromolecule biosynthetic process (GO:2000113) | 3/179 | 0.0002 | -1.41 | pnr;kni;dl |
|  | **negative regulation of transcription from RNA polymerase II promoter (GO:0000122)** | 3/192 | 0.0002 | -1.17 | pnr;kni;dl |
|  | ectoderm development (GO:0007398) | 2/31 | 0.0004 | -1.73 | pnr;dl |
|  | **negative regulation of gene expression (GO:0010629)** | 3/245 | 0.0004 | -1.04 | pnr;kni;dl |
|  | blastoderm segmentation (GO:0007350) | 2/34 | 0.0004 | -1.74 | pnr;kni |
|  | regulation of cellular macromolecule biosynthetic process (GO:2000112) | 3/266 | 0.0004 | -1.36 | kni;dl;lola |
|  | **negative regulation of transcription, DNA-templated (GO:0045892)** | 3/279 | 0.0004 | -1.03 | pnr;kni;dl |
|  | **positive regulation of transcription from RNA polymerase II promoter (GO:0045944)** | 3/305 | 0.0005 | -1.06 | pnr;dl; disco |
|  | segmentation (GO:0035282) | 2/47 | 0.0005 | -1.58 | pnr;kni |
|  | lymph gland development (GO:0048542) | 2/47 | 0.0005 | -1.55 | pnr;lola |
|  | circulatory system development (GO:0072359) | 2/55 | 0.0007 | -1.69 | pnr;dl |
|  | heart development (GO:0007507) | 2/87 | 0.0017 | -1.27 | pnr;dl |
|  | blood circulation (GO:0008015) | 1/6 | 0.0100 | -3.19 | pnr |
|  | **histone H3-K9 methylation (GO:0051567) - silenced transcription** | 1/7 | 0.0111 | -3.43 | BEAF-32 |
|  | peripheral nervous system neuron differentiation (GO:0048934) | 1/9 | 0.0115 | -3.87 | dl |
| 7A/8B/9B | Malpighian tubule development (GO:0072002) | 3/61 | 1.5E-05 | -1.11 | sc;twi;da |
|  | **positive regulation of transcription, DNA-templated (GO:0045893)** | 4/379 | 2.3E-05 | -1.01 | sc;twi; lola;da |
|  | Malpighian tubule tip cell differentiation (GO:0061382) | 2/10 | 4.9E-05 | -3.82 | sc;da |
|  | neuroblast fate commitment (GO:0014017) | 2/31 | 0.0002 | -2.43 | sc;da |
|  | neuroblast fate determination (GO:0007400) | 2/27 | 0.0002 | -1.88 | sc;da |
|  | stem cell fate determination (GO:0048867) | 2/32 | 0.0002 | -1.55 | sc;da |
|  | regulation of cellular macromolecule biosynthetic process (GO:2000112) | 3/266 | 0.0002 | -1.37 | sc;kni;lola |
|  | **positive regulation of transcription from RNA polymerase II promoter (GO:0045944)** | 3/305 | 0.0003 | -1.07 | sc;twi;da |
|  | chaeta morphogenesis (GO:0008407) | 2/42 | 0.0004 | -1.98 | sc;da |
|  | peripheral nervous system development (GO:0007422) | 2/79 | 0.0012 | -1.39 | sc;da |
|  | chaeta development (GO:0022416) | 2/85 | 0.0013 | -1.35 | sc;da |
|  | regulation of mitotic cell cycle (GO:0007346) | 2/100 | 0.0017 | -1.25 | sc;kni |
|  | **positive regulation of nucleic acid-templated transcription (GO:1903508)** | 2/135 | 0.0029 | -1.42 | lola;da |
|  | sensory organ development (GO:0007423) | 2/143 | 0.0030 | -1.15 | sc;da |
| 4A/7B | **positive regulation of gene expression (GO:0010628)** | 6/204 | 3.5E-09 | -1.22 | sens;D; Dll;disco; lola;Ubx |
|  | leg disc morphogenesis (GO:0007478) | 4/61 | 3.2E-07 | -1.35 | bab1;Dll; Ubx;Med |
|  | axon guidance (GO:0007411) | 5/242 | 7.7E-07 | -1.05 | sens;D;en; lola;Med |
|  | regulation of cellular macromolecule biosynthetic process (GO:2000112) | 5/266 | 1.0E-06 | -1.37 | bab1;D; lola;Ubx;Med |
|  | **positive regulation of nucleic acid-templated transcription (GO:1903508)** | 4/135 | 4.5E-06 | -1.44 | D;Dll; disco;lola |
|  | **positive regulation of transcription, DNA-templated (GO:0045893)** | 5/379 | 4.6E-06 | -1.00 | D;Dll; disco; en; lola |
|  | post-embryonic appendage morphogenesis (GO:0035120) | 4/161 | 7.4E-06 | -1.02 | D;Dll; Ubx;Med |
|  | imaginal disc-derived appendage morphogenesis (GO:0035114) | 4/170 | 8.4E-06 | -0.90 | Dll;disco; Ubx;Med |
|  | **negative regulation of transcription from RNA polymerase II promoter (GO:0000122)** | 4/192 | 1.3E-05 | -1.17 | sens;D; en;Ubx |
|  | eye-antennal disc development (GO:0035214) | 3/54 | 1.7E-05 | -1.81 | bab1;Dll; disco |
|  | axonogenesis (GO:0007409) | 4/212 | 1.7E-05 | -1.07 | sens;D; en;lola |
|  | dorsal/ventral pattern formation (GO:0009953) | 3/56 | 1.8E-05 | -1.51 | D;Dll;Med |
|  | imaginal disc-derived wing morphogenesis (GO:0007476) | 4/240 | 2.4E-05 | -1.12 | Dll;en; lola;Med |
|  | imaginal disc-derived leg morphogenesis (GO:0007480) | 3/68 | 2.9E-05 | -1.41 | Dll;Ubx; Med |
|  | **negative regulation of transcription, DNA-templated (GO:0045892)** | 4/279 | 3.8E-05 | -1.02 | sens;D; en;Ubx |
| 13A/11B | **positive regulation of nucleic acid-templated transcription (GO:1903508)** | 3/135 | 2.4E-05 | -1.46 | D;pnr;lola |
|  | **positive regulation of gene expression (GO:0010628)** | 3/204 | 4.2E-05 | -1.22 | D;pnr;lola |
|  | blastoderm segmentation (GO:0007350) | 2/34 | 0.0002 | -1.75 | D;pnr |
|  | **positive regulation of transcription, DNA-templated (GO:0045893)** | 3/379 | 0.0002 | -1.01 | D;pnr;lola |
|  | segmentation (GO:0035282) | 2/47 | 0.0002 | -1.60 | D;pnr |
|  | lymph gland development (GO:0048542) | 2/47 | 0.0002 | -1.56 | pnr;lola |
|  | dendrite morphogenesis (GO:0048813) | 2/141 | 0.0013 | -1.18 | D;lola |
|  | **negative regulation of transcription from RNA polymerase II promoter (GO:0000122)** | 2/192 | 0.0022 | -1.17 | D;pnr |
|  | axonogenesis (GO:0007409) | 2/212 | 0.0025 | -1.07 | D;lola |
|  | regulation of cellular macromolecule biosynthetic process (GO:2000112) | 2/266 | 0.0030 | -1.36 | D;lola |
|  | blood circulation (GO:0008015) | 1/6 | 0.0043 | -3.21 | pnr |
|  | cardioblast cell fate commitment (GO:0042684) | 1/8 | 0.0051 | -3.55 | pnr |
|  | heart contraction (GO:0060047) | 1/8 | 0.0051 | -1.39 | pnr |
|  | digestive system development (GO:0055123) | 1/9 | 0.0055 | -3.61 | pnr |

**Supplementary Table S7. GO enrichment analysis of gene sets specific for evolutionary conservative sequences.** GO Biological Process ## GO: 0006355, GO: 0006357, GO: 0010468, GO: 0045892, GO: 0071824, GO: 1903506 associated with general regulation of transcription are excluded from the lists as obvious for sets of exclusively transcription factors. GO processes that determine an increase or decrease in transcriptional activity are highlighted in bold.
